# Quantitative measures of clock protein dynamics in the mouse suprachiasmatic nucleus extends the circadian time-keeping model

**DOI:** 10.1038/s44318-025-00426-z

**Published:** 2025-04-17

**Authors:** Nicola J Smyllie, Alex A Koch, Antony D Adamson, Andrew P Patton, Adam Johnson, James S Bagnall, Olivia Johnson, Qing-Jun Meng, Andrew S I Loudon, Michael H Hastings

**Affiliations:** 1 Neurobiology Division, MRC Laboratory of Molecular Biology, Cambridge, UK; 2 Faculty of Biology, Medicine and Health, University of Manchester, Manchester, UK

**Keywords:** Suprachiasmatic Nucleus, PER2, CRY1, TTFL, E-box, Chromatin, Transcription & Genomics, Neuroscience, Post-translational Modifications & Proteolysis

## Abstract

The suprachiasmatic nucleus (SCN) synchronises circadian rhythmicity (~24 h) across the body. The SCN cell-autonomous clock is modelled qualitatively as a transcriptional-translational feedback loop (TTFL), with heteromeric complexes of transcriptional activator and repressor proteins driving cyclical gene expression. How these proteins really behave within the SCN, individually and in relation to each other, is poorly understood. Imaging SCN slices from a novel array of knock-in reporter mice, we quantify the dynamic behaviours of combined repressors PERIOD2 (PER2) and CRYPTOCHROME1 (CRY1), and activator BMAL1. We reveal a spectrum of protein-specific intracellular and spatiotemporal behaviours that run counter to the qualitative TTFL model. We also show that PER and CRY1 exert independent actions on TTFL oscillations, and that their individual stabilities play a critical role in SCN circadian dynamics. These results reveal a rich and unanticipated complexity in the dynamic behaviours and functions of endogenous circadian proteins, prompting re-appraisal of current transcriptional-translational feedback loop models of the suprachiasmatic nucleus.

## Synopsis

The intracellular dynamics of clock proteins are fundamental to circadian time-keeping in the mammalian suprachiasmatic nucleus (SCN), but they are poorly understood. This study provides new quantitative insights into their spatio-temporal relationships that expand the current model of the clock.

- Circadian clock proteins PER2, CRY1, and BMAL1, imaged as knock-in fluorescent fusions, exhibit a spectrum of spatio-temporal behaviours in the mouse suprachiasmatic nucleus.
- In contrast to the current clock model where PER2 and CRY1 are depicted in the same place and time, in complex, we show that they are in fact segregated in circadian time and in SCN cellular space.
- A new mouse model enables quantification of relative molecular abundances of CRY1, BMAL1, and PER2 across the circadian day, showing that CRY1 and BMAL1 levels match or exceed those of PER2.
- PER2 emerges as the limiting factor for complex formation with CRY1 or BMAL1.
- Pharmacological stabilisation of PERs or CRY1 reveals their independence, with no mutual stabilisation, and their different and additive effects on SCN clock dynamics.

## Abstract (web summary)

The complex and dynamic behaviours of PER2, CRY1, and BMAL1 prompt re-appraisal of current transcriptional-translational feedback loop models.

## Graphical abstract

**Figure:**
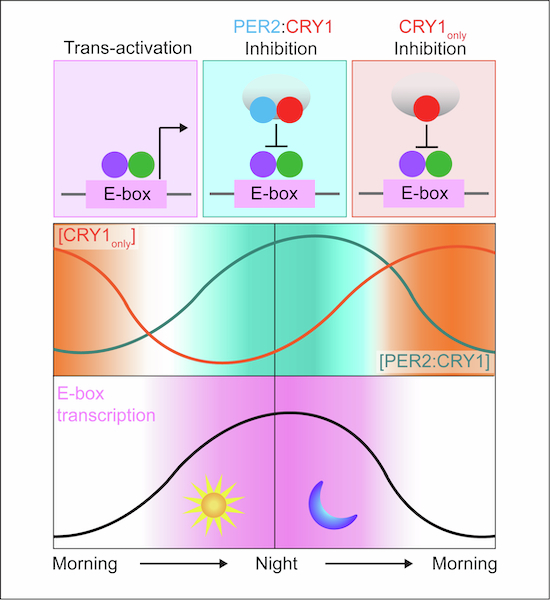

**Figure:**
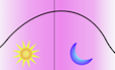

## Introduction

Circadian (~24 h) timekeeping allows organisms to schedule their biology in anticipation of the demands and opportunities of the 24-h world (Dunlap et al, 2004). In mammals, the suprachiasmatic nucleus (SCN) of the hypothalamus sits at the pinnacle of a body-wide circadian network (Reppert and Weaver, 2002). It generates a robust internal representation of solar time, encoded as a circadian cycle of gene expression and electrical activity. This timing signal is conveyed via neural and neuroendocrine links to co-ordinate subordinate cell-autonomous clocks across tissues, thereby orchestrating daily rhythms of physiology and sleep-wakefulness (Hastings et al, 2018). Importantly, whereas circadian rhythms of subordinate clocks in peripheral tissues damp rapidly when isolated from SCN-derived and systemic cues, organotypic SCN slice cultures can maintain autonomous, high-amplitude oscillations for many weeks to months in the absence of systemic cues (Yoo et al, 2004). Understanding the molecular and cellular mechanisms of the SCN time-keeper therefore has broad relevance to health and to diseases associated with modern lifestyles, including shiftwork and ageing, that disrupt circadian clock function (Cederroth et al, 2019).

In both the SCN and peripheral tissues, the cell-autonomous clockwork is modelled as a transcriptional-translational feedback loop (TTFL), incorporating a core set of “clock proteins” arranged within positive and negative regulatory arms (Partch et al, 2014). Heterodimeric BMAL1 and CLOCK trans-activate *Period* (*Per1, Per2*) and *Cryptochrome* (*Cry1, Cry2*) genes via E-box regulatory sequences, while in the negative arm, PER (1, 2) and CRY (1, 2) heteromeric complexes translocate into the nucleus to auto-repress their E-box-mediated trans-activation. Subsequent degradation of PER and CRY alleviates repression, thereby re-initiating trans-activation every ~24 h. Mutations that variously affect the stability and activity of these proteins alter the period of the SCN and circadian behaviour in rodents (Gallego and Virshup, 2007), and are associated with human sleep disorders (Jones et al, 2013).

This model posits that PER and CRY are in the same place, at the same time, doing the same thing. Notwithstanding its success, it is essentially qualitative and heterotypical, broadly based on studies of recombinant proteins in non-neuronal cell lines and “snapshot” analyses of clock proteins in fixed SCN tissues. Such approaches lack the dynamic, quantitative information required to understand SCN timekeeping. Luciferase-based bioluminescence approaches have enabled live-imaging of both transcriptional (Yamaguchi et al, 2016) and translational (Yoo et al, 2004) temporal dynamics of SCN clock proteins. These are, however, indirect enzymatic readouts of protein abundance with limited spatial resolution.

To develop this further, we employed a fluorescent-fusion, multi-channel approach to image SCN clock proteins directly and simultaneously. Organotypic SCN slices from mice carrying novel combinations of fluorescently tagged knock-in (KI) alleles of PER2, CRY1 and BMAL1 were used to visualise, measure and manipulate, pharmacologically, the behaviour of the individual proteins in their native setting. This revealed a spectrum of protein-specific properties that highlight a rich and unanticipated complexity to their dynamic intracellular behaviours. Notably, we show that PER2 and CRY1 do not behave as envisaged and overall, our findings prompt a re-appraisal of the current model of the SCN TTFL.

## Results

### Quantitative comparison of the intracellular behaviour of endogenous clock proteins in the SCN

**Figure 1:**
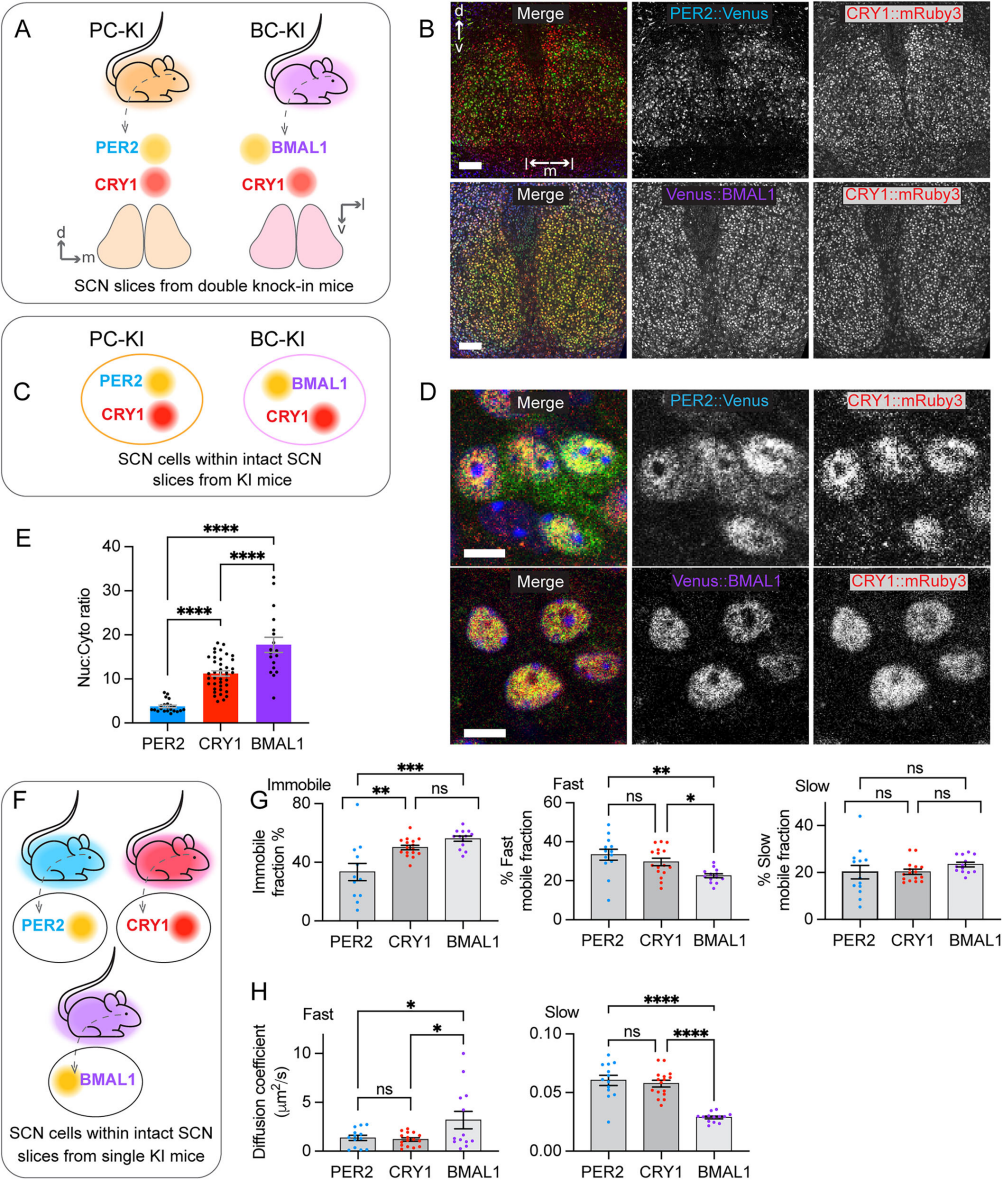
Endogenous circadian clock proteins display different intracellular behaviours within the SCN. (**A**) Schematic describing knock-in (KI) mouse strategy, to accompany images in B. Mice express pairs of fluorescently tagged endogenous clock proteins: either PER2 and CRY1 (PC-KI) or BMAL1 and CRY1 (BC-KI). Anatomical orientation labels (arrows and letters) indicate dorsal (d), medial (m), ventral (v) and lateral (l). (**B**) Confocal images show (upper) PER2::Venus (green) and CRY1::mRuby3 (red) in SCN from PC-KI mice and (lower) CRY1::mRuby3 (red) and Venus::BMAL1 (green) in SCN from BC-KI mice. Counterstained with DAPI (blue). Scale bar = 150 μm. (**C**) Schematic relating to images in (**D**). (**D**) Confocal images show (upper) PER2::Venus (green) and CRY1::mRuby3 (red) in individual cells within fixed SCN slices from PC-KI mice and (lower) CRY1::mRuby3 (red) and Venus::BMAL1 (green) in cells within fixed SCN from BC-KI mice. Counterstained with DAPI (blue). Scale bar = 10 μm. (**E**) Group data show the intracellular localisation metric Nuclear:Cytoplasmic (Nuc:Cyto) ratios of clock proteins within SCN cells (*n* > 170 cells per protein across *n* > 17 SCN per protein; ****P < 0.0001). (**F**) Schematic relating to data in (**G**, **H**). (**G**) Group data show percentages of PER2, CRY1 and BMAL1 molecules that are present within (left) Immobile (ns, *P* = 0.3939, **P = 0.0025, ***P = 0.0001), (middle) Fast (ns, *P* = 0.405, *P = 0.0426, **P = 0.0024), or (right) Slow (ns, *P* > 0.43) intra-nuclear mobility fractions (derived from FRAP curve-fits) within SCN cells. (**H**) Group data show intra-nuclear mobility (diffusion coefficients) of clock proteins within (left) Fast (ns, *P* = 0.9726, *P < 0.0489) or (right) Slow fractions (ns, *P* = 0.7975, ****P < 0.0001). Group data are presented as mean ± SEM, where measurements were made in *n* = 10 cells per SCN and *n* > 12 SCN per group, and analysed using One-way ANOVAs with Tukey’s multiple comparisons tests. Molecular mobility of PER2 and BMAL1 presented in (**G**, **H**) are a meta-analysis of published data (Smyllie et al, 2016; Yang et al, 2020). Images are presented as merge on the left and separate channels (in greyscale) on the right. Source data are available online for this figure.

We intercrossed CRY1::mRuby3 (Koch et al, 2022) mice with either PER2::Venus mice (Smyllie et al, 2016) to create the PC-KI line, or Venus::BMAL1 mice (Yang et al, 2020) to create the BC-KI line. This enabled confocal imaging of spectrally separated pairs of endogenous clock proteins simultaneously within the same living SCN organotypic slice (Fig. 1A,B; Appendix Fig. S1) and in fixed slices (Fig. 1C,D). We compared their intracellular distributions by calculating their nuclear:cytoplasmic (Nuc:Cyto) ratios. The ratios of CRY1 measured in slices from the two KI lines were not different, so the data were combined (Appendix Fig. S2A,B). All three proteins displayed predominantly nuclear localisation, i.e., Nuc:Cyto ratios greater than 1 (Fig. 1E), but the degree of localisation differed between them. PER2 had the lowest ratio (~4) being the least nuclear, BMAL1 had the highest (~18), and CRY1 was intermediate (~11). The ratio was also used to infer the converse: ~27% of PER2, ~9% of CRY1 and ~6% of BMAL1 localised to the cytoplasm. The Nuc:Cyto ratios of PER2 and BMAL1 did not vary with circadian phase, while for CRY1 there was a small, albeit significant, decline limited to circadian time (CT) 12. Nevertheless, even at its nadir, CRY1 was more nuclear than PER2 (Appendix Fig. S2C,D) and overall, the relative degree of nuclear localisation across the three proteins, i.e., BMAL1 > CRY1 > PER2, did not vary as a function of circadian time (Appendix Fig. S2C).

We then used fluorescence recovery after photobleaching (FRAP) to quantify the intra-nuclear mobility of CRY1::mRuby3 (Appendix Fig. S2E–H), and compared it in a meta-analysis (Fig. 1F–H) with published data for PER2 and BMAL1 (Smyllie et al, 2022; Yang et al, 2020). The FRAP curves for CRY1 did not fit to a simple monophasic curve, as its complex recovery dynamics displayed two components. This indicates that, like PER2 and BMAL1, CRY1 molecules form two mobility pools referred to as CRY1_fast_ (~30%), and CRY1_slow_ (~20%) and an “immobile” (or very slowly moving) pool, CRY1_immobile_ (~50%), represented by fluorescence that did not recover over the 90 s course of the experiment. The presence of a slow mobility component may represent molecules present in phase separated liquid droplets, which have been detected for clock proteins REV-ERBα and NCOR1 (Zhu et al, 2023). As with PER2 and BMAL1, the relative sizes of the pools and the mobilities of CRY1 molecules within each pool did not display appreciable circadian differences (Appendix Fig. S2E–H), and so analyses of CRY1 mobility were combined, irrespective of time of day. The size of the PER2_immobile_ pool (~35%) was significantly smaller than either CRY1 _immobile_ (~50%) or BMAL1 _immobile_ (~65%), indicating that proportionally more PER2 molecules were distributed between the mobile pools than either CRY1 or BMAL1 (Fig. 1G). Within the mobile pools, the mobilities of PER2 _fast/slow_ molecules were not significantly different to CRY1 _fast/slow_ (~1 μm^2^s^−1^ and ~0.06 μm^2^s^−1^, respectively), but were different to BMAL1 (~3 μm^2^s^−1^ and ~0.03 μm^2^s^−1^, respectively; Fig. 1H). Overall, the three proteins display different intracellular behaviours: BMAL1 is the most nuclear with a large immobile fraction, which likely represents its predominantly DNA-bound state. CRY1 is also strongly nuclear with a large immobile fraction, consistent with binding to BMAL1, but it also has a significant mobile fraction. PER2 is the most dynamic, with greater cytoplasmic distribution and a smaller immobile pool, but its mobility was matched by that of CRY1, consistent with PER2:CRY1 heterodimerisation.

### Distinct spatiotemporal behaviours of endogenous PER2, CRY1 and BMAL1 in the SCN

**Figure 2:**
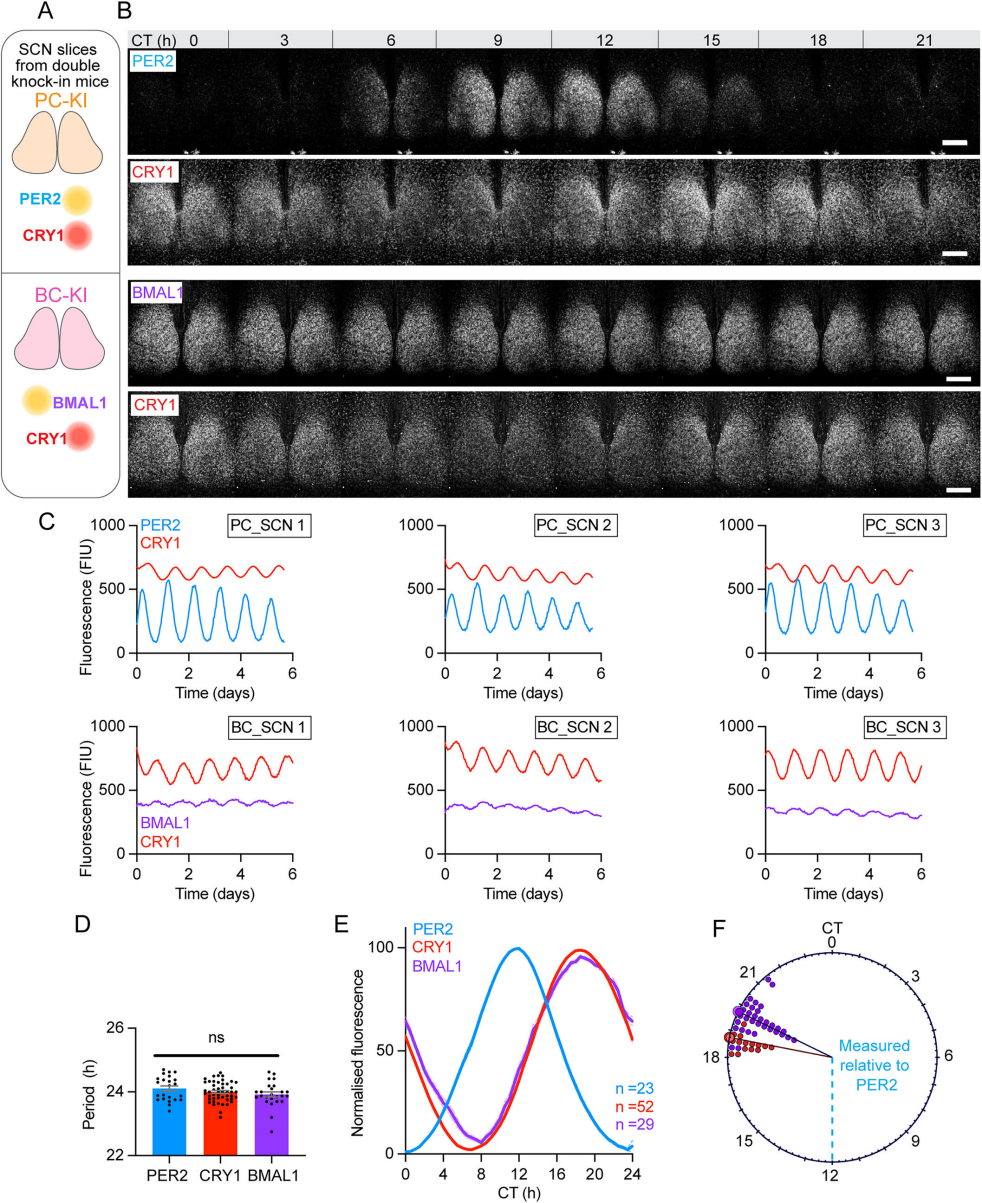
Dual-channel confocal timelapse imaging of endogenous circadian clock proteins reveals different SCN-wide circadian dynamics of PER2, CRY1 and BMAL1. (**A**) Schematic relating to images in (**B**). (**B**) Image montage shows dual-channel frames of confocal timelapse recordings of (upper) PER2 and CRY1 fluorescence in SCN from PC-KI mice and (lower) CRY1 and BMAL1 in SCN from BC-KI mice. Scale = 250 μm. (**C**) Representative fluorescence traces from dual-channel confocal recordings of SCN from (upper) PC-KI mice and (lower) BC-KI mice. (**D**) Group data (mean ± SEM) show circadian period of clock protein fluorescence oscillations (*n* > 24 SCN; One-way ANOVA with Tukey’s multiple comparison’s test: ns, PER2-CRY1: *P* = 0.4442, PER2-BMAL1: *P* = 0.1619, CRY1-BMAL1: *P* = 0.6401). (**E**) Average 24-h oscillation profiles, expressed as mean (unbroken lines) with SEM (dashed lines). (**F**) Rayleigh circular plot showing peak circadian phases of CRY1 (red) and BMAL1 (purple) oscillations, relative to PER2 (blue, CT12). Each dot represents 1 SCN. Source data are available online for this figure.

Having revealed the spectrum of intracellular properties, we examined the SCN-wide circadian dynamics of the clock proteins (Fig. 2A). All three cycled robustly across circadian time, albeit with different waveforms (Fig. 2B–D; Appendix Fig. S3). The pairs of clock proteins in each SCN exhibited the same period and CRY1 behaved comparably within PC-KI and BC-KI SCN slices (Appendix Fig. S3A–D). The timing of the peaks, however, were different between the three proteins (Fig. 2E,F). Whereas the peak phase of PER2, defined as circadian time (CT)12, marked the start of circadian night (Smyllie et al, 2016), the peak of CRY1 levels in the same SCN occurred in the late circadian night (~CT18.7), i.e., more than 6 h after PER2. The peak of BMAL1 (~CT20) was ~1 h later than CRY1 in the same SCN, and so by inference, ~8 h after PER2. Surprisingly, peak levels of repressor CRY1 and activator BMAL1 were closely aligned but peak levels of the two repressor proteins, PER2 and CRY1 were temporally segregated, suggestive of independent roles.

**Figure 3:**
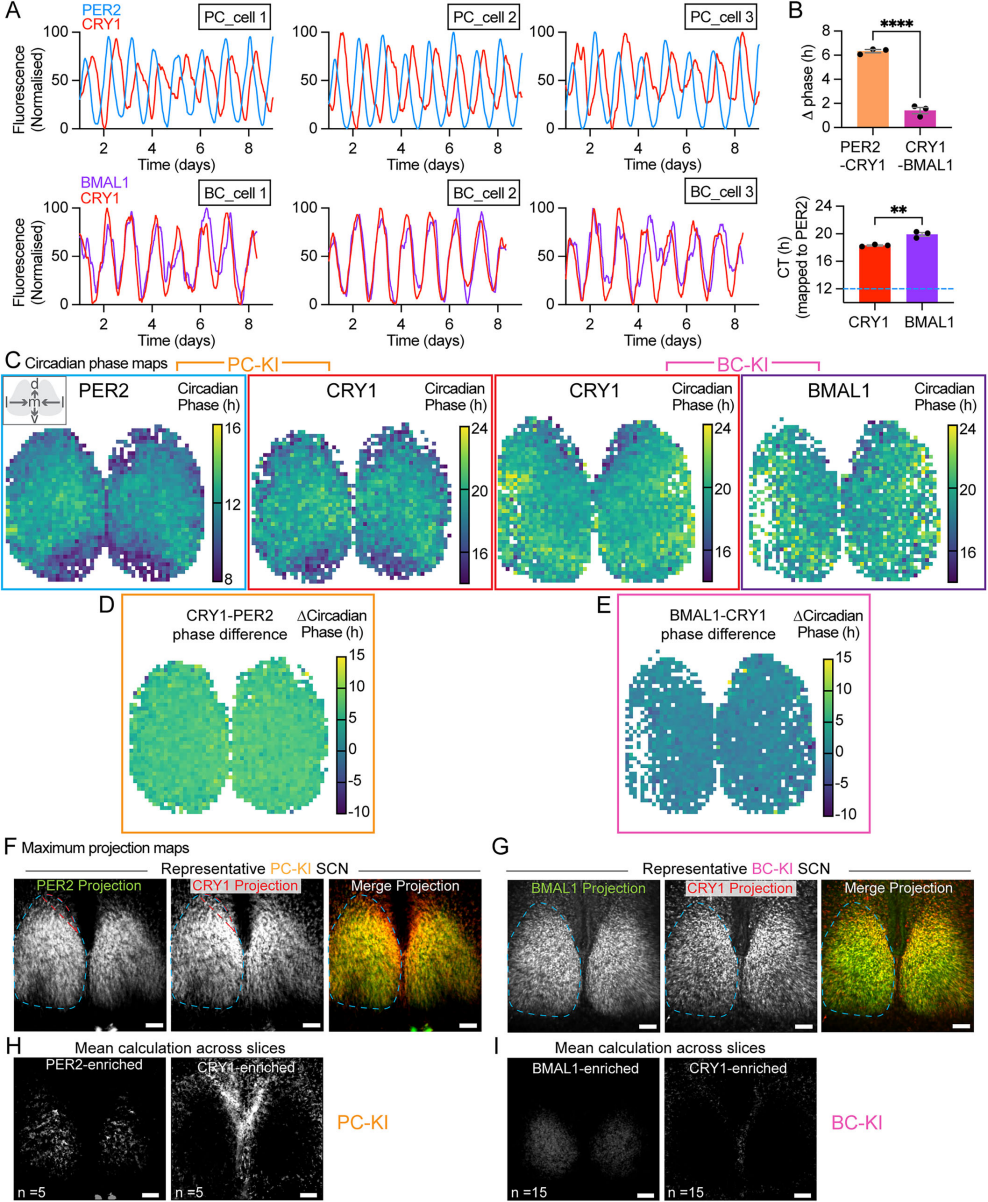
SCN clock proteins maintain fixed relationships within their spatiotemporal dynamics. (**A**) Representative fluorescence traces from individual cells within SCN slices from (upper) PC-KI mice and (lower) BC-KI mice (slices shown in **C**). (**B**) Group data showing (upper) peak phase differences between the pairs of protein oscillation within each cell (*n* = 3 cells per SCN; unpaired t-test; ****P < 0.0001) and (lower) peak phases of CRY1 and BMAL1 single cell oscillations using PER2 as a CT12 reference (*n* = 3 cells per SCN; unpaired t-test; **P = 0.0045; mean ± SEM shown), calculated from the single cells shown in (**A**). (**C**) Circadian phase maps for pairs of (left) PER2 and CRY1 oscillations in a representative PC-KI SCN and (right) CRY1 and BMAL1 oscillations in a representative BC-KI SCN. (Top left) The boxed schematic shows anatomical orientation labels d, m, v and l using the same key as in Fig. 1B,D. (**D**, **E**) Phase difference maps for the pairs of proteins shown in (**C**). (**F**) Maximum projection maps show total clock protein fluorescence across all image frames of confocal timelapse recordings of PER2 and CRY1, in the same representative PC-KI SCN shown in Fig. 2B. (**G**) As in (**F**), but for BMAL1 and CRY1, in the same representative BC-KI SCN shown in Fig. 2B. (**H**) Difference projection maps for (left) PER2 – CRY1 and (right) CRY1 – PER2 averaged across *n* = 5 PC-KI SCN. (**I**) Difference projection maps for (left) BMAL1 – CRY1 and (right) CRY1 – BMAL1 averaged across *n* = 15 BC-KI SCN. Source data are available online for this figure.

Protein oscillations within individual SCN cells were also robustly rhythmic across more than 8 days of recording and maintained comparable phase-relationships to those observed at the SCN-wide level, with CRY1 and BMAL1 delayed relative to PER2 by ~6.3 and ~7.5 h, respectively (Fig. 3A,B). The circadian activities of individual cells across the SCN are synchronous, they are not simultaneous, exhibiting a phase-wave spanning ~6 h between peak activity of the earliest and latest cells (Patton et al, 2020). To determine whether individual clock proteins follow the same spatiotemporal patterning, we created peak-phase maps for each, again making pairwise comparisons within the same SCN. Each protein displayed a similar, characteristic phase-distribution (Fig. 3C) with a phase-leading edge at the dorsal lip and a phase-lagging ventro-lateral region (Patton et al, 2020). The phase difference between the protein pairs was maintained across the SCN, i.e., the inter-protein phase differences within a cell were common to all cells, confirming that the temporal structure of the TTFL is conserved across the SCN network (Fig. 3D,E). Finally, we compared protein spatial expression patterns, independently of time, using maximum projection mapping of all images within the timelapse series (Fig. 3F,G). Remarkably, a region within the dorsomedial lip of the SCN contained CRY1-enriched cells that did not express appreciable levels of PER2 (Fig. 3H). Conversely, there was a modest enrichment of PER2 (i.e., lower CRY1) in the central SCN. Comparison of BMAL1 and CRY1, also showed a modest enrichment of BMAL1 in the central SCN (lower CRY1) but there was no specific enrichment of CRY1 in relation to BMAL1 in the dorsomedial lip (Fig. 3I). This suggests that the relative molecular composition of TTFL proteins may vary across SCN regional cell populations, especially the phase-leading dorso-medial margin.

### Quantifying relative protein abundance using a novel “colour-switch” PER2 mouse

**Figure 4:**
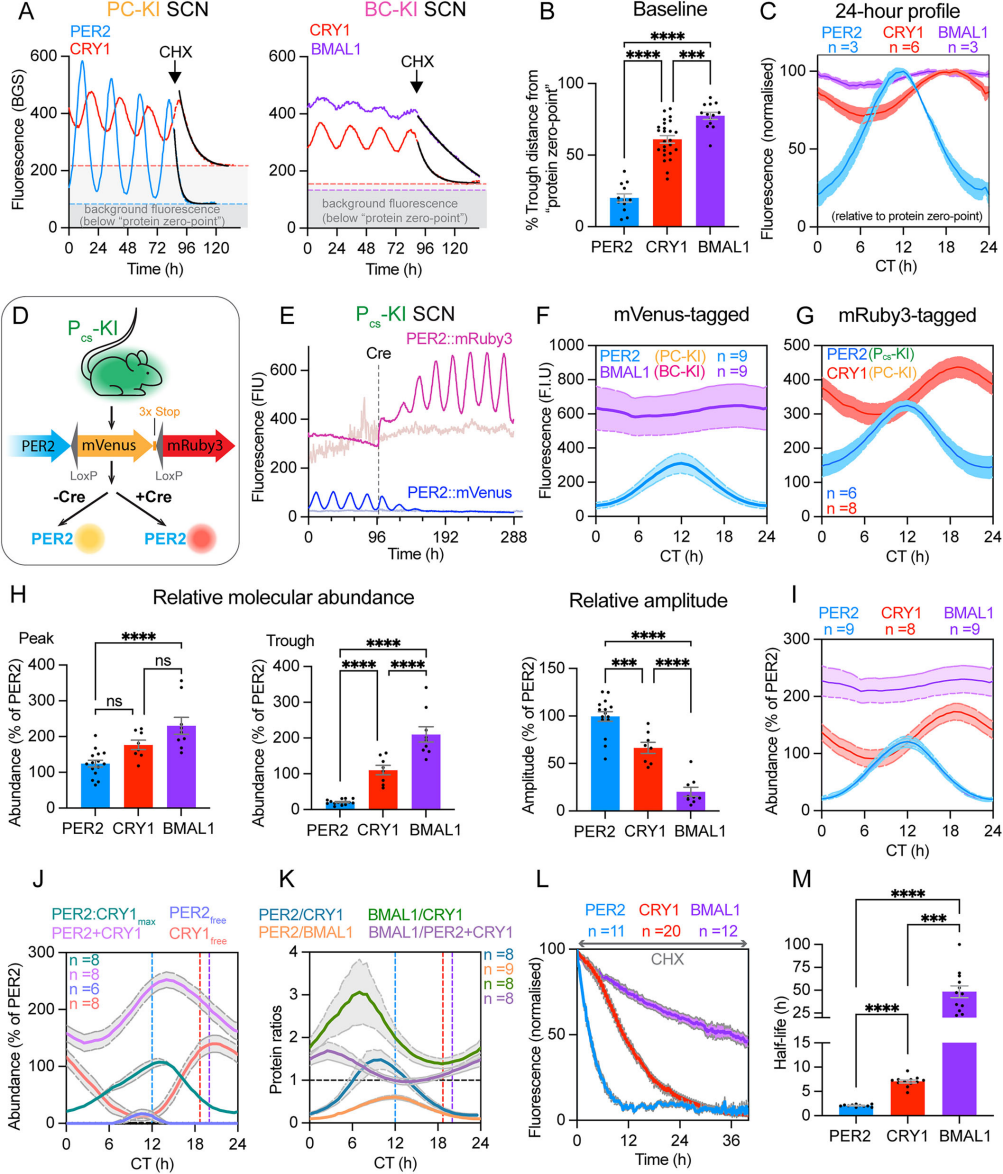
SCN clock protein stability correlates with circadian oscillation dynamics. (**A**) Fluorescence timelapse traces for (left) PER2 and CRY1 in a PC-KI SCN and (right) CRY1 and BMAL1 in a BC-KI SCN, where CHX treatment reveals the zero-point for each protein (coloured dotted line). The CHX-dependent decay is fit with a one-phase decay curve (black line). (**B**) Group data (*n* > 11 SCN per group) show oscillation baseline relative to protein zero-point (One-way ANOVA: ***P = 0.0003, ****P < 0.0001). (**C**) Average circadian profiles normalised to each clock protein zero-point (0%) and peak (100%). (**D**) Schematic shows design of PER2_colour-switch_ (P_cs_-KI) mouse. (**E**) Representative fluorescence trace showing *Ef1a*-Cre dependent switch from expression of PER2::Venus to PER2::mRuby3. Pale lines indicate background fluorescence. (**F**, **G**) Comparison of average circadian profiles for pairs of clock protein fusions that have the same fluorophore tag. (**F**) mVenus-tagged PER2 and BMAL1 recorded from PC-KI and BC-KI SCN, respectively. (**G**) mRuby3-tagged PER2 and CRY1 recorded from P_cs_-KI and PC-KI SCN, respectively. (**H**) Group data show relative abundance for (left) peak, (middle) trough, and (right) amplitude of clock protein oscillations relative to PER2, using P_cs_ to calibrate between fluorophores (*n* > 7 SCN; One-way AVOVA with Tukey’s multiple comparisons, for each dataset: ****P < 0.0001, ***P = 0.0006, ns, PER2-CRY1: *P* = 0.0537; CRY1-BMAL1: *P* = 0.0797). (**I**) Average circadian profiles show relative abundance of clock proteins through circadian time, relative to PER2 abundance (0% is PER2 zero-point, 20% is PER2 trough, 100% is PER2 peak). (**J**) Average circadian profiles (expressed relative to PER2) show inferred abundance of PER2 and CRY1 in different configurations: (purple) their total combined population; (teal) the maximum possible abundance of the PER2:CRY1 complexes; (red) free CRY1 and (blue) free PER2. These inferred configurations assume a 1:1 stoichiometry of PER2:CRY1 complexes, where excess molecules above this amount would be considered “free”. (**K**) Average circadian profiles show molecular ratios between clock proteins. (**L**) Group data show clock protein fluorescence decay dynamics during 40 μg/mL CHX treatment. (**M**) Group data show clock protein half-lives (*n* > 10 SCN per group; One-way ANOVA with Dunnett’s T3 multiple comparisons test: ***P = 0.0002, ****P < 0.0001). All group data in this figure are presented as mean ± SEM and analysed using One-way ANOVA with Tukey’s multiple comparison’s test unless specified otherwise. Source data are available online for this figure.

We next examined clock protein abundance in the SCN, relative to one another, across circadian time. We first defined a true “zero-point” by treating slices with cycloheximide (CHX) to deplete the specific fusion-protein fluorescence and thus quantify the non-specific background fluorescence (Figs. 4A and EV1A). This showed that the circadian trough of PER2 (i.e., the baseline of the oscillation) sits very close to its zero-point (~20% relative to its total range from zero-to-peak; Fig. 4B). In contrast, the CRY1 and BMAL1 troughs are significantly higher than PER2, and well above their own zero-points (~60% and ~75%, respectively). These differences become particularly clear when the average profile of each protein oscillation is plotted between its own peak (100%) and zero-point (0%) (Fig. 4C). The temporal dynamics of the three proteins therefore exhibit marked differences across circadian time, with the majority of PER2 being cleared every cycle, but CRY1, and even more so BMAL1, being present at significant levels at all phases of the cycle.

**Figure EV1:**

SCN relative molecular abundance of clock proteins measured using cre-recombinase dependent PER2 “Colour-Switch” mouse: P_cs_ KI. (**A**) Schematic illustrates how CHX-dependent decay curves account for the background level of fluorescence in order to determine the level of fluorescence where clock protein molecules = zero (zero-point). Once the true zero-point is known, relative amplitude and baseline can be calculated, relative to the total dynamic range of fluorescence. (**B**) Schematic showing the design of the PER2_cs_ KI, highlighting the locations for the PCR primers and their amplicons (upper horizontal bars; green: 5’ flanking amplicon and magenta: 3’ flanking amplicon) used for the initial identification of founder animals. An example Sanger sequence alignment (lower horizontal arrows; red) is shown for one founder animal (# 4 in PCR reactions). (**C**) 5’ (upper panel) and 3’ (lower panel) flanking PCRs identify pups, # 2, 3 and 4 as candidate knock in alleles. (**D**) Representative confocal images of an SCN slice (left, upper two rows) before transduction with *pEf1a*-Cre AAV (right, upper two rows) and 7 days after transduction, and (bottom) a merge of Venus and mRuby3 signals from before and after transduction, respectively. Scale bar = 200 μm. (**E**) Group data showing (upper) period, (lower) phase of PER2::Venus and PER2::mRuby3 oscillations from P_cs_-KI SCN (*n* = 6; period: paired t-test: ns, *P* = 0.1566; mean phase difference: 0.31 ± 0.25 h). All group data are presented as mean ± SEM.

Without a common fluorophore (PER2 and BMAL1 carried Venus but CRY1 carried mRuby3), this analysis was unable to quantify the relative molecular abundance of the three proteins. We therefore expanded our approach with a new PER2 “colour-switch” KI (P_cs_-KI) mouse, based on the dual-colour luciferase mouse (Shan et al, 2020). For the P_cs_-KI, endogenous PER2 again carries a C-terminal Venus fusion, as in the PC-KI mice. In the presence of Cre-recombinase (Cre), however, the *Venus* sequence is excised and an mRuby3 C-terminal fusion to PER2 is expressed in its place (Figs. 4D and EV1B,C). SCN from P_cs_-KI mice showed very stable circadian oscillations of PER2::Venus fluorescence (Fig. 4E; Appendix Fig. S4A) comparable to those of the existing PC-KI (Appendix Fig. S4B,C), confirming that the KI did not alter the SCN TTFL nor the fidelity of the Venus signal. Following transduction with an AAV encoding the pan-cellular *pEF1a-*Cre, the Venus signal disappeared over 4–6 days and was replaced by a high-amplitude, circadian oscillation of mRuby3 fluorescence (Fig. 4E; Appendix Fig. S4A) in spatial and temporal register to the previous Venus signal (Fig. EV1D). Within the same SCN, the properties of the PER2 oscillation, including the period and phase, were comparable between the two fluorescent reporters (Fig. EV1E), reiterating that TTFL function was not affected by the different PER2 tags.

Because the fusion did not affect the properties of the fluorescent proteins, the P_cs_-KI allowed us to calibrate, on an equimolar basis for PER2, the intensity of the mRuby3 signal against that of the Venus. We could then compare the relative molecular abundance of all three proteins: BMAL1 with PER2 (Venus reporter), CRY1 with PER2 (mRuby3 reporter) and, by extension, CRY1 with BMAL1 (Fig. 4F–I). In terms of molecular abundance, the elevated circadian baselines of the BMAL1 and CRY1 oscillations were again evident (Fig. 4F,G). Importantly, the abundance of BMAL1 was always appreciably greater than PER2 (>2-fold at the PER2 peak), whereas CRY1 peak abundance was only slightly higher than PER2 at the PER2 peak (Fig. 4H). In an echo of the earlier CHX-dependent baseline calculations, it was at the rhythm nadirs where the greatest differences of molecular abundance were observed. The trough of CRY1 abundance sat ~5 times higher than the trough of PER2, and the trough for BMAL1 was ~10 times higher (Fig. 4H). By contrast, the oscillation amplitudes had the opposite relationship. The amplitude of the CRY1 oscillation, in terms of molecular abundance, was only ~65% of PER2, and BMAL1 had an even smaller oscillation of ~20% of PER2 (Fig. 4H).

Quantification of relative molecular abundances across the circadian cycle (Fig. 4I) confirmed that the level of PER2 is likely the most limiting in the formation of PER2:CRY1, where these heterodimers exist within larger protein assemblies (Aryal et al, 2017). In contrast, CRY1 and BMAL1 are available throughout the circadian cycle, creating an evolving landscape of protein stoichiometries though circadian time. This allowed us to infer the likely dynamics of the incorporation of PER2 and CRY1 into heteromeric protein complexes (Fig. 4J), by calculating their theoretical maximum relative abundance (PER2:CRY1_max_). This maximum assumes 100% preference for heterodimer formation when a partner is available, leaving any excess PER2 or CRY1 as “free” (PER2_free_ and CRY1_free_). As an aggregate single population, the combined molecular abundance of PER2 and CRY1 (PER2 + CRY1) oscillated with a peak at ~CT14 as did PER2:CRY1_max_, both driven by the total abundance of PER2 (PER2_total_; Fig. 4I,J). Importantly, CRY1_free_ oscillated with a peak-phase (~CT21) that was ~7 h after PER2:CRY1_max_ and ~3 h after CRY1_total_. By contrast, PER2_free_ had a minimal presence of ~10% of PER2_total_ between CT8-13. We then quantified the stoichiometric ratios of the three proteins across the circadian cycle (Fig. 4K). The ratio of PER2/CRY1 was ~6:5 at the peak of PER2 abundance (CT12) dropping to ~1:5 at its trough (CT0). A similar trend was observed for PER2/BMAL1, where a ratio of ~2:3 was observed at CT12, dropping to ~1:10 at CT0. The BMAL1/CRY1 ratio oscillated between ~4:3 and ~3:1. Strikingly, the PER2 + CRY1 combined population of potential repressor proteins, was broadly comparable to the abundance of the transcriptional activator, BMAL1, such that the ratio of repressors to activator sat just above 1:1 at all circadian phases. These relative molecular abundance measures provide new insight into how the availability of clock proteins to both interact and act independently evolves across the circadian time. More specifically, they indicate that temporally complex changes in the relative abundance of PER2 and CRY1 will drive the central pivot of the TTFL, i.e., BMAL1/E-box-mediated trans-activation.

### Stability of endogenous PER2, CRY1 and BMAL1 in the SCN

The striking contrasts in the waveform of the protein oscillations, especially baseline, and amplitude, led us to hypothesise that they could arise from differential protein stability. To test this, we compared the half-lives of the three proteins as determined from paired CHX fluorescence decay curves (Fig. 4L,M). The responses of CRY1 to CHX were not significantly different between SCN treated at either CT15 and CT19 and so data were combined (Appendix Fig. S5A–C). PER2 and CRY1 showed conventional mono-exponential decays, with both proteins depleted after several hours of CHX. In contrast, BMAL1 declined progressively but very slowly and did not fully deplete over 72 h of treatment. The half-life of BMAL1 was therefore estimated using a projected mono-exponential curve-fit, which likely represents an underestimate. Of the three proteins, PER2 had the shortest half-life (~2 h), CRY1 was significantly longer (~7 h) whereas BMAL1 had significantly the longest, in the order of several days (~48 h). Their half-lives did not vary appreciably across the sub-regions of the SCN (Appendix Fig. S5D). These protein-specific stabilities therefore correlate with the level of signal at the trough of the respective oscillations: least stable PER2 was cleared to a low nadir, BMAL1 had the longest half-life, the highest trough and smallest amplitude, whereas CRY1 was intermediate for these properties.

### Effects of PER protein stabilisation on SCN TTFL function

**Figure 5:**
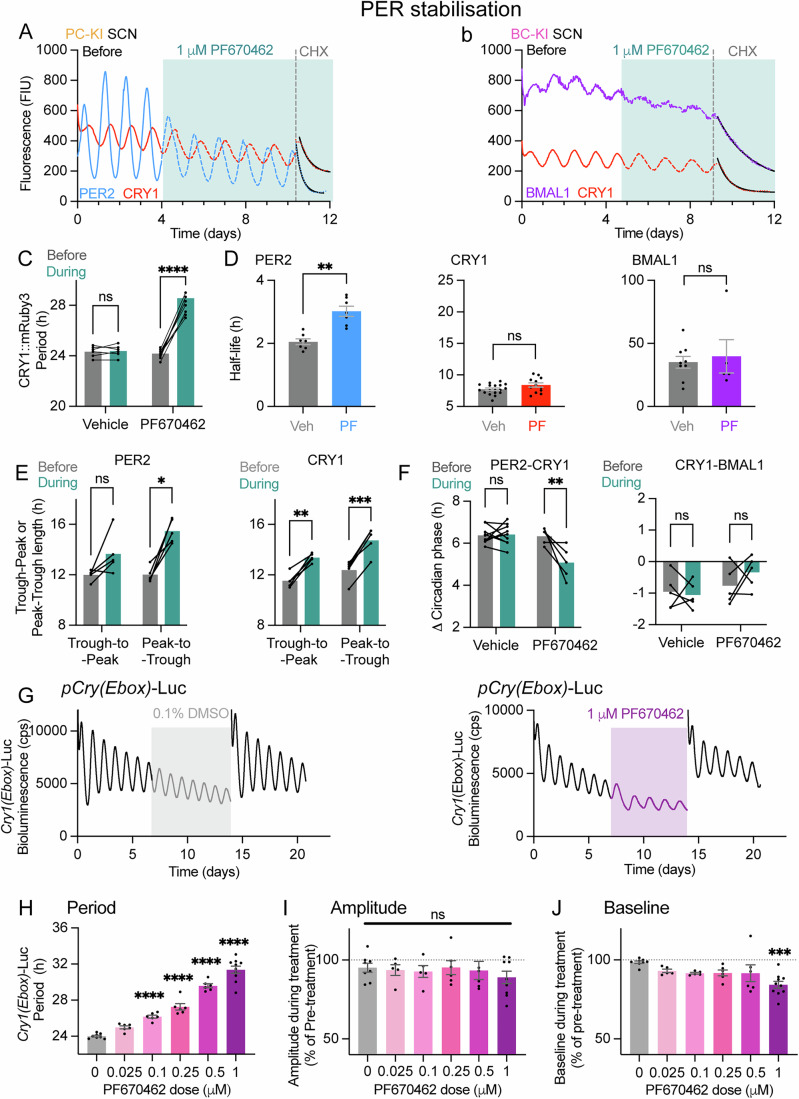
Pharmacological stabilisation of PER lengthens circadian period without perturbation of other protein half-lives or TTFL waveform. (**A**, **B**) Fluorescence traces of (**A**) PER2 (blue) and CRY1 (red) in a representative PC-KI SCN or (**B**) BMAL1 (purple) and CRY1 (red) oscillations in a representative BC-KI SCN recorded before and during (teal shading) treatment with PER stabiliser drug, PF670462 (1 μM), followed by treatment with 40 μg/mL CHX (at grey dotted line). CHX-dependent fluorescence decay was fit with a one-phase decay curve (black curves). (**C**–**F**) Group data show the effect of PF670462 treatment on (**C**) the circadian period of CRY1 fluorescence oscillations (*n* > 5 SCN per group; ns, *P* = 0.9859, ****P < 0.0001), (**D**) half-lives of (left) PER2, (middle) CRY1 and (right) BMAL1 (*n* > 4 SCN per group; mixed effects repeated measures model with Šídák’s multiple comparisons test: PER2: *P = 0.0013, CRY1: ns, *P* = 0.2878, BMAL1: ns, *P* = 0.9851) (**E**) oscillation waveforms (*n* > 4 SCN per group) for (left) PER2 (*P* = 0.138, *P = 0.0145) and (right) CRY1 (**P = 0.0024, ***P = 0.0009) and (**F**) the difference (Δ) between (left) PER2 and CRY1 oscillation peak phases (ns, vehicle: *P* = 0.9770; PF670462: **P = 0.0026) and (right) CRY1 and BMAL1 peak phase (ns, vehicle: *P* = 0.9527; PF670462: *P* = 0.4533) before (grey) or during (teal) treatment with 1 μM PF670462. (**G**) Representative *Cry1(Ebox)*-Luc bioluminescence traces show the effect on either (left) vehicle or (right) 1 μM PF670462 treatment (grey/purple shaded area with grey/purple data trace). Pre-treatment and wash-out of treatment shown as black data traces. (**H**–**J**) Group data (*n* > 5 SCN per group) show dose-dependent effects of PF670462 treatment on (**H**) circadian period (ns, *P* = 0.1041, ****P < 0.0001), (**I**) amplitude (ns, *P* = 0.9994, *P* = 0.9950, *P*  > 0.9999, *P* = 0.9985) and (**J**) baseline (ns, *P* = 0.4662, *P* = 0.247, *P* = 0.2261, *P* = 0.2046, ***P < 0.0002). All group data are presented as mean ± SEM. (**C**–**F**) were analysed using a Two-way ANOVA with Šídák’s multiple comparisons test, unless otherwise specified. (**H**–**J**) were analysed using a One-way ANOVA with Dunnett’s multiple comparison’s test, where *P* values for multiple comparisons with 0 μM are listed in order of concentration lowest to highest. Source data are available online for this figure.

Having revealed the contrasting properties of the negative regulators PER2 and CRY1, we wished to define their respective influence on the SCN clock by manipulating their stability and examining TTFL function. To target PER proteins, we used PF670462, which stabilises PER1 and PER2 by interfering with their phosphorylation by casein kinase 1 (CK1)*δ* (Meng et al, 2010). To quantify the effect of PF670462 on protein stability, we recorded PER2, CRY1 and BMAL1 fluorescence rhythms in double KI SCN slices treated with vehicle or 1 μM PF670462 and then with CHX (Fig. 5A,B). This showed, first, that treatment with PF670462 (1 μM) caused a significant lengthening of circadian period, from ~24 to ~28 h, as reported by CRY1::mRuby3 (Fig. 5C). Second, period-lengthening was associated with increased half-life of PER2, from ~2 h to ~3 h (Fig. 5D). This stabilising effect was specific, insofar as it was not seen for CRY1 and BMAL1, neither of which were affected by PF670462 and thus their stability was independent of CK1*δ* and the stability of PER proteins.

PF670462 prolonged the interval between the peak and trough (descending phase) of endogenous PER2 by ~3.5 h, whereas the trough-to-peak duration (rising phase) was not affected (Fig. 5E). This phase-specific effect explained the bulk of the period-lengthening. In contrast, CRY1 showed an approximately symmetrical extension of both the rising phase (~1.8 h) and the falling phase (~2.3 h) (Fig. 5E). Consequently, the phase difference between the peaks of PER2 and CRY1 was reduced from ~6.5 h to ~5 h, whereas the difference between CRY1 and BMAL1 was unaltered at ~1 h (Fig. 5F). This suggests that the peak of PER2 extended later, independently of the other two proteins, and again argues for independence of the stability CRY1 and BMAL1 from that of PER2 protein.

To gain insight into the potential mechanism of action of PER protein within the TTFL, we treated SCN slices of mice carrying bioluminescent reporters of E-box-dependent transcription (*Cry1(Ebox)-*Luciferase; *Cry1*(*Ebox)*-Luc) (Maywood et al, 2013) or PER2 post-translational protein expression (PER2::Luciferase [PER2::Luc]) (Yoo et al, 2004) with PF670462 (Figs. 5G–J and EV2). PF670462 dose-dependently and reversibly lengthened SCN period as reported by either reporter. PF670462 did not, however, affect the amplitude of *Cry1(Ebox)*-Luc (Fig. 5I,J) or PER2::Luc (Fig. EV2D) oscillations, although at the highest dose there was a minor suppression of baseline for both. Thus, selective stabilisation of PERs altered the waveform of PER2 in a phase-specific manner whereas CRY1 waveform was elongated across the whole TTFL cycle. Furthermore, the stability of PER proteins has a significant influence over the period of the SCN TTFL, but through a route that is independent of alterations in the stability of CRY1 and BMAL1 proteins. Finally, these results highlight relatively weak inhibition of E-box transcription by stabilised PER proteins (Langmesser et al, 2008).

**Figure EV2:**

The effect of stabilisation of PER on TTFL rhythms. (**A**, **B**) Representative bioluminescence traces show TTFL reporters *Cry1(Ebox)*-Luc (transcriptional) and PER2::Luc (translational) in SCN slices treated with different doses of PER stabiliser drug, PF670462. Before treatment and wash-out after treatment traces are shown in black. Treatment traces are shown in pink for *Cry1(Ebox)*-Luc and blue for PER2::Luc SCN treated with PF670462. Vehicle traces are shown in grey for both reporters. (**C**) Circadian period of (upper) *Cry1*(*Ebox*)-Luc or (lower) PER2::Luc oscillations before (pre), during and after wash-out (post) of different concentrations PF670462. This shows dose dependence and reversibility of effect on circadian period. (**D**) PF670462 dose dependently (upper) lengthens period (ns, *P* = 0.4042, ***P = 0.0002, ****P < 0.0001) and (lower) minimally drops circadian baseline (ns, *P* = 0.3470, *P = 0.0108, **P = 0.0052, ***P = 0.0001) but does not alter circadian amplitude (ns, *P* = 0.9478, *P* = 0.3996, *P* = 0.8470, *P* = 0.8915, *P* = 0.0747) of PER2::Luc oscillations. All group data are presented as mean ± SEM and data in (**D**) (*n* > 5 SCN per dose group) are analysed with One-way ANOVAs with Dunnett’s multiple comparisons tests.

### Effects of CRY1 protein stabilisation on SCN TTFL function

**Figure 6:**
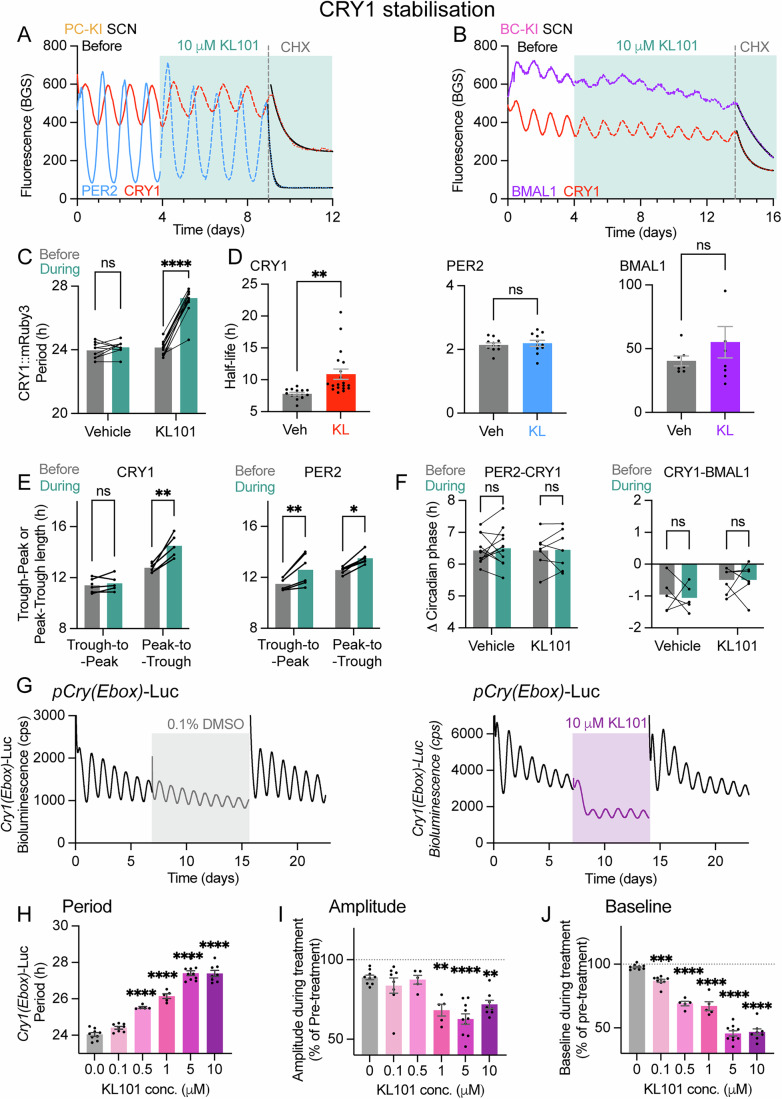
Pharmacological stabilisation of CRY1 lengthens circadian period and suppresses TTFL transcription without perturbation of other protein half-lives or TTFL waveform. (**A**, **B**) Fluorescence traces of (**A**) PER2 (blue) and CRY1 (red) in a representative PC-KI SCN or (**B**) BMAL1 (purple) and CRY1 (red) oscillations in a representative BC-KI SCN recorded before and during (teal shading) treatment with CRY1 stabiliser drug, KL101 (10 μM), followed by treatment with 40 μg/mL CHX (at grey dotted line). CHX-dependent fluorescence decay was fit with a one-phase decay curve (black curves). (**C**–**F**) Group data show the effect of KL101 treatment on the (**C**) Circadian period of CRY1 fluorescence oscillations (*n* > 8 SCN per group; ns, *P* = 0.6726, ****P < 0.0001). (**D**) Half-lives of (left) CRY1, (middle) PER2 and (right) BMAL1 (*n* > 4 SCN per group; mixed effects repeated measures model with Šídák’s multiple comparisons test: CRY1: **P = 0.0040, PER2: ns, *P* = 0.9612, BMAL1: ns, *P* = 0.6390) (**E**) oscillation waveforms (*n* > 4 SCN per group) for (left) CRY1 (ns, *P* = 0.8390, **P = 0.0094) and (right) PER2 (*P = 0.0121, **P = 0.0060) and (**F**) the difference (Δ) between (left) PER2 and CRY1 peak phase (*n* > 6 SCN per group; ns, vehicle: *P* = 0.8462; KL101: *P* = 0.9932), and (right) CRY1 and BMAL1 peak phase (*n* > 4 SCN per group; ns, vehicle: *P* = 0.9349; KL101: *P* = 0.9999). (**G**) Representative *Cry1(Ebox)*-Luc bioluminescence traces show the effect on either (left) vehicle, or (right) 10 μM KL101 treatment (grey/purple shaded area with grey/purple data trace). Pre-treatment and wash-out of treatment shown as black data traces. (**H**–**J**) Group data (*n* > 4 SCN per group) show dose-dependent effects of KL101 treatment on (**H**) circadian period (ns, *P* = 0.1294, ****P < 0.0001), (**I**) amplitude (ns, *P* = 0.6866, *P* = 09989 **P = 0.0011, ****P < 0.0001, *P* = 0.0020) and (**J**) baseline (***P = 0.0005, ****P < 0.0001). Group data are presented as mean ± SEM. (**C**–**F**) were analysed using a Two-way ANOVA with Šídák’s multiple comparisons test, unless otherwise specified. (**H**–**J**) were analysed using a One-way ANOVA with Dunnett’s multiple comparison’s test, where *P* values for multiple comparisons with 0 μM are listed in order of concentration lowest to highest. Source data are available online for this figure.

We next asked how stabilisation of CRY1 protein affected the SCN TTFL. The small molecule KL101 selectively stabilises CRY1 protein by disrupting its interactions with FBXL ubiquitin-ligases (Miller et al, 2020). We recorded PER2, CRY1 and BMAL1 fluorescence rhythms in double KI SCN slices treated with vehicle or KL101 and followed by CHX (Fig. 6A,B). Treatment with 10 μM KL101 lengthened SCN period, as reported by CRY1::mRuby3, by ~3 h to ~27 h (Fig. 6C). This was associated with a significant increase in the half-life of CRY1 from ~8 h to ~11 h (Fig. 6D). The stability of neither PER2 nor BMAL1 was affected by KL101, confirming their independence of CRY1 stability.

KL101 prolonged the descending phase of the CRY1 oscillation waveform by ~2 h, whereas the duration of the rising phase was not significantly affected (Fig. 6E). The phase-specific effect of KL101 therefore explained the bulk of the period-lengthening of the CRY1 oscillation. By contrast, the rhythm of PER2 showed a symmetrical extension of both the rising and descending phases (Fig. 6E). The lengthening of period did not, however, affect the internal phasing of the TTFL: the phase differences between peaks of CRY1 and either PER2 or BMAL1 were unaltered (Fig. 6F). Therefore, the marked temporal segregation of the abundance of PER2 and CRY1 persisted in the presence of stabilised CRY1.

**Figure EV3:**
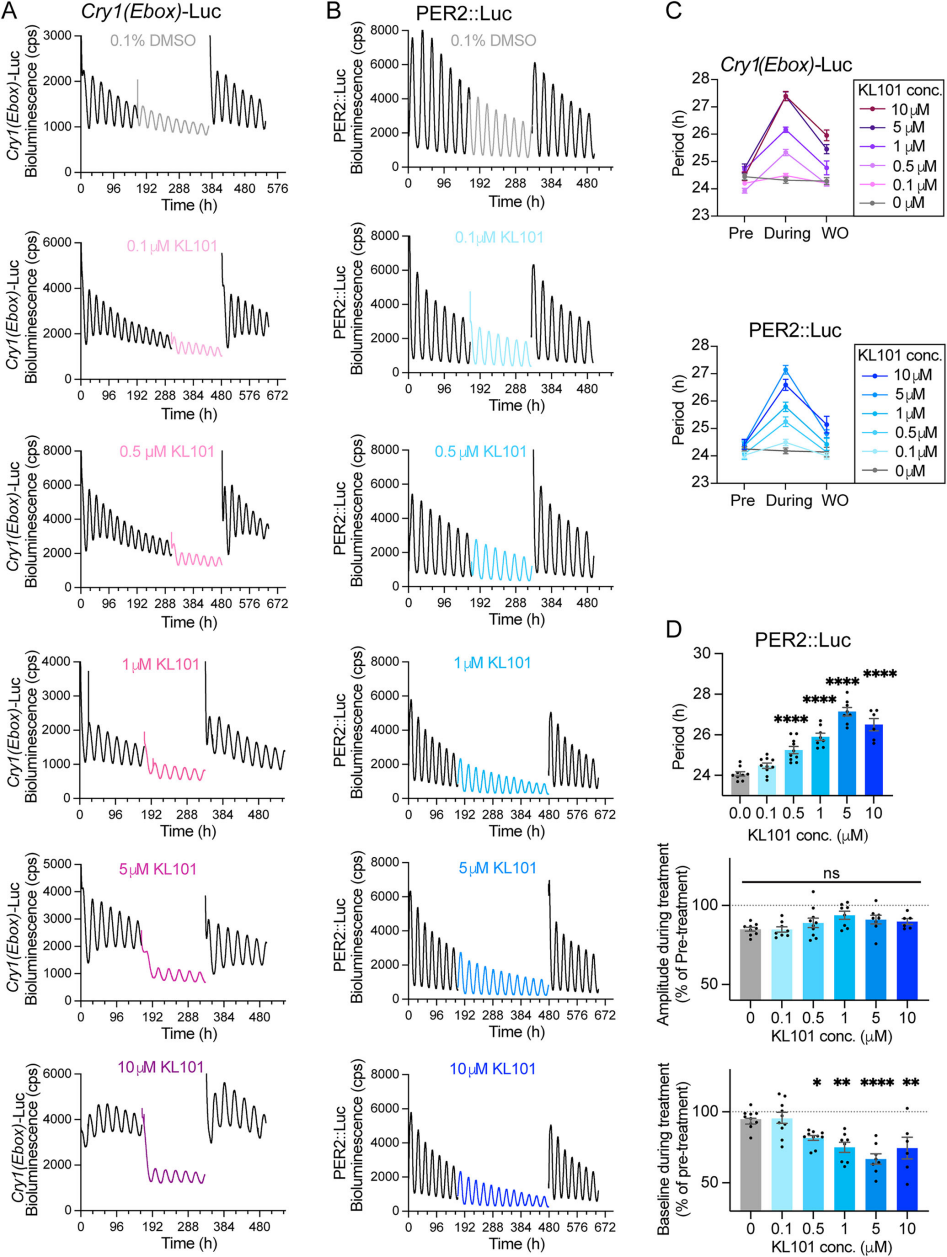
The effect of stabilisation of CRY1 on TTFL rhythms. (**A**, **B**) Representative bioluminescence traces show TTFL reporters *Cry1(Ebox)*-Luc (transcriptional) and PER2::Luc (translational) in SCN slices treated with different doses of CRY1 stabiliser drug, KL101. Before treatment and wash-out after treatment traces are shown in black. Treatment traces are shown in pink for *Cry1(Ebox)*-Luc and blue for PER2::Luc SCN treated with KL101. Vehicle traces are shown in grey for both reporters. (**C**) Circadian period of (left) *Cry1*(*Ebox*)-Luc or (right) PER2::Luc oscillations before (pre), during and after wash-out (post) of different concentrations KL101 shows dose dependence and reversibility of effect on circadian period. (**D**) KL101 dose dependently (upper) lengthens period (ns, *P* = 0.3274, ****P < 0.0001), does not alter circadian amplitude (ns, *P* > 0.9999, *P* = 0.5658, *P* = 0.2398, *P* = 0.5226) but does (lower) drops circadian baseline (*P* > 0.9999, *P = 0.0345, **P < 0.0028, ****P < 0.0001) of PER2::Luc oscillations. All group data are presented as mean ± SEM and data in (**D**) (*n* > 5 SCN per group) are analysed with One-way ANOVAs with Dunnett’s multiple comparisons tests.

To explore CRY1-sensitive targets in the TTFL, we tested the effect of CRY1 stabilisation on *Cry1(Ebox)*-Luc or PER2::Luc bioluminescent reporters in SCN slices (Figs. 6G and EV3). KL101 dose-dependently and reversibly lengthened the period of SCN carrying either reporter (Figs. 6H and EV3B,C). At the maximum dose (10 μM KL101), period was again lengthened by ~3 h. Stabilisation of CRY1 had potent effects on the oscillation of *Cry1(Ebox)*-Luc. In comparison with vehicle treatment, the amplitude of the *Cry1(Ebox)*-Luc reporter was dose-dependently suppressed by KL101 (Fig. 6I), whereas the PER2::Luc oscillation was not affected (Fig. EV3D). For both reporters, however, stabilisation of CRY1 caused a significant, dose-dependent suppression of the oscillation baseline (Fig. 6J) and EV 3D). These results indicate strong inhibition of E-box-mediated transcription by stabilised CRY1.

### The effects of PER and CRY stabilisation on the TTFL are additive

**Figure 7:**
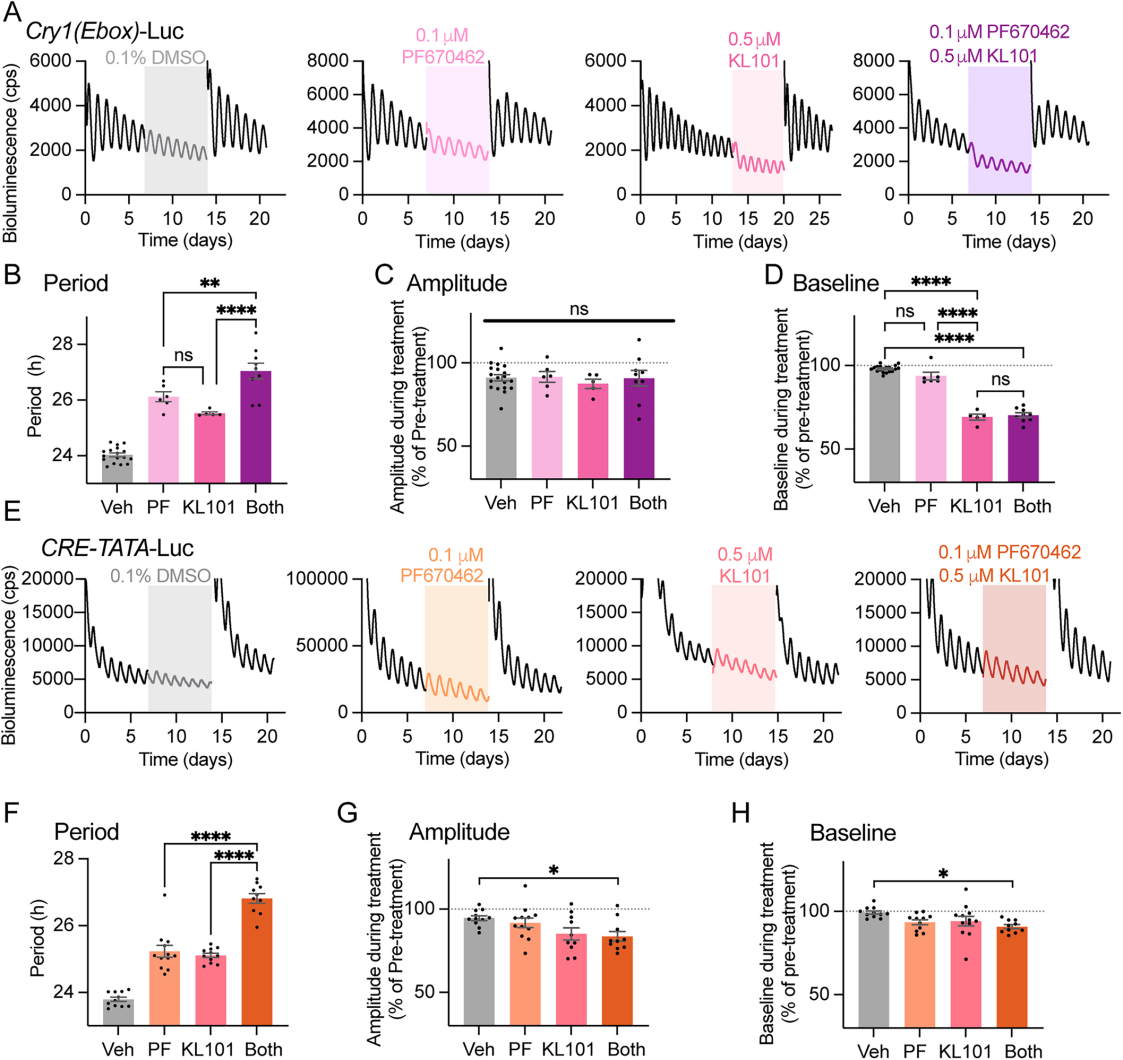
The effects of combined stabilisation of PER and CRY1 on circadian oscillations is additive. (**A**) Representative *Cry1(Ebox)*-Luc bioluminescence traces show the effect on either (grey, far left) vehicle, (light pink, mid left) 0.1 μM PF670462, (bright pink, mid right) 0.5 μM KL101 or (purple, far right) both drugs together (at the same concentration as applied individually). Pre-treatment and wash-out of treatment shown as black data traces and treatment duration indicated with grey/pink/purple shading). (**B**–**D**) Group data show the effect of applying both PF670462 and KL101 on *Cry1(Ebox)*-Luc oscillation properties (*n* > 5 SCN per group) on (**B**) circadian period (ns, *P* = 0.2174, **P = 0.0068, ****P < 0.0001), (**C**) amplitude (ns, *P* = 0.8866) and (**D**) baseline (ns, *P* = 0.0554, ****P < 0.0001, *P* = 0.9312). (**E**) As in a, above but for *CRE-TATA*-Luc reporter bioluminescence: (grey, far left) vehicle, (light orange, mid left) 0.1 μM PF670462, (salmon pink, mid right) 0.5 μM KL101 or (dark orange, far right) both drugs together (at the same concentration as individually applied). (**F**–**H**) Group data show the effect of applying both PF670462 and KL101 on *CRE-TATA*-Luc oscillation properties (*n* > 9 SCN per group) on (**F**) circadian period (ns, *P* = 0.8982, ****P < 0.0001), (**G**) amplitude (*P* = 0.8832, *P* = 0.1041, *P = 0.0458, *P* = 0.3459, *P* = 0.1836, *P* = 0.9835) and (**H**) Baseline (*P* = 0.1426, *P* = 0.2260, *P = 0.0192, *P* = 0.9909, *P* = 0.7887, *P* = 06072). Group data are presented as mean ± SEM and analysed using a One-way ANOVA with Tukey’s multiple comparison’s test. For (F–**H**), all unlabelled comparisons were not significant. In the legend, these ns *P*-values are listed in bar chart order (vehicle - PF670462 - KL101 - both). Source data are available online for this figure.

Although stabilisation of either PER or CRY1 lengthened circadian period, their effects on the SCN TTFL were markedly different. The stabilisation of PER, but not stabilisation of CRY1, altered intra-TTFL phasing, whereas the stabilisation of CRY1 but not of PER was far more potent in suppressing the E-box-driven transcriptional arm of the TTFL. This indicates that the effects of PER and CRY1 stability within the SCN TTFL are likely independent of each other. To explore this further, we tested for potential additive effects by combined application of both PF670462 and KL101 at doses that were in the middle of their respective dose–response ranges (PF670462: 0.1 μM; KL101: 0.5 μM). As anticipated, the application of either drug alone to SCN carrying the *Cry1(Ebox)*-Luc reporter lengthened period significantly (Fig. 7A). Dual application lengthened period significantly further than did either drug alone (Fig. 7B), thereby confirming additive, independent effects of increased PER and CRY1 stability. At these intermediate doses, there was no effect on rhythm amplitude for either drug alone or on application of both drugs (Fig. 7C). As described earlier, 0.5 μM KL101 treatment lowered *Cry1(Ebox)*-Luc baseline, but 0.1 μM PF670462 did not. On application of both drugs, the baseline dropped to a level that matched KL101 treatment alone, again emphasising negative transcriptional feedback by CRY1 but no additional action by stabilised PER (Fig. 7D).

**Figure EV4:**
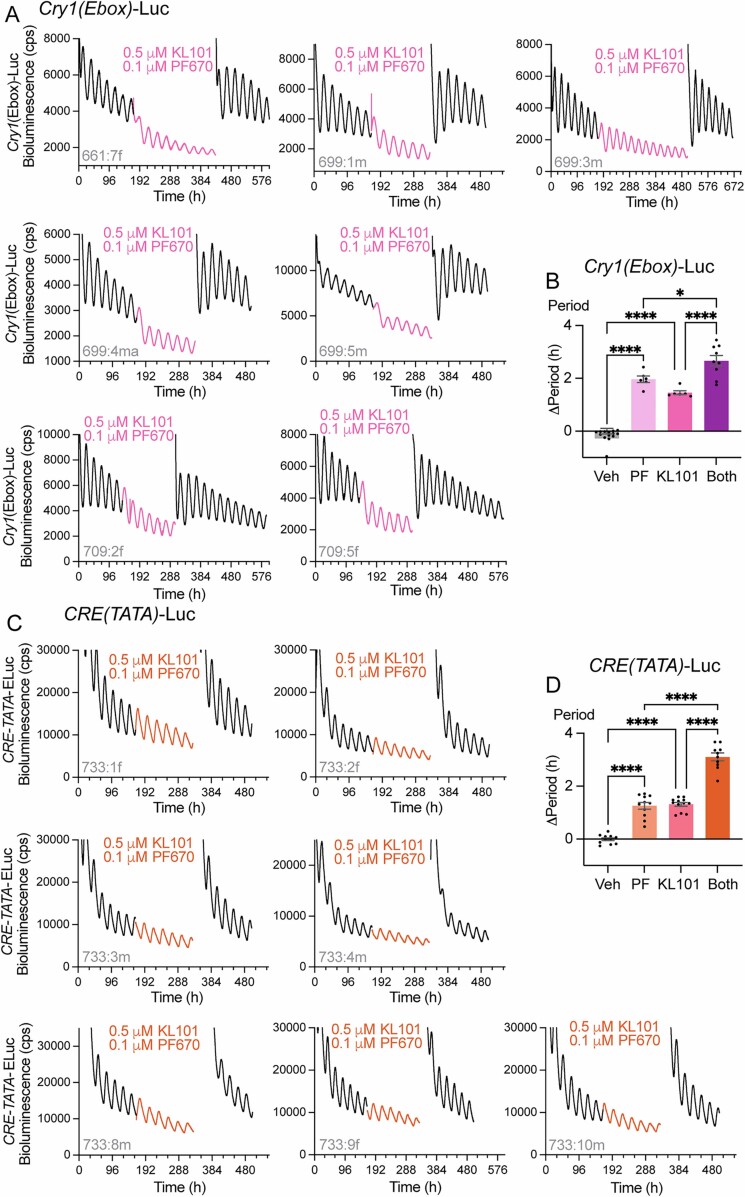
Combined stabilisation of PER and of CRY1 have an additive effect on the SCN circadian clock. (**A**) Representative bioluminescence recordings of *Cry1*(*Ebox*)-Luc intra-TTFL transcriptional oscillations in SCN slices before (black trace) and during double-drug-treated with 0.1 μM PF670462 and 0.5 μM KL101 (pink trace) and after wash-out (2nd black trace). (**B**) Group data show circadian period change (Δ) of *Cry1*(*Ebox*)-Luc oscillations, after drug treatment compared to pre-treatment, where there was an additive effect of double-drug treatment compared with single drug treatment (*P = 0.0190, ****P < 0.0001). (**C**) As in (**A**), but for *CRE*-*TATA*-Luc extra-TTFL transcriptional oscillations (treatment trace in dark orange). (**D**) as in (**B**), but for *CRE*-*TATA*-Luc oscillations. (****P < 0.0001). All group data (*n* > 5 SCN per group) are presented as mean ± SEM and are analysed with One-way ANOVAs with Tukey’s multiple comparisons tests.

To examine SCN circadian function beyond the core TTFL, using a reporter independent of direct regulation by PER and CRY proteins, we transduced wild-type, i.e., reporter-free, SCN with an AAV encoding a Ca^2+^/cAMP Response Element (*CRE*)-Luc reporter (*CRE*-*TATA*-Luc) (Figs. 7E–H and EV4). *CRE*-driven bioluminescence was highly circadian with a distinctive saw-tooth waveform (Brancaccio et al, 2013), rather than the sinusoidal waveform of TTFL reporters. Treatment with either PF670462 or KL101 lengthened period and dual application lengthened it further in an additive manner (Fig. 7F), confirming that *CRE*-dependent transcription is downstream of, and regulated by, the PER- and CRY1-sensitive TTFL. In contrast to *Cry1(Ebox)*-Luc transcription, the effects of the drugs alone and in combination on the amplitude and baseline of the *CRE*-*TATA*-Luc oscillations were minimal, with only combined treatment causing minor suppression of ~10% for both measures (Fig. 7G,H). Taken together, specific pharmacological perturbations of PER and CRY1 stability reveal independent control of their stabilities that have additive effects on the TTFL, that are consistent with the differing temporal profiles of (early) PER2 and (late) CRY1 abundance.

## Discussion

By applying real-time, combinatorial fluorescence imaging, we quantified the dynamic behaviours of endogenous PER2, CRY1 and BMAL1 within SCN slices. Their intracellular and SCN-wide properties varied widely, falling consistently on a spectrum: PER2 the most dynamic, CRY1 intermediate and BMAL1 the most static. We show that the circadian cycles of PER2 and CRY1 are temporally segregated, and by using a novel PER2 colour-switch mouse we determined the relative molecular abundance of all three proteins, mapping their circadian changes and thereby defining the limits for interaction and individual function. The dynamic nature of these clock proteins strongly suggests that circadian regulation at E-boxes is not a static and strict stoichiometric phenomenon but, instead, should be considered as a dynamic hub of transiently associating and dissociating transcription factors. By selectively stabilising either PER or CRY1, we show that their turnover determines overall TTFL dynamics and that CRY1 is the more potent repressor of E-box-mediated circadian transcription. Finally, we show that PER and CRY have different and independent, additive actions on the TTFL. Inevitably, PER1 and CRY2 proteins, which we did not image here, will also contribute to TTFL repressor complexes, but nevertheless our analyses offer a starting point to dissect the TTFL of the SCN and generate a quantitative understanding of the clock.

**Figure EV5:**
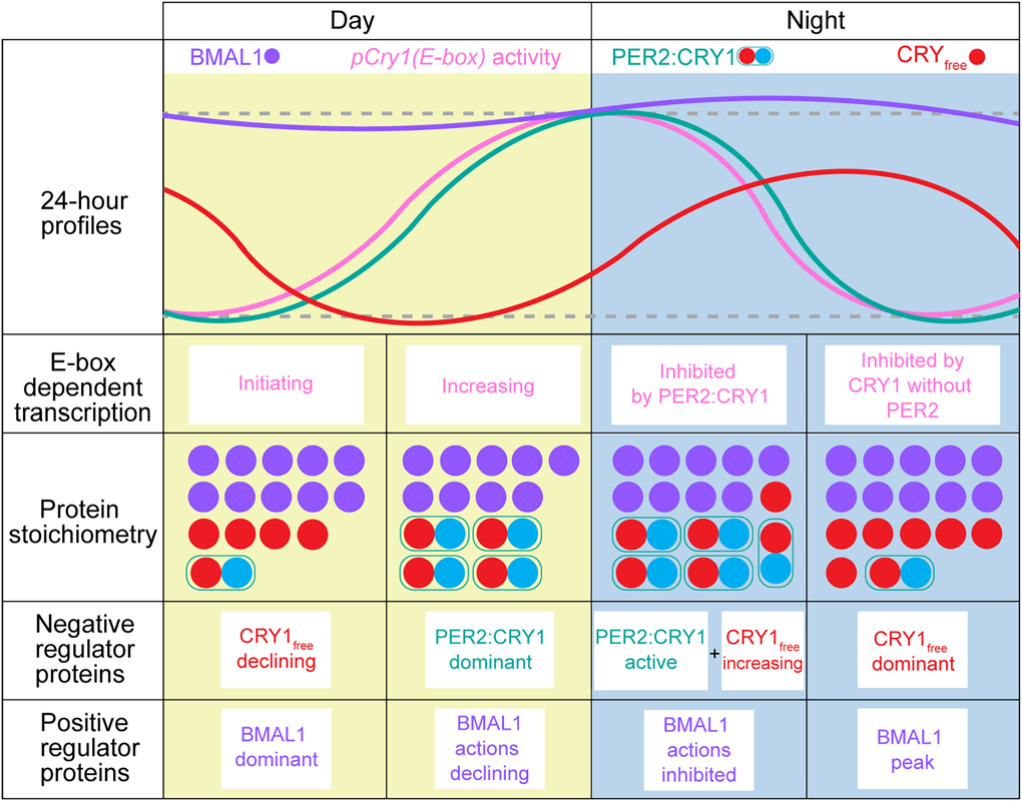
Schematic of TTFL protein and E-box activity in the SCN. Schematic shows the circadian dynamics of different TTFL activities. 1st row: plots of oscillations of (purple) BMAL1, (pink) *pCry1(Ebox)* promoter activity, (teal) PER2:CRY1 heteromeric complex and (red) free CRY1 outside of the PER2:CRY1 heteromeric complex. The minimum and maximum levels of E-box activation, suggested to be set by PER2 and CRY1_free_, respectively, are indicated with dotted grey lines. 2nd row: descriptions of E-box-dependent transcriptional activities, relating to the oscillation of *pCry1* promoter-activity. 3rd row: graphic depicting estimated and inferred integer stoichiometries of clock proteins. See main text and methods to see full description about assumptions made regarding calculations of PER2:CRY1 complexes. 4th row: status of negative regulator protein/ repressor complex activities. 5th row: status of positive regulator (BMAL1) activities. Rows 1–4 are divided into quarters of the circadian cycle: Early morning, late morning, early night and late night. The transition between day and night is CT12.

In the central pivot of the current TTFL model, PER:CRY heterodimer-containing protein complexes, mediate feedback repression of CLOCK:BMAL1-dependent trans-activation at E-boxes, thus depicting PER and CRY behaving “as one”, i.e., with a common location, timing and function. Consistent with this, at a qualitative level, PER2, CRY1 and BMAL1 co-localise in the nucleus of SCN cells and nuclear retention of PER2 in the SCN is controlled by CRYs and their ability to translocate to the nucleus (Smyllie et al, 2022). Nevertheless, our analyses highlight the inadequacy of this model. More than 25% of native PER2 is cytoplasmic, whereas CRY1 and BMAL1 are barely detectable outside the nucleus of SCN cells. Within the nucleus, a greater proportion of PER2 molecules remain mobile, compared with the larger immobile fractions of CRY1 and BMAL1. Thus, PER2 and CRY1 are not always together in intracellular space. Furthermore, in the SCN, steady-state PER2 and CRY1 oscillations are temporally segregated, the circadian peak of CRY1 lagging PER2 by ~7 h, a feature also observed in U2OS cells acutely treated with dexamethasone (Gabriel et al, 2021). The different temporal profiles of relative clock protein abundance contribute further to this misalignment. High-amplitude, low-baseline oscillations of PER2 molecular abundance rarely exceed that of CRY1, whereas CRY1 oscillates at and above the peak of PER2. Together, this highlights PER2 abundance as the main driver for PER2:CRY1 complex formation (Chen et al, 2009) during the middle of the circadian cycle, and points towards PER2-independent roles for CRY1_free_ during the late circadian night. Indeed, such a role for PER-independent CRY1_free_ is consistent with a two-stage model of E-box repression. Biochemical studies of the mouse liver TTFL suggest that CRY1-containing but PER-free “late” repressor protein complexes (Aryal et al, 2017) promote a transcriptionally “poised-state” (Koike et al, 2012) using a “blocking” mode of repression of CLOCK:BMAL1 at E-boxes (Ye et al, 2014). This contrasts with proposed “displacement”-type repression (Ye et al, 2014) by “early” PER2:CRY1-containing protein complexes (Aryal et al, 2017). In the SCN, the persistent presence of CRY1 suggests that the potential for E-box repression remains throughout the day, either as PER2:CRY1 or CRY1_free_, which could be modulated by the presence of corepressors. Further to this, the level of inhibition of CRY1_free_ could set the maximal level of E-box activation, whereas the abundance of PER2, which limits the availability of PER2:CRY1, may therefore set the minimum, i.e. less PER2 would give a higher minimum. The circadian timing of E-box activation/repression can be examined by mapping PER/CRY abundance in the context of *pCry1(Ebox)*-Luc oscillations. Peak *pCry1(E-box)* activity (~CT12.5) (Smyllie et al, 2016) is phase-aligned to PER2:CRY1_max_ abundance, when PER2:CRY1 repressive activity would likely dominate. In the late circadian night, with *pCry1(Ebox)*-Luc declining, the PER2/CRY1 ratio reaches a nadir, leaving CRY1_free_ available to engage in blocking-type repression of CLOCK:BMAL1 at the E-box (Fig. EV5). Thus, our data highlight how PER:CRY complex composition (and function) evolves progressively through circadian time.

Complementing this conclusion, selective drug-dependent stabilisation of PER or CRY1 revealed their overlapping and different functions. Dose-dependent period-lengthening of the TTFL by either PF670462 or KL101, strongly indicates that the stabilities of both proteins influence their repressive functions on the TTFL. The underlying mechanisms, however, were different because PER-stabilisation had little or no effect on the repression of E-box-dependent transcription, whereas CRY1-stabilisation dose-dependently increased repression, damped amplitude and suppressed oscillation baseline. This is consistent with genetic studies that identify global suppression of gene expression rhythms in CRY-stabilised FBXL mutants (Siepka et al, 2007) as well as CRY1 having greater repressive strength than PER2 through its high affinity for BMAL1 (Langmesser et al, 2008), acting as a PER-independent repressor. Importantly, selective stabilisation of either PER or CRY1 did not affect the stabilities of the other clock proteins. This argues against the widely held view of mutual protein stabilisation, which is commonly based on over-expression studies (Chen et al, 2009; Matsumura et al, 2022). Furthermore, the drug treatments also resulted in phase-specific extension of the descending phase that accounted for the lengthening of period. This is consistent with phase-specific sensitivity of PER2 and CRY1 to the actions of CK1δ and FBXL, respectively, and could explain observed circadian fluctuations in clock protein half-lives in U2OS cells (Gabriel et al, 2024). Importantly, this effect was selective because symmetrical waveform extension was observed in the non-targeted proteins. This “mismatch” in oscillation dynamics (asymmetrical/symmetrical) between TTFL proteins causes contrasting underlying molecular architectures and again emphasises the independence of PER and CRY1 protein stability and function. Finally, joint application of sub-saturating doses of PF670462 and KL101 had an additive effect on lengthening SCN period, indicative of independent effects of PER and CRY1 stabilisation. This provides mechanistic insight into genetic studies of circadian behaviour and SCN function (Maywood et al, 2011) that showed additive effects of mutations in key regulators within PER and CRY degradation pathways. Specifically, the dominant effect of PER2 stabilisation is to prolong the PER:CRY heterodimer activity phase in the early circadian night, whereas CRY1 stabilisation boosts the free-CRY1 activity phase in the late circadian night. Thus, PER and CRY1 (and BMAL1) stabilities are independent of each other, and PER and CRY1 proteins have different, complementary actions within the SCN TTFL.

Taken together, we have provided new quantitative insights within the SCN circadian clock, under physiological conditions, which are not described within, and are at odds with, the current processive model of the mammalian TTFL. Specifically, the activator and repressive arms of the TTFL are not static entities. Rather, complex spatiotemporal relationships exist within and between each arm, where CRY1 has additional independent behaviours outside of the PER:CRY heteromeric protein complex. This rich and unanticipated complexity to the dynamic behaviours of these circadian proteins provides a context and opportunity to develop new quantitative, multi-layered perspectives of the SCN clockwork.

## Methods

**Table:** Reagents and tools table

Reagent/Resource	Reference or Source	Identifier or Catalog Number
**Experimental models**		
C57.BL6 PER2::Luc *(M. musculus)*	(Yoo et al, 2004)	RRID: IMSR_JAX:006852
C57.BL6 *Cry1*-Luc *(M. musculus)*	(Maywood et al, 2013)	NA
C57.BL6 PER2::Venus *(M. musculus)*	(Smyllie et al, 2016)	NA
C57.BL6 CRY1::mRuby3 *(M. musculus)*	(Koch et al, 2022)	NA
C57.BL6 Venus::BMAL1 *(M. musculus)*	(Yang et al, 2020)	NA
C57.BL6 PER2::Venus/mRuby3 *(M. musculus)*	This paper	NA
**Recombinant DNA**		
AAV8.Ef1a-Cre	Vector Builder	VB230504-1362cwg
pCRE-TATA.dsELuc	Designed in this paper, made by Vector Builder	Vector ID: VB240116-1143qfx
LoxP( + 2 bp)-mVenus-STOP-LoxP( + 2 bp)-mRuby3	This paper	NA
**Antibodies**		
**Oligonucleotides and other sequence-based reagents**		
Primers for PER2::Venus/mRuby3 genotyping	See below	See below
**Chemicals, Enzymes and other reagents**		
PF670462	Sigma-Aldrich	Catalog: SML0795
KL101	Focus Biomolecules	Catalog: 10-3907
Cycloheximide	SLS	Catalog: 01810
DMSO	Santa Cruz Biotechnology	Catalog: 358801
MK801	Sigma-Aldrich	Catalog: M107
B27	GIBCO	Catalog: 0080085SA
D-AP5	Tocris	Catalog: 0106
Luciferin	Promega	Catalog: E6551
Paraformaldehyde	ThermoFisher Scientifc	Catalog: 043368.9M
**Software**		
ImageJ	https://imagej.net/software/fiji/	RRID:SCR_002285
Prism 9	Graphpad	RRID:SCR_002798
Web Statistics	https://github.com/tomoinn/web-statistics	N/A
Microsoft Excel	Microsoft	RRID:SCR_016137
Zen	Zeiss	RRID:SCR_013672

### Methods and protocols

#### Animals

All animals were cared for in accordance with the UK Animals (Scientific Procedures) Act (1986) with ethical approval granted by the UK Home Office, under the project licence number PP6024326. The MRC-LMB AWERB committee provided local ethical oversight. All mice were maintained on a C57/BL6 genetic background (>6 generations). PER2::Venus, CRY1::mRuby3 and *Cry1(E-box)*-Luc mice were generated in-house and Venus::BMAL1 mice were generated in collaboration with Qing-Jun Meng at University of Manchester (Yang et al, 2020). PER2::Luc mice originated from Joseph Takahashi from UT South Western (Yoo et al, 2004). PER2::Venus/mRuby3 mice were generated in-house.

#### Knock-in strategy for PER2::Venus/mRuby3 Colour-Switch

A CRISPR knock-in strategy, based on the CRISPR READI technique (Chen et al, 2019), was used to direct integration of LoxP-Venus-LoxP-mRuby3 in place of the stop codon of the *mPer2* gene to generate a C-terminally tagged PER2 allele. We designed sgRNA to target the sequences spanning the *Per2* stop codon, *ctgggggacagggttacgtc* and *tgggggacagggttacgtct* and had them synthesised full-length sgRNAs (Integrated DNA technologies). Note: sgRNA target sites were inactivated by transgene insertion, so locus re-editing post homology-directed repair would not be possible. For the donor, we designed a homology (500 bp each side) flanked integration cassette comprising of LoxP(+2 bp)-mVenus-STOP-LoxP(+2 bp)-mRuby3. Here, the LoxP can be recombined to alter the gene tag from mVenus to mRuby3. LoxP sequences have 2 bp added to act as in-frame linkers. Full donor sequence is provided below. This vector was cloned in an AAV backbone and packaged as rAAV6 (VectorBuilder, USA).

#### Donor sequence

(Box 1)

**Figure:**
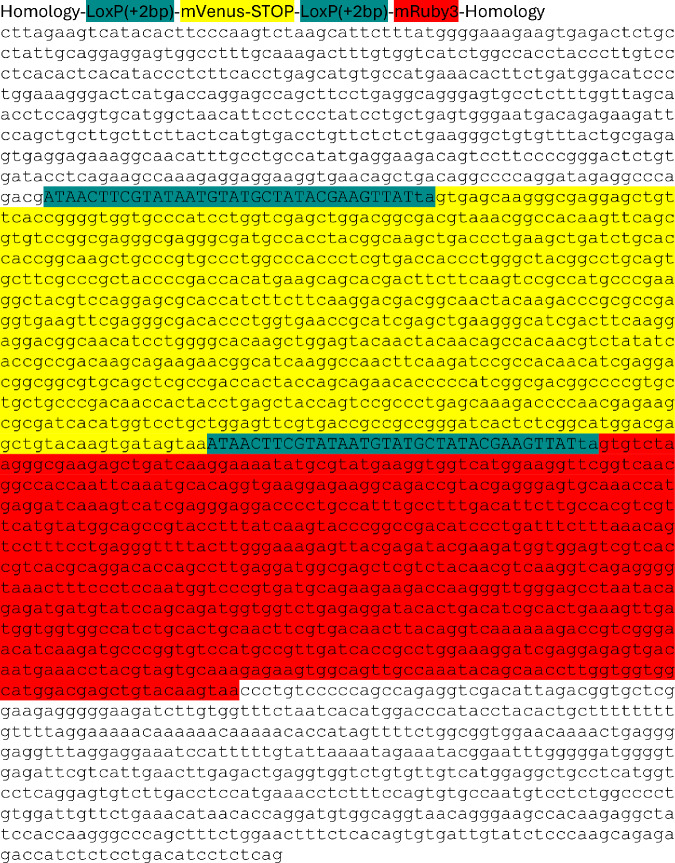

Cryopreserved 1-cell C57BL/6J embryos generated by IVF were thawed and cultured in AAV (minimum titre: >2 × 10^11^ GC/ml) containing medium (EmbryoMax KSOM + AA w/D-Glucose w/PhenolRed, Millipore) for 4 h. sgRNA oligos for each guide were resuspended in sterile, RNase-free Injection buffer (TrisHCl 1 mM, pH 7.5, EDTA 0.1 mM) at 1 μg/μl. 2.5 μg of each guide was mixed with 12.5 μg Engen Spy Cas9 recombinant protein (NEB M0646; final concentration: 500 ng/μl) and made up to a final volume of 20 μl in Opti-MEM (Gibco). The mixture was then incubated at room temperature for 10 min. The sgRNA:Cas9 mix was delivered to AAV infected embryos via electroporation using a protocol adapted from Teixeira et al (2018). Briefly, 15–30 zygotes were placed in 5 µl of sgRNA:Cas9 mix on an electrode slide (Nepagene CUY501P1-1.5) with the following settings:

Impedance: 120–180 Ω, 4 poring pulses @40 v, 3.5 ms, interval 50 ms, 10% voltage decay, Polarity +. 5 transfer pulses at 5 v, 50 ms, interval 50 ms, 40% voltage decay, alternating + and – polarity.

Zygotes were cultured overnight, and the resulting 2 cell embryos surgically implanted into the oviduct of day 0.5 post-coitum pseudo-pregnant mice, across 2 independent days. After birth (5 pups total) and weaning, genomic DNA was extracted using Sigma REDExtract-N-Amp Tissue PCR kit and used to genotype pups. A series of PCR reactions to determine transgene insertion (Fig. EV1) were performed using combinations of primers (shown below). Flanking PCRs identified three candidate knock-in pups (#2, 3 and 4, Fig. EV1B). Knock-in sequence fidelity for pup 4 was confirmed by Sanger sequencing (Fig. EV1C), and a colony established.

**Table:** 

Primer name	Primer sequence
AL34_gF	GCCATCTAGCATGCCTCTCC
AL34_gR4	ATGTTGACCACCACAGGCAG
mVenusF3	CGAAGGCTACGTCCAGGAG
mVenusR3	TGATGCCGTTCTTCTGCTTG
eGFP_seqF	GCAAAGACCCCAACGAGAAGC
mVenusR2	CCTCCTTGAAGTCGATGCCC

#### AAVs

AAV8.Ef1a-Cre (>2 × 10^11^ GC/mL) was obtained from VectorBuilder, off the shelf, Vector ID: VB230504-1362cwg. The pCRE-TATA.dsELuc construct was produced by VectorBuilder (Vector ID: VB240116-1143qfx). Briefly, the CRE promoter from the pCRE-DD-ZsGreen1 plasmid (2xCRE sequences fused to a TATA-like promoter region) as used previously (Brancaccio et al, 2013) was inserted into the standard VectorBuilder AAV backbone to drive expression of destabilised Emerald Luciferase (dsEluc). The Eluc sequence (from Addgene plasmid #170873) (González-Grandío et al, 2021) was destabilised by C-terminal fusion of the mouse ornithine decarboxylase (mODC) D433A/D434A (DA) variant PEST sequence (corresponding to amino acids 423-450 of the mODC protein), which has been shown to reduce the half-life of luciferase from 3 h to 0.8 h (Leclerc et al, 2000). The resulting plasmid was packaged into AAV serotype 1 viral particles (2.1 × 10^12^ GC/ml) by VectorBuilder.

#### SCN organotypic culture

SCN explants were prepared as previously described (Hastings et al, 2005; Smyllie et al, 2022) and briefly outlined below. Male and female mouse pups (P9-P12) were culled by a Schedule 1 method (dislocation and exsanguination). Brains were removed and placed into ice-cold dissection medium (GBSS (Sigma, USA); 5 mg/mL glucose) with 100 nM MK801 (Sigma, USA); 3 mM MgCl_2_ and 0.05 mM D-AP5 (Tocris, USA) to reduce excitotoxicity during slice preparation. The brains were carefully trimmed and then sliced at 300 μm thickness using a Mcllwain (UK) tissue chopper. Slices containing the SCN were further trimmed and transferred to membrane inserts (Millipore, USA) in dishes containing a tissue culture medium (50% Eagle’s basal medium (Sigma, USA); 25% EBSS (Sigma; 25% heat inactivated horse serum (Invitrogen, USA); 5 mg/mL D-glucose (Sigma); 25 μg/mL Penicillin/Streptomycin; 1% Glutamax (Invitrogen, USA); pH 7.2, osmolarity 315–320 mOsm), which was again supplemented with MK801, MgCl_2_ and D-AP5. SCN slices were maintained at 37 °C, 5% CO_2_. After 2–4 h in culture, slices were transferred from supplemented culture medium to culture medium without excitotoxicity blockers for long-term maintenance. SCN slices were maintained in culture for 7 days prior to luciferase-based recordings or 3 days prior to fluorescence imaging experiments.

#### Luciferase recordings

SCN slices were transferred to 35 mm cell culture dishes containing 1.2 mL of recording medium (D-MEM (Sigma); 0.35 mg/mL NaHCO_3_ (Fisher Scientific); 5 mg/mL glucose; 25 μg/mL; Penicillin/Streptomycin; 0.01 M HEPES (Invitrogen)) made up as a stock solution and further supplemented with foetal calf serum (Gibco, USA), B27 (Gibco), Glutamax (Invitrogen) and 10 μM Luciferin (Promega, USA)). A coverglass was sealed over the dishes using silicone grease to prevent evaporation during the recording. Luciferase bioluminescence signal was detected by photon multiplier tubes (PMT; Hamamatsu, Japan), housed within a light-tight incubator maintained at 37 °C. Photons were counted every second and collected into 6-min bins.

#### Fixed tissue preparation

SCN slices were first cut out of their membrane inserts and fixed in 4% PFA (Thermo Scientific, USA) in phosphate buffer for 30 min at room temperature, with gentle shaking. The fixed SCN slices were then washed with PBS for 15 min, 3 times and mounted onto frost-free slides (Thermo Scientific) in a DAPI-containing Vectashield Hardset mounting medium (Vector Labs, USA).

### Confocal microscopy procedures

#### Confocal imaging of fixed SCN samples

Fixed SCN tissue (sections of adult brain or neonatal brain slices) were imaged using a Zeiss LSM780 system using a 63X oil immersion apochromatic objective. The tile-scan mode was used to acquire the entire SCN and the images were stitched together in the Zen software. A 10% overlap was included to reduce edge artifacts. Nonetheless, some tile-scan artifacts remain present in the images. Further image processing was carried out within FIJI software.

#### Confocal timelapse recordings

SCN slices were transferred into 35 mm glass bottom dishes (Mattek, USA) and sealed with a coverglass as previously described (Smyllie et al, 2022). Live confocal imaging was carried out using a Zeiss LSM880 inverted system, maintained at 37 °C. A custom dish holder allowed imaging of 6 SCN slices at any time. A 10X apochromatic objective was used and the following acquisition settings used in the Zeiss Zen software: 1024 × 1024 pixels frame, 4x averaging, 1 frame acquired per 30 min.

#### CRY1::mRuby3 confocal FRAP

FRAP experiments on SCN slices were carried out as previously described (Smyllie et al, 2022; Smyllie et al, 2016) and briefly summarised below. A 63x apochromatic objective was used on a Zeiss LSM880 inverted system, maintained at 37 °C. To bring the SCN slice into the working distance range of the objective, the SCN slice was excised from its membrane and place directly onto a 35 mm glass bottom dish containing a minimal amount of recording medium (~100 μL). The slice was held in place using a slice harp (Scientifica, UK) to ensure stable imaging. To reduce the effect of photobleaching, only the regions of interest (ROI) were imaged rather than acquiring full frame recordings. The following FRAP protocol was used: 20 frames baseline, 10 frames photobleach at 100% intensity using 514 nm laser, 100 s acquisition of fluorescence recovery. In addition to the FRAP ROI, a background and control (cell without photobleaching) were recorded to account for background fluorescence and acquisition photobleaching.

### Pharmacological manipulations

#### Half-life measures

To measure PER2, CRY1 and BMAL1 protein half-lives, 40 μg/mL cycloheximide (CHX) (Sigma-Aldrich, USA) was applied to SCN slices to inhibit protein translation. The CT of application depended on the combination of proteins being measured: CT 15 for PER2::Venus with CRY1::mRuby3, and CT 19 for CRY1::mRuby3 and Venus::BMAL1 simultaneous recordings. Fluorescence timelapse recordings of the slices during CHX were made using a Zeiss LSM880, either every 15 or 30 min for at least 60 h to capture full fluorescence decay.

#### Manipulating protein stability

PF670462 (Sigma) was used to stabilise PER protein and KL101 (Focus Molecules, USA) was used to stabilise CRY1 protein. Dose–response curves were generated by treating SCN slices with a range of concentrations of PF670462 from zero (0.1% DMSO) to 1 μM and KL101 from zero (0.1% DMSO) to 10 μM. To ensure that our measured effects were “on target” and not related to cytotoxicity, only doses where the measured effects were immediately and fully reversible on washout of the drug were used. Dose ranges for protein stabiliser drugs were selected based on previous literature (Meng et al, 2010; Patton et al, 2016; Miller et al, 2020), where no cytotoxic effects were reported. PER2::Luc, *Cry1(Ebox)*-Luc and *CRE-TATA*-Luc reporter bioluminescence was detected by PMTs (as described above). These experiments followed the following protocol: baseline (pre) recording for 7 days, treatment for 7 days, washout (medium change) and record for a final 7 days. In addition to luciferase recordings, the effect on PER2, CRY1 and BMAL1 protein oscillations was observed using timelapse confocal microscopy (as described above). Here, the following protocol was used: baseline recording for 3–7 days, treatment for 4–7 days, CHX treatment (at CT 15 or CT 18) for 60 h.

#### PER2-Colourswitch confocal recordings

The P_cs_-KI mouse was used to calibrate the differences in fluorescence properties between mVenus and mRuby3, thus allowing relative abundance measures between all 3 proteins. Dual-fluorescence timelapse recordings of PER2-colourswitch were made using a Zeiss LSM880. Both mVenus and mRuby3 channels were recorded from P_cs_-KI SCN slices for at least 3 cycles, prior to transduction with a pan-Cre recombinase: *Ef1a*-Cre (Vector Builder). The recording was then continued for at least a further 7 days to allow complete loss of the mVenus sequence and to allow maximum expression of PER2::mRuby3.

### Analysis methods

#### Nucleus:Cytoplasmic (Nuc:Cyto) ratio

Fluorescence Intensity was measured within FIJI software (NIH, USA). All measures were background-corrected using fluorescence intensity outside of the SCN area. ROIs were selected for nucleus and cytoplasm for each cell. The Nuc:cyto ratio was then calculated for the background-subtracted intensities and expressed as percentages.

#### FRAP

Background subtraction and acquisition bleach correction were applied within the Zen software prior to FRAP curve fitting. Data were fit to the best fit model: a two-component, passive diffusion model:

$$I={I}_{E}-{I}_{1}{\exp }^{-\frac{t}{{T}_{1}}}-{I}_{2}{\exp }^{-\frac{t}{{T}_{2}}}$$

*I* = intensity at time, *t;I*_*E*_ = end intensity (after recovery)*; I*_1,2_ = intensity from component 1 or 2*; T*_1,2_ = constant for component 1 or 2.

The Zen software uses this equation and the equation below to calculate the *t*_1/2_ of recovery:

$${t}_{1/2}={ln}2\cdot {T}_{x}$$

(where *T*_*x*_ is either *T*_1_ or *T*_2_, giving a *t*_1/2_ value for CRY1_fast_ and CRY1_slow_ fractions).

The *t*_1/2_ values were converted to diffusion coefficients using the Axelrod equation (Axelrod et al, 1976) to account for variation in bleach area:

$$D=\frac{{w}^{2}}{4\cdot {t}_{1/2}}$$

*D* = diffusion coefficient and *w* = bleach diameter.

Mobile and immobile molecule fractions were expressed as percentages using the magnitude of fluorescence recovery (*I*_*E*_*– I*_0_) in relation to the pre-bleach fluorescence (*I*_1_*– I*_0_).

$$\% {{CRY}1}_{{mobile}}=\left(\frac{{I}_{E}-{I}_{0}}{{I}_{1}-{I}_{0}}\right)\cdot 100$$

*I*_1_ = Initial intensity (pre-FRAP) and *I*_0_ = intensity at *t* = 0 after photobleaching.

Each mobility fraction is given by the following equations:

$$\% {{CRY}1}_{{Fast}}=\left(\frac{{I}_{1}}{{I}_{E}-{I}_{1}-{I}_{2}}\right)\cdot 100$$

$$\% {{CRY}1}_{{Slow}}=\left(\frac{{I}_{2}}{{I}_{E}-{I}_{1}-{I}_{2}}\right)\cdot 100$$

#### Luciferase circadian analyses

Period and relative amplitude error (RAE—a measure of rhythm robustness) of bioluminescence oscillations were calculated using a fast-fourier transform – linear non-least squares (FFT-NLLS) algorithm within the BioDare circadian rhythms analyses platform (biodare2.ed.ac.uk) (Zielinski et al, 2014). The first 24 h of recording were excluded from analyses and at least 5 days of recording were analysed. For PF670462 and KL101 dose–response curves, the effect on oscillation baseline and amplitude were measured. The baseline of the first full cycle during treatment was expressed as percentage of the projected baseline of the last cycle prior to treatment. Similarly, the amplitude of the oscillation on the first full cycle during treatment was expressed as the percentage of the projected amplitude of the last cycle prior to treatment.

#### Fluorescence circadian analyses

All circadian analyses of fluorescence timelapse recordings were carried out within Graphpad Prism software (v10; USA) using a “Peak-to-Peak” method. Raw data were first de-trended using a centred fifth exponential fit (unbiased detrending). “Area under the Curve” was then used to define peaks that were >10% above baseline. The time interval between the peaks was calculated as the period on a cycle-by-cycle basis.

#### Peak circadian phase

Oscillations in fluorescence intensity (mean average across the field-of-view) from dual-channel confocal timelapse recordings were used to make measures of peak phase of each protein from PC-KI and BC-KI SCN. To map the peak circadian phase of all three clock proteins, PER2::Venus was used as a primary phase reference, defined as CT12 (Smyllie et al, 2016). From this, CRY1 phase was defined and used to measure the phase of BMAL1 in the BC-KI SCN. The calculated peaks times (CT) for each protein in each slice were graphed in a Rayleigh circular plot using an open-source analysis algorithm (https://github.com/tomoinn/web-statistics).

#### Average circadian profiles

At least 4 circadian cycles of fluorescence were used for each protein in each slice to create their average circadian profile. Circadian profiles were generated using either raw or normalised data, specified below:

Figure 2: normalised to peak and trough of oscillation.

Figure 4C: normalised to peak and zero point for each protein (calculated from CHX treatments. Note: see below for CHX decay curves).

Figure 4F,G: using background-subtracted fluorescence intensity.

Figure 4I,J: expressed as percentage relative to PER2 (mVenus or mRuby3) fluorescence, where 0% is the PER2 “zero point”, 20% is the PER2 oscillation trough and 120% is the PER2 oscillation peak. Circadian profiles of proteins in individual slices were averaged across multiple slices (*n* number shown on figure).

Figure 4K: expressed as a ratio using circadian profiles from Fig. 4C.

Circadian profiles shown in Fig. 4J are inferred from abundance data of PER2 and CRY1. They represent a theoretical abundance of different molecular species using the following assumptions:

- *Assumption 1: PER2 and CRY1 heterodimerise, if possible, with a 1:1 stoichiometry.*
- *Assumption 2: The amount of PER2:CRY1 will be limited by the lowest (MIN) concentration of either PER2 or CRY1*.

and Equations:

- PER2:CRY1_max_ is the abundance (given assumptions) of PER2:CRY1 heterodimers given by:
  $${({{\rm{PER}}}2\!\!:{{\rm{CRY}}}1)}_{\max {{\rm{t}}}={{\rm{x}}}}={{\rm{MIN}}}({{{\rm{PER}}}2}_{{{\rm{t}}}={{\rm{x}}}},{{{\rm{CRY}}}1}_{{{\rm{t}}}={{\rm{x}}}})$$
- PER2 + CRY1 is the total abundance of PER2 and CRY1 as a combined pool given by:

$${({{\rm{PER}}}2+{{\rm{CRY}}}1)}_{{{\rm{t}}}={{\rm{x}}}}={{{\rm{PER}}}2}_{{{\rm{t}}}={{\rm{x}}}}+{{{\rm{CRY}}}1}_{{{\rm{t}}}={{\rm{x}}}}$$

- PER2_free_ is the abundance of remaining PER2, after considering preferential inclusion of PER2 in PER2:CRY1 heterodimers, given by:
  $${{{\rm{PER}}}2}_{{{\rm{free\; t}}}={{\rm{x}}}}={{{\rm{PER}}}2}_{{{\rm{total\; t}}}={{\rm{x}}}}-({{\rm{MIN}}}\left({{{\rm{PER}}}2}_{{{\rm{total\; t}}}={{\rm{x}}}},{{{\rm{CRY}}}1}_{{{\rm{total\; t}}}={{\rm{x}}}}\right))$$
- CRY1_free_ is the abundance of remaining CRY1, after considering preferential inclusion of CRY1 in PER2:CRY1 heterodimers, given by:

$${{{\rm{CRY}}}1}_{{{\rm{free\; t}}}={{\rm{x}}}}={{{\rm{CRY}}}1}_{{{\rm{total\; t}}}={{\rm{x}}}}-({{\rm{MIN}}}\left({{{\rm{PER}}}2}_{{{\rm{total\; t}}}={{\rm{x}}}},{{{\rm{CRY}}}1}_{{{\rm{total\; t}}}={{\rm{x}}}}\right))$$

Note: We use heterodimer to describe the PER2:CRY1 interaction. PER2:CRY1 will exist in larger multimeric protein complexes and not in isolation.

#### Relative phase relationship calculations

Phase relationships between PER2 and CRY1 or CRY1 and BMAL1 were calculated under control conditions (before treatment) and during treatment of vehicle (0.1% DMSO) 1 μM PF670462 or 10 μM KL101. For each slice recording, the peak phase difference, or peak phase Δ between the pairs of proteins, was calculated on a cycle-by-cycle basis. The “before treatment” and “during treatment” phase Δ were then averaged separately and compared using paired statistical tests (statistics described later).

#### CHX decay curves

To calculate protein half-life, CHX decay curves were fit to a “Plateau followed by one phase decay” model within Graphpad Prism using the following equation:

$$Y={if}(X \, < \, {X}_{0},{Y}_{0},{Plateau}+\left({Y}_{0}-{Plateau}\right)* \exp \left(-K* \left(X-{X}_{0}\right)\right))$$

*X* = time. *Y* = *Y*_0_ until *X* = *X*_0_ the time at which fluorescence starts to decay down to the plateau with a one-phase decay. *Y*_0_ and Plateau are the same units as *Y*. *K* = rate constant (units are reciprocal to X axis units).

#### Protein oscillation baseline and “protein zero point”

CHX treatment was used to calculate the fluorescence intensity where protein molecules equal zero: “protein zero point”. This is equivalent to the plateau in the equation as defined above. The defined zero point provided a fixed point to make relative measures of oscillation baseline, by discounting background fluorescence. The oscillation baseline was calculated using the following equation:

$$\% {Tr}=\frac{{F}_{{tr}}}{({F}_{{tr}}-{F}_{0})}* 100$$

*Tr* = Baseline. *F*_*tr*_ = absolute fluorescence at oscillation trough. *F*_0_ = absolute fluorescence at zero point, or plateau of CHX treatment.

#### Phase maps

Phase maps were generated as previously described (Patton et al, 2020). Briefly, dual timelapse fluorescence image stacks were split into individual colour channels in FIJI software. The same mask was applied to both channels to remove extra-SCN signal. A custom FIJI plug-in was used to generate a grid of regions of interest (ROIs) over the SCN. Mean fluorescence timeseries for each grid square were extracted. The grid of fluorescence timeseries data were analysed using FFT-NLLS in the BioDare software, as described above. Phase information from the analysis was converted to CT by using the average period of the ROIs. The phase difference between fluorescence channels of the same recording were calculated for each grid-square. The phase information for the grid square was then loaded into Graphpad Prism and assembled into phase maps using the heat-map plot function. The colour ranges were set to reflect the range of phases for the given protein, in order to reveal any spatiotemporal dynamics that are present.

#### Timelapse projections

To compare SCN-level protein localisation of PER2, CRY1 and BMAL1, “SUM projection” was used, which sums the fluorescence of all frames in the images stack. The SUM projections of each fluorescence channel were then compared to one another (PER2 vs CRY1 or CRY1 vs BMAL1). The SUM projections were subtracted from one another to observe protein-specific localisation, e.g., PER2 without CRY1 (PER2-only) or CRY1 with PER2 (CRY1-only).

#### Waveform analysis

The effect of PF670462 and KL101 on the waveform of protein oscillations was measured by analysing peak width and trough width. Here, the width of each peak/trough was measured at the 50% distance from either midpoint to trough or midpoint to peak. At least 4 cycles were measured per slice and averaged. The widths were expressed as a percentage of the pre-treatment peak-width.

### Statistics

All graphs and statistics were performed in Graphpad Prism. Graphs that show group data include error bars expressing standard error of the mean (SEM). Table 1 outlines the statistical tests and *n* numbers for each figure.

**Table 1:** Summary of statistical tests for each figure.

Figure	# groups	# SCN (biological replicates)	Test	Comparison
1E	3	>21	One-way ANOVA	Tukey’s
1G	3	>12 per group	One-way ANOVA	Tukey’s
1H	3	>12 per group	One-way ANOVA	Tukey’s
Appendix Fig. S2B	4	>17	One-way ANOVA	Tukey’s
Appendix Fig. S2C	3 × 4	>2	Two-way ANOVA	Tukey’s
Appendix Fig. S2D	6	>2	One-way ANOVA	Tukey’s
Appendix Fig. S2E	2	>10	Unpaired t-test	n/a
Appendix Fig. S2G	2 per graph	>6 per group	Unpaired t-test	n/a
Appendix Fig. S2H	2 per graph	>6 per group	Unpaired t-test	n/a
2D	3	>22 per group	One-way ANOVA	Tukey’s
Appendix Fig. S3A	2	>22 per group	Paired t-test	n/a
Appendix Fig. S3B	2	>22 per group	Paired t-test	n/a
Appendix Fig. S3C	2	>22 per group	Unpaired t-test	n/a
Appendix Fig. S3D	4	>22 per group	One-way ANOVA	Tukey’s
Appendix Fig. S3F	3	>22 per group	One-way ANOVA	Tukey’s
3B	2	>2 per group	Unpaired t-test	n/a
4B	3	>11 per group	One-way ANOVA	Tukey’s
4H	3	>2 per group	One-way ANOVA	Tukey’s
4I	3	>2 per group	One-way ANOVA	Tukey’s
4M	3	>6 per group	Brown-Forsythe and Welch’s ANOVA test	Dunnet’s
Appendix Fig. S4B	2	>2 per group	Unpaired t-test	n/a
Appendix Fig. S4C	2	>2 per group	Unpaired t-test	n/a
EV1D	2	6 per group	Paired t-test	n/a
Appendix Fig. S5B	2	>7 per group	Unpaired t-test	n/a
Appendix Fig. S5C	4	>7 per group	One-way ANOVA	Tukey’s
Appendix Fig. S5D	2 × 3	>7 per group	Two-way ANOVA	Šidák’s
5C	3 × 2	>5 per group	Two-way ANOVA	Šidák’s
5D	3 × 2	>4 per group	Two-way ANOVA	Šidák’s
5E	2 × 2	>4 per group	Two-way ANOVA	Šidák’s
5F	2 × 2	>4 per group	Two-way ANOVA	Šidák’s
5H	6	>5 per group	One-way ANOVA	Dunnet’s
5I	6	>5 per group	One-way ANOVA	Dunnet’s
5J	6	>5 per group	One-way ANOVA	Dunnet’s
EV2D	6	>4 per group	One-way ANOVA	Dunnet’s
6C	3 × 2	>4 per group	Two-way ANOVA	Šidák’s
6D	2 × 2	>8 per group	Two-way ANOVA	Šidák’s
6E	2 × 2	>4 per group	Two-way ANOVA	Šidák’s
6F	2 × 2	>4 per group	Two-way ANOVA	Šidák’s
6H	6	>4 per group	One-way ANOVA	Dunnet’s
6I	6	>4 per group	One-way ANOVA	Dunnet’s
6J	6	>4 per group	One-way ANOVA	Dunnet’s
EV3D	6	>5 per group	One-way ANOVA	Dunnet’s
7B	4	>5 per group	One-way ANOVA	Tukey’s
7C	4	>5 per group	One-way ANOVA	Tukey’s
7D	4	>5 per group	One-way ANOVA	Tukey’s
7E	4	>9 per group	One-way ANOVA	Tukey’s
7F	4	>9 per group	One-way ANOVA	Tukey’s
7G	4	>9 per group	One-way ANOVA	Tukey’s
EV4B	4	>5 per group	One-way ANOVA	Tukey’s
EV4D	4	>9 per group	One-way ANOVA	Tukey’s

## Supplementary information

Appendix

Peer Review File

Source data Fig. 1

Source data Fig. 2

Source data Fig. 3

Source data Fig. 4

Source data Fig. 5

Source data Fig. 6

Source data Fig. 7

Expanded View Figures

## Data Availability

This study includes data deposited onto the Bioimage Archive (S-BIAD1582), link: https://www.ebi.ac.uk/biostudies/bioimages/studies/S-BIAD1582?key=fa96f5e0-48b5-44b1-85ec-a37b07263a64. All data reported in this paper is included in the source data files. This paper does not report original code. Further information required to re-analyse the data reported in this paper is available from the lead contact upon request. In this study we generated a new mouse line: PER2::Venus/mRuby3 Colour-Switch (P_cs_-KI). These animals will be shared by the lead contact on request, subject to material transfer agreement. We also generated a new AAV construct: *CRE-TATA-Luc*, whose full details for getting this made from Vector Builder are included in this paper. Any further information and requests for resources and reagents should be directed and will be fulfilled by the lead contact: Michael H. Hastings mha@mrc-lmb.cam.ac.uk. The source data of this paper are collected in the following database record: biostudies:S-SCDT-10_1038-S44318-025-00426-z.
