# Supplementary material for: Quantitative measures of clock protein dynamics in the mouse suprachiasmatic nucleus extends the circadian time-keeping model
Source: EMBO J. 2025 Apr 17;44(13):3614–44. doi: 10.1038/s44318-025-00426-z (PMC12218236; doi:10.1038/s44318-025-00426-z)
Supplement: Supplementary file 1 — Appendix [file 44318_2025_426_MOESM1_ESM.pdf]

Appendix for: “Quantitative measures of clock protein dynamics in the mouse suprachiasmatic nucleus extends the circadian time-keeping model”

Table of contents:

Appendix Figure S1	page 2
Appendix Figure S2	page 4
Appendix Figure S3	page 5
Appendix Figure S4	page 8
Appendix Figure S5	page 10

Appendix Figure S1

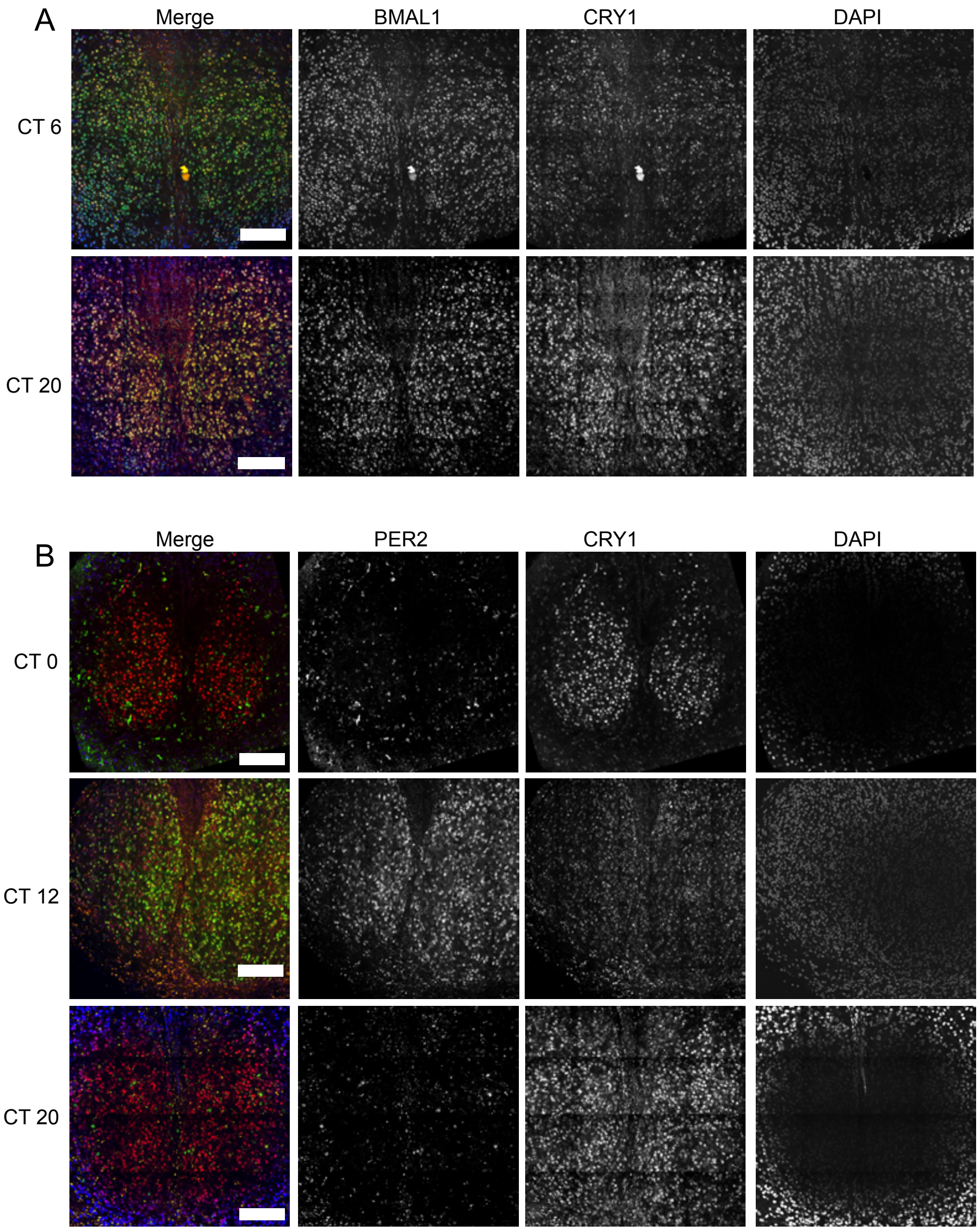

Appendix Figure S1. Endogenous clock proteins in the SCN of double KI mice: PC-KI and BC-KI

Confocal images show fluorescence of endogenous clock protein – fluorescent protein fusions in fixed SCN slices from either **A**, the PC-KI mouse line that expresses PER2::Venus (green) and CRY1::mRuby3 (red) or **B**, the BC-KI mouse line that expresses Venus::BMAL1 (green) and CRY1::mRuby3 (red). Slices were co-stained with DAPI (blue). The circadian time at which the slices were fixed (based on prior recording of a circadian luciferase reporter) is shown on the left side of the image panels. The individual channels are shown in greyscale to the right of the merge image. Scale bar = 200 μm.

Appendix Figure S2

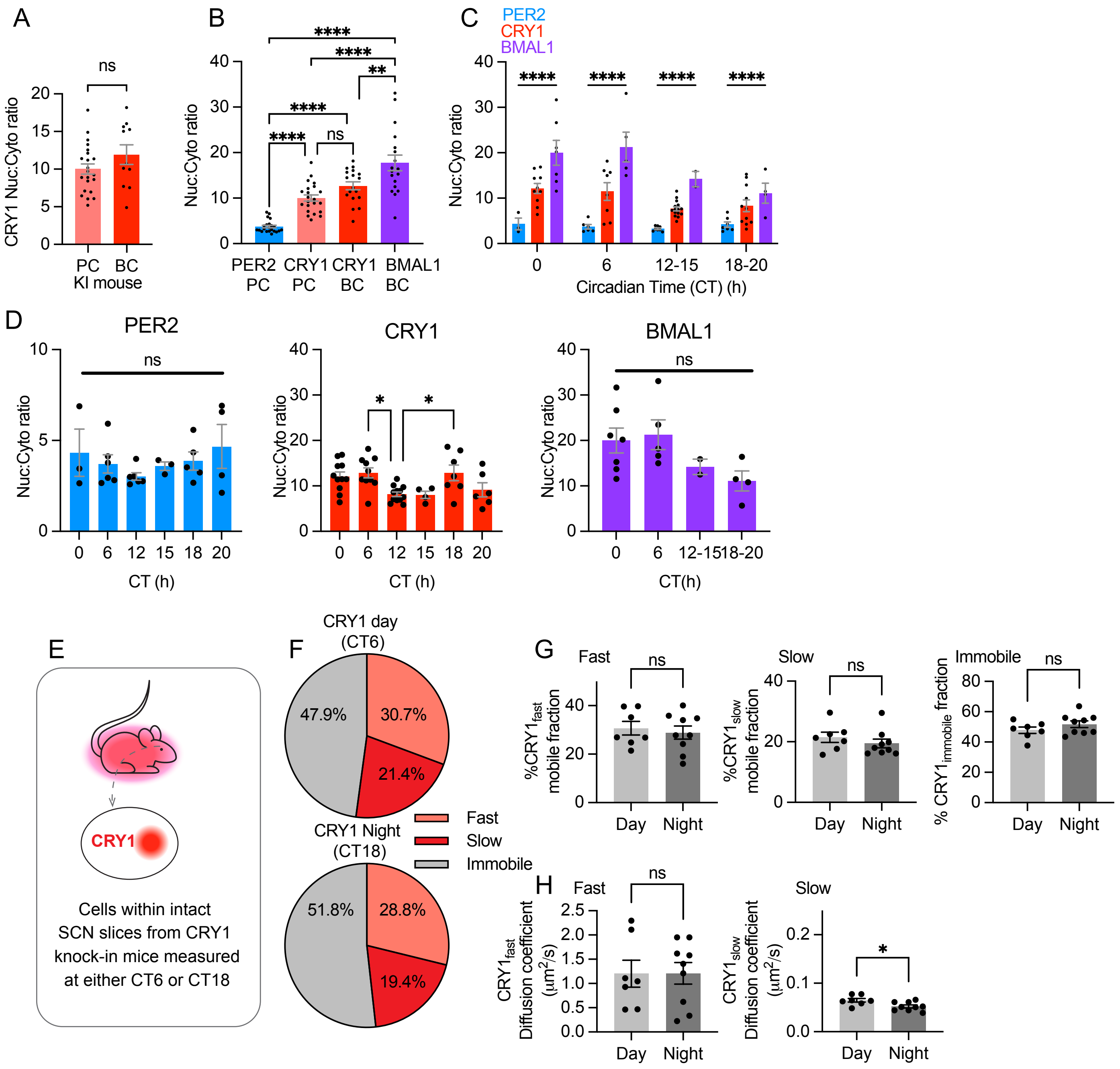

Appendix Figure S2. Intracellular behaviours of endogenous clock proteins

A-D, Group data show Nuclear:Cytoplasm (Nuc:Cyto) ratios of clock proteins measured in cells within fixed SCN slices. **A**, Nuc:Cyto ratio of CRY1 in time-of-day-matched SCN slices taken from either the PC-KI or BC-KI mouse lines (n > 10 SCN per group; unpaired t-test: ns, P = 0.164. **B**, Nuc:Cyto ratio of all three proteins, accounting for KI mouse origin for CRY1 measures (n > 18 SCN per group; ns, P = 0.204, ****P <0.0001, **P = 0.0038,). **C-D**, Nuc:Cyto ratio measures of clock proteins at different times across the circadian cycle (n > 2 SCN per protein per time point). **c**, Comparison between proteins at each time point (two-way ANOVA with Tukey's multiple comparison test: ****P < 0.0001). **D**, Circadian analysis of Nuc:Cyto ratio for (left) PER2 (ns, P = 0.579), (middle) CRY1 (**P = 0.0033) and (right) BMAL1 (ns, P = 0.116). A Tukey's multiple comparison test showed significant variation of CRY1 localisation between CT12 and CT6 (*P = 0.0166) and CT18 (*P = 0.0424). **E**, Schematic diagram indicating that CRY1 intra-nuclear molecular mobility was measured in cells within SCN slices from CRY1::mRuby3 single KI mice. **F**, Pie charts showing the mean average (n > 6 SCN per group) proportions of CRY1 molecules that occupy fast, slow and immobile pools during circadian day (upper) or circadian night (lower). **G,** There was no circadian day (CT6) / night (CT18) variation in the percentages of CRY molecules that are defined as (left) $CRY1_{fast}$ (ns, P = 0.655), (middle) $CRY1_{slow}$ (ns, P = 0.375) or (right) $CRY1_{immobile}$ (ns, P = 0.22) mobility pools (n > 6 SCN per group). **H**, There was no circadian day / night variation in diffusion coefficients of $CRY1_{fast}$ (ns, P = 0.981), but a small significant difference for $CRY1_{slow}$ (*P = 0.019) (n > 6 SCN per group). All group data in this figure are presented as mean ± SEM. Data in **B and D** were analysed by One-way ANOVA with Tukey's comparisons test and data in **G-H** were analysed with unpaired t-tests.

Appendix Figure S3

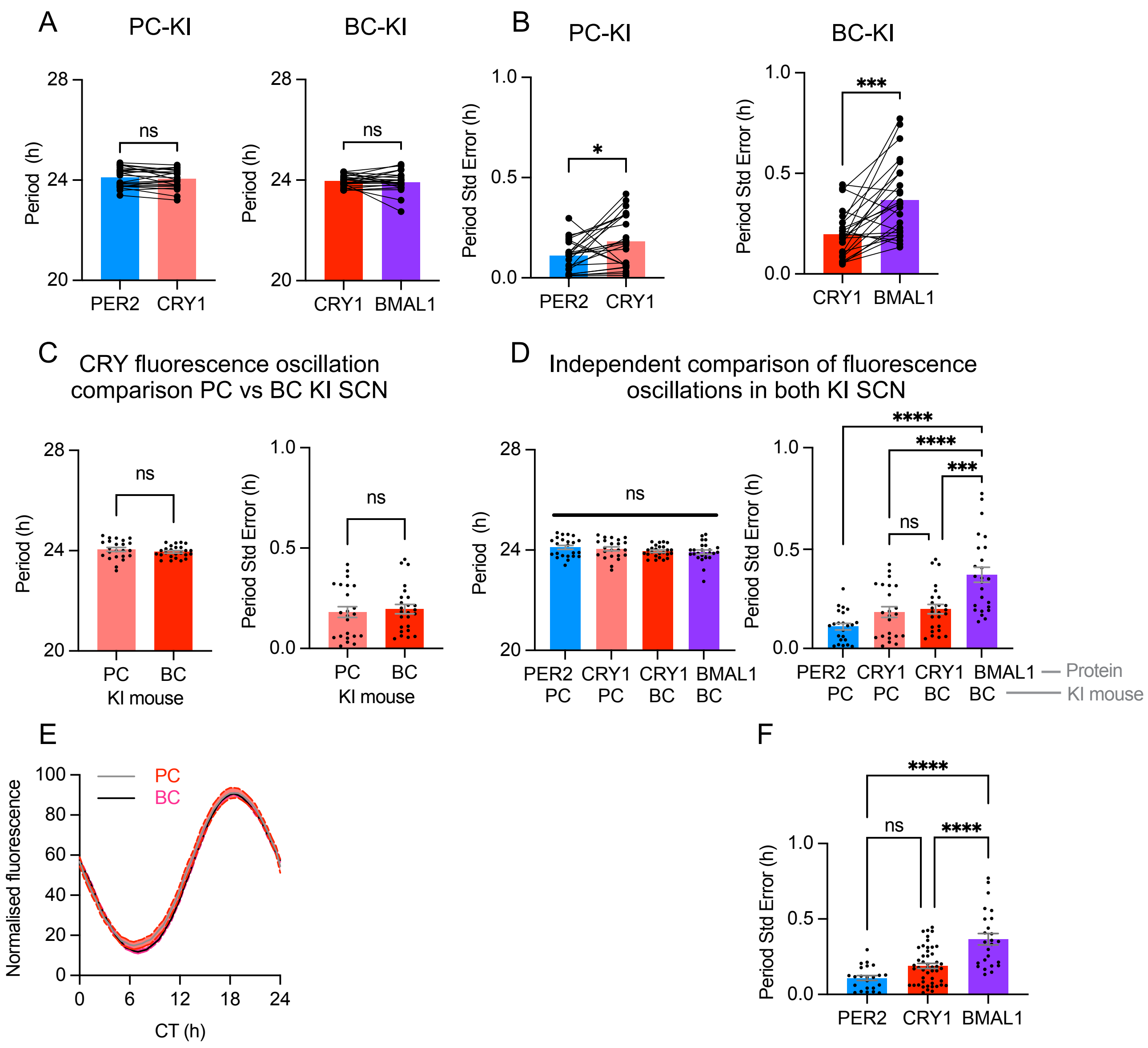

Appendix Figure S3. Circadian dynamics of endogenous clock proteins fusions in SCN slices from KI mice

A, Circadian periods of (left) PER2 and CRY1 (paired t-test: ns, P = 0.232) and (right) BMAL1 and CRY1 fluorescence oscillations within the same SCN were not significantly different (paired t-test: ns, P = 0.64). **B**, Standard (Std) error of (left) CRY1 period was significantly greater than PER2 (paired t-test: ns, *P = 0.0156) and (right) the Std error of BMAL1 period was significantly greater than CRY1 (paired t-test: ***P = 0.001) within the same SCN slices. Low Std error was indicative of a greater robustness in the oscillations. **c**, Comparison of CRY1 circadian properties in SCN slices from PC-KI and BC-KI mice, using data shown in **A-B**. Period (left) and period Std error (right) of CRY1 fluorescence oscillations were not significantly different between the two mouse lines ($n_{PC\text{-}KI}$ = 23, $n_{BC\text{-}KI}$ = 25 SCN; unpaired t-test: period: ns, P = 0.314; period Std error: P = 0.674). **D**, Independent analyses of (left) period (One-way ANOVA: ns, P = 0.246) and (right) period Std error (One-way ANOVA with Tukey's comparisons test: ns, P > 0.1, ***P = 0.0001, ****P < 0.0001) of data shown in **A-C**. **E**, Average 24-hour CRY1 oscillation profiles of SCN taken from either PC-KI (grey and red) or BC-KI (black and pink) mice. Oscillation plotted as mean (unbroken line, grey or black) ± SEM (dashed line, red or pink). **F**, Independent analysis of period Std error of oscillations of all three proteins irrespective of mouse KI origin (One-way ANOVA with Tukey's comparisons test: ns, P = 0.0517, ****P < 0.0001). All group data in this figure are presented as mean ± SEM. 23 PC-KI SCN and 25 BC-KI SCN slices were used for all analyses.

Appendix Figure S4

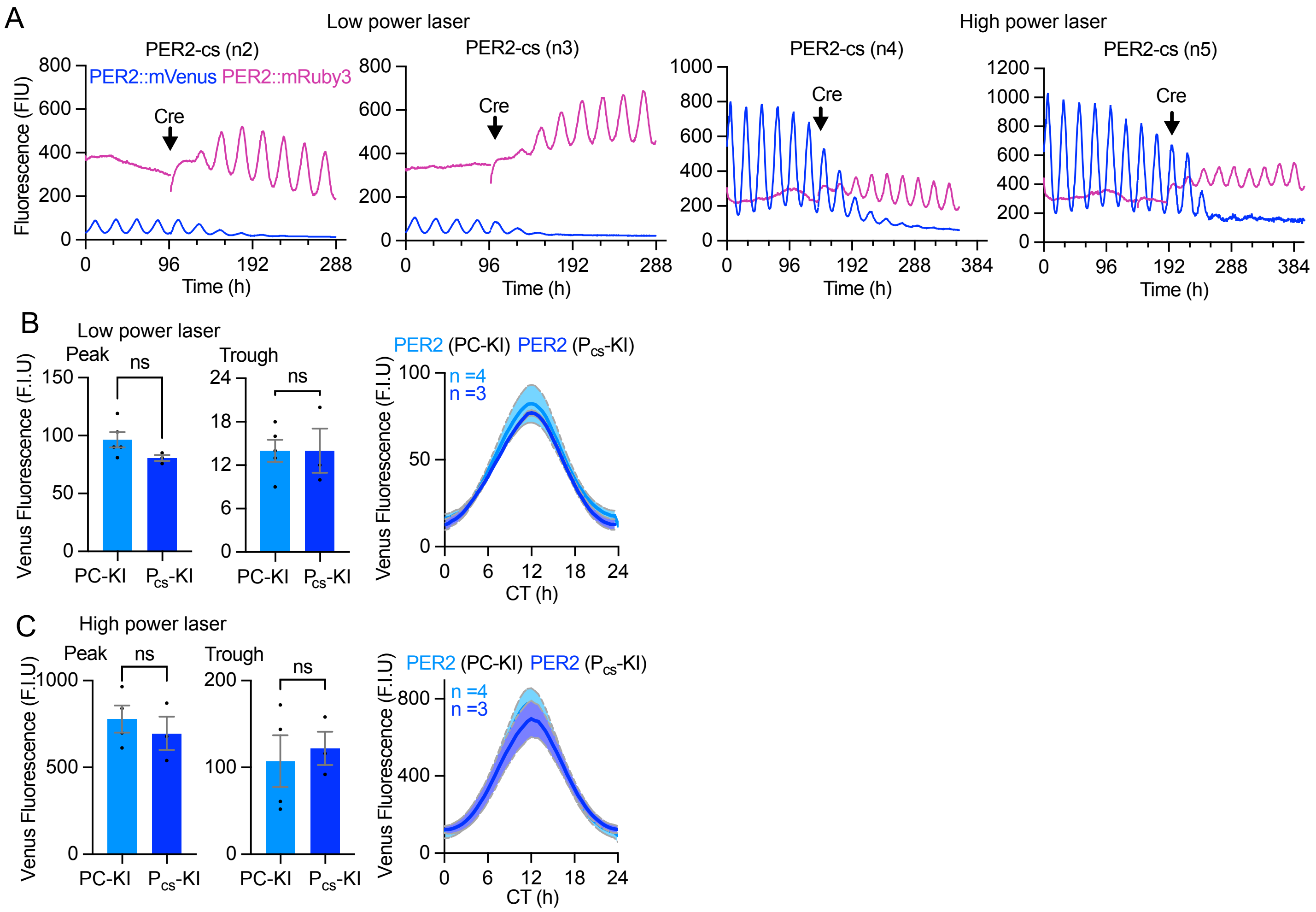

Appendix Figure S4. Cre-recombinase dependent PER2 "Colour-Switch" Venus fluorescence is comparable to the original PER2::Venus

A, Individual fluorescence traces showing the switch from PER2::Venus to PER2::mRuby3 on transduction of SCN slice with *pEf1a*-Cre AAV. Recordings were made under low-power (left) and high-power laser conditions (right), before and after, respectively, the replacement of the confocal microscope argon laser. All relative calculations between Venus and mRuby3 were made within each laser condition only. **B-C,** Group data (mean ± SEM) show that $PER2_{CS}$::Venus fluorescence is comparable to the original PER2::Venus, measured under **B,** low laser power and **C,** high laser power (*n* = 3 SCN per group). Peak (left) and trough (middle) fluorescence levels were not significantly different under either laser power condition (*n* = 3 SCN per group; t-test for each technical replicate: ns, $P > 0.05$). The average circadian profiles (right) of PER2::Venus in PC-KI SCN (light blue curve) and $PER2_{CS}$::Venus (dark blue curve) were comparable under both laser power conditions.

Appendix Figure S5

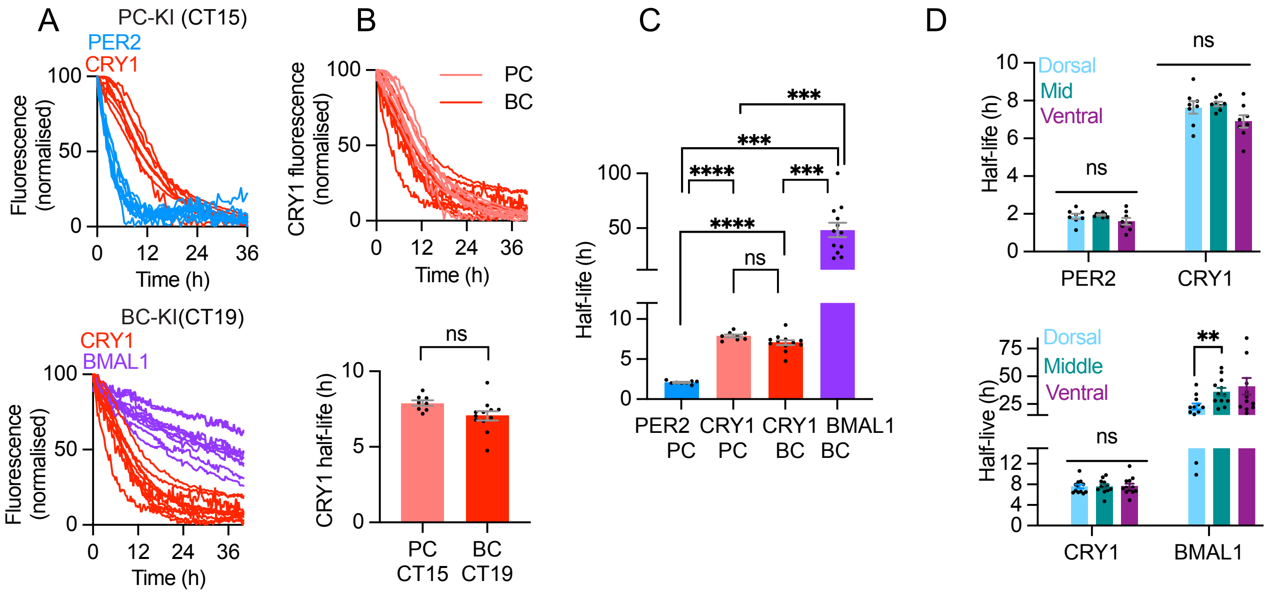

Appendix Figure S5. Half-life measures of endogenous clock proteins in the SCN determined by CHX treatment

A, Normalised fluorescence decay curves for (upper) PER2 (blue) and CRY1 (red) of individual SCN from PC-KI mice and (lower) CRY1 (red) and BMAL1 (purple) of SCN from BC-KI mice, after slices were treated with 40 μg/mL CHX at time = 0 h. **B,** (upper) CRY1 fluorescence decay curves in SCN from PC-KI (peach) and BC-KI (red) where comparable and (lower) their half-lives were not significantly different (*n* > 7 SCN per group; t-test: ns, *P* = 0.0545). **C,** Independent analyses of clock protein half-lives, across both KI mouse lines (*n* > 7 SCN; One-way ANOVA with Dunnett's T3 multiple comparisons test: ns, *P* = 0.162, ***P* = 0.0004, ****P* < 0.0001). **D,** Group data analysis of half-life across different SCN sub-regions. Within (upper) PC-KI SCN, PER2 and CRY1 did not vary across dorsal (blue), middle (teal) or ventral (purple) regions (*n* > 7 SCN, Two-way ANOVA with Tukey's multiple comparisons test: PER2: ns, *P* = 0.8571, *P* = 0.5766, *P* = 0.1830, CRY1: ns, *P* = 0.8046, *P* = 0.1052, *P* = 0.0610). Within (lower) BC-KI SCN, CRY1 did not vary across regions, but the half-life of BMAL1 did vary between dorsal and middle SCN (*n* = 12 SCN, Two-way ANOVA with Šídák's multiple comparisons test: CRY1: ns, *P* = 0.9879, *P* = 0.9752, *P* = 0.9983; BMAL1: **P* = 0.0081, ns, *P* = 0.1835, *P* = 0.8785), but there was still an overlap between the half-lives within those two regions. All group data are presented as mean ± SEM. *P* values for multiple comparisons tests are listed in the following order: dorsal-middle, dorsal-ventral, middle-ventral.
