# Supplementary figures and images for: Quantitative measures of clock protein dynamics in the mouse suprachiasmatic nucleus extends the circadian time-keeping model
Source: EMBO J. 2025 Apr 17;44(13):3614–44. doi: 10.1038/s44318-025-00426-z (PMC12218236; doi:10.1038/s44318-025-00426-z)

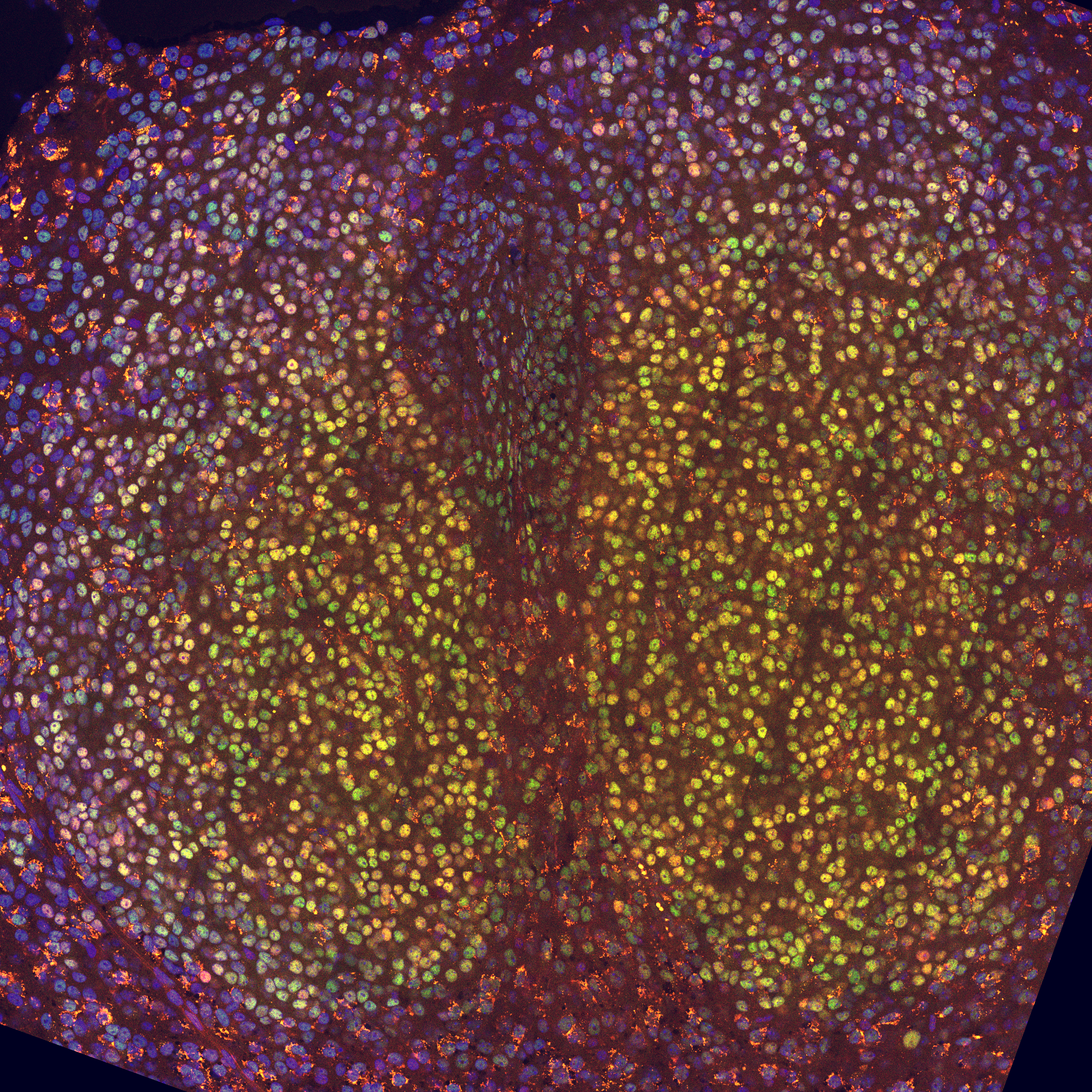

Supplement: Supplementary file 3 — Source data Fig. 1 [file 44318_2025_426_MOESM3_ESM.zip › Figure 1/1B/Figure 1B lower.tif]

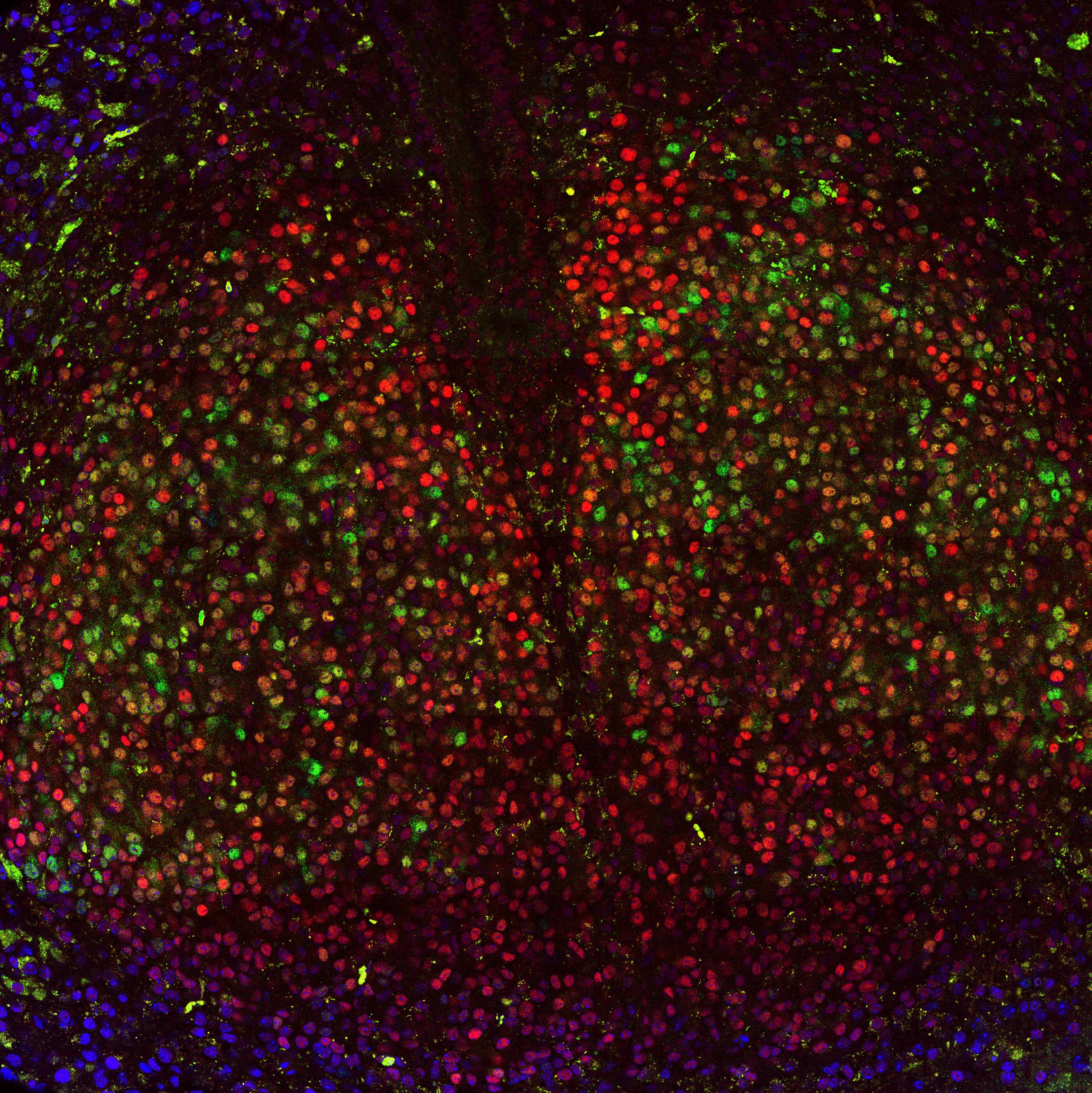

Supplement: Supplementary file 3 — Source data Fig. 1 [file 44318_2025_426_MOESM3_ESM.zip › Figure 1/1B/Figure 1B upper.tif]

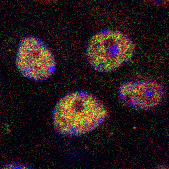

Supplement: Supplementary file 3 — Source data Fig. 1 [file 44318_2025_426_MOESM3_ESM.zip › Figure 1/1D/Figure 1D lower.tif]

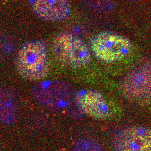

Supplement: Supplementary file 3 — Source data Fig. 1 [file 44318_2025_426_MOESM3_ESM.zip › Figure 1/1D/Figure 1D upper.tif]

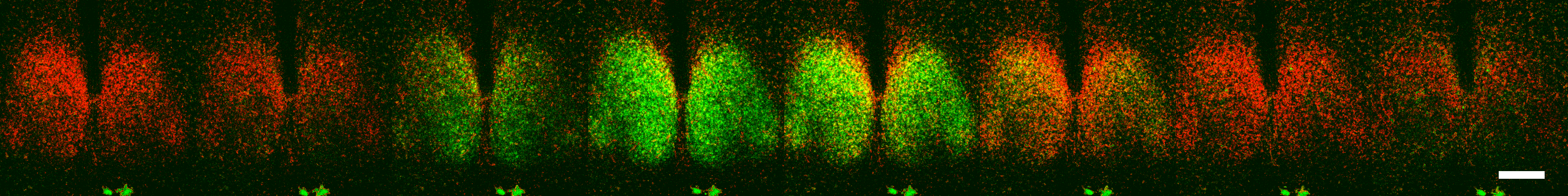

Supplement: Supplementary file 4 — Source data Fig. 2 [file 44318_2025_426_MOESM4_ESM.zip › Figure 2/2B/Figure 2B upper.tif]

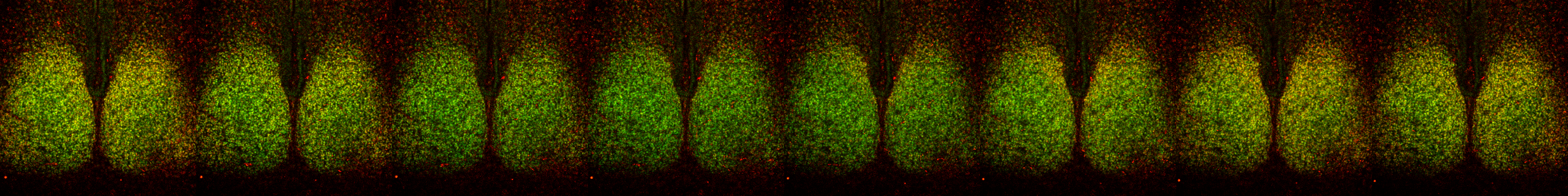

Supplement: Supplementary file 4 — Source data Fig. 2 [file 44318_2025_426_MOESM4_ESM.zip › Figure 2/2B/Figure 2B lower.tif]

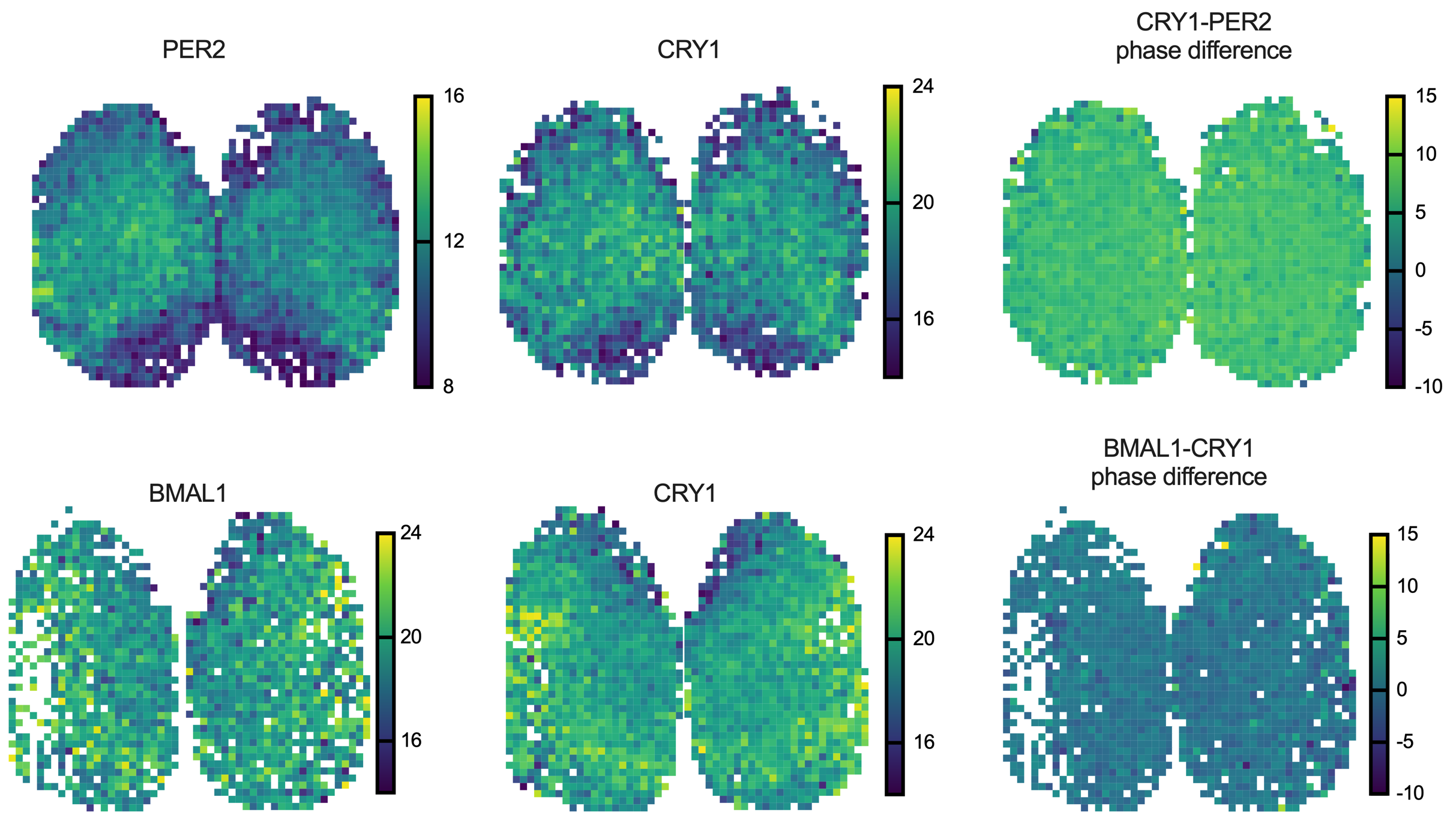

Supplement: Supplementary file 5 — Source data Fig. 3 [file 44318_2025_426_MOESM5_ESM.zip › Figure 3/3C-E/Figure 3C-3E.tiff]

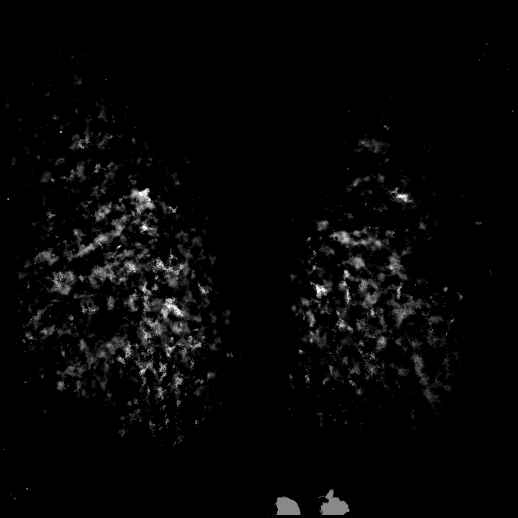

Supplement: Supplementary file 5 — Source data Fig. 3 [file 44318_2025_426_MOESM5_ESM.zip › Figure 3/3H/Figure 3H right.tif]

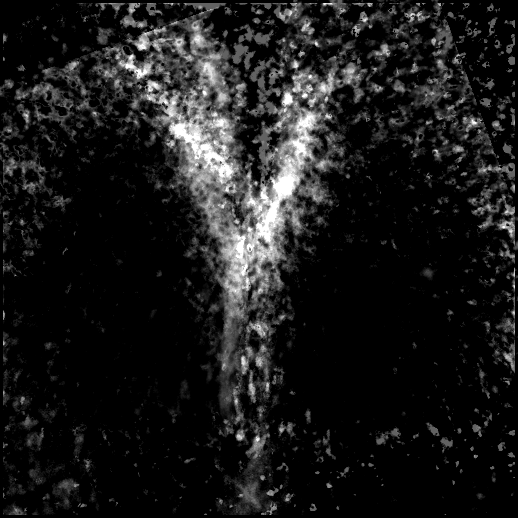

Supplement: Supplementary file 5 — Source data Fig. 3 [file 44318_2025_426_MOESM5_ESM.zip › Figure 3/3H/Figure 3H left.tif]

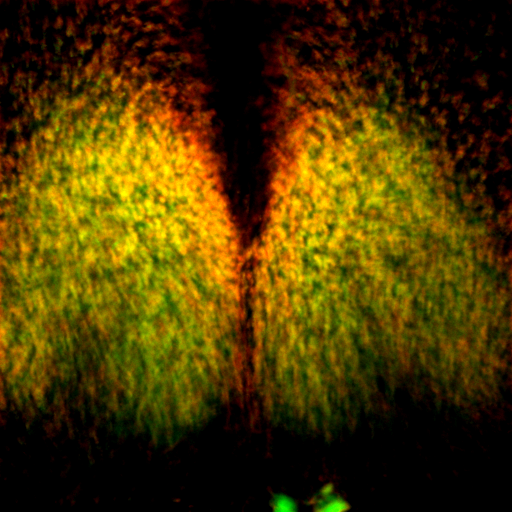

Supplement: Supplementary file 5 — Source data Fig. 3 [file 44318_2025_426_MOESM5_ESM.zip › Figure 3/3F/Figure 3F.tif]

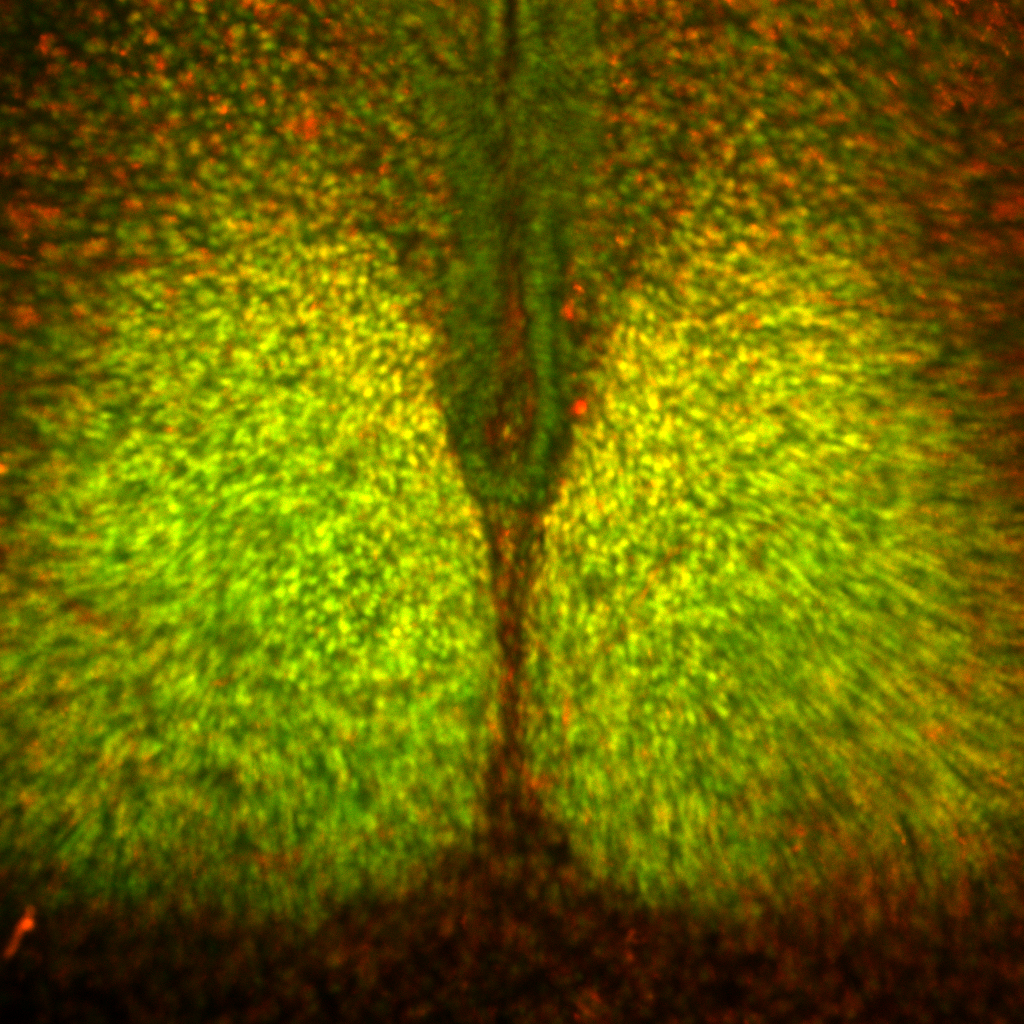

Supplement: Supplementary file 5 — Source data Fig. 3 [file 44318_2025_426_MOESM5_ESM.zip › Figure 3/3G/Figure 3G.tif]

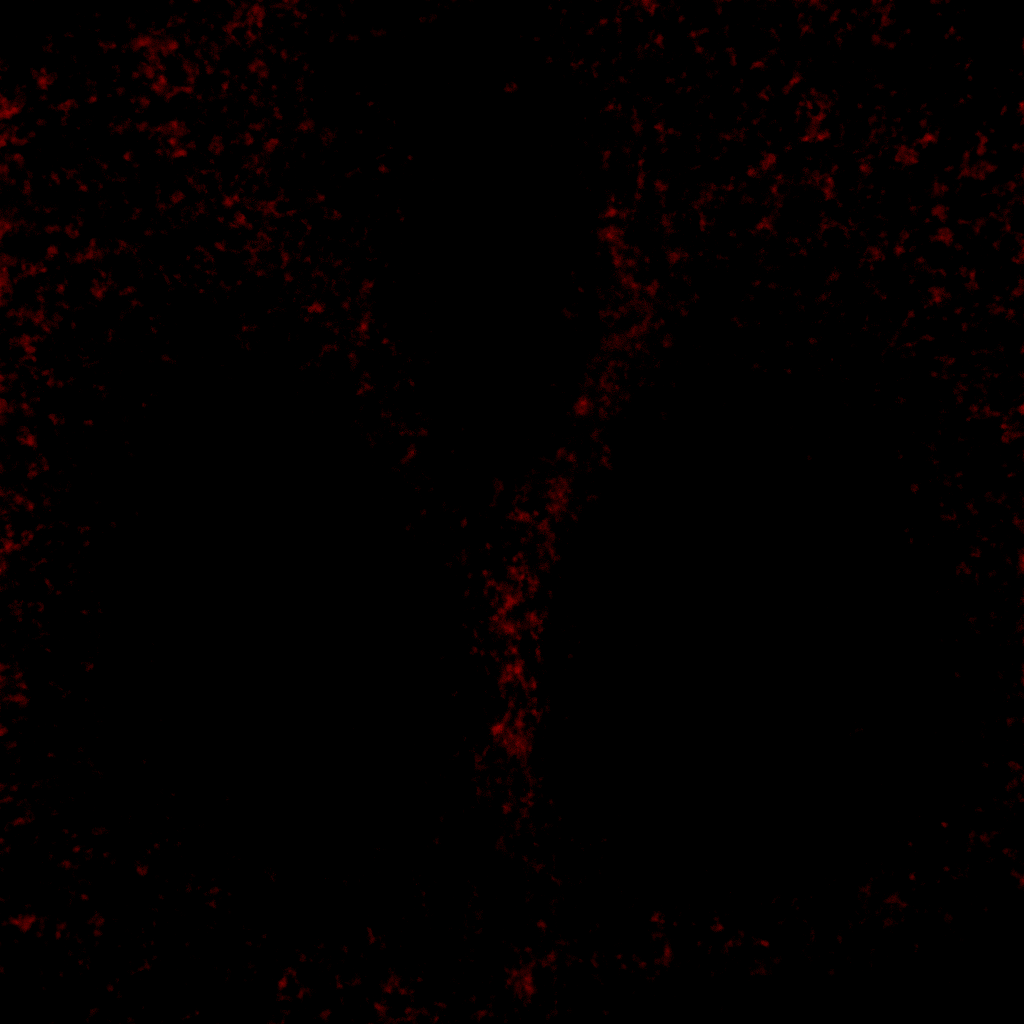

Supplement: Supplementary file 5 — Source data Fig. 3 [file 44318_2025_426_MOESM5_ESM.zip › Figure 3/3I/Figure 3I right.tif]

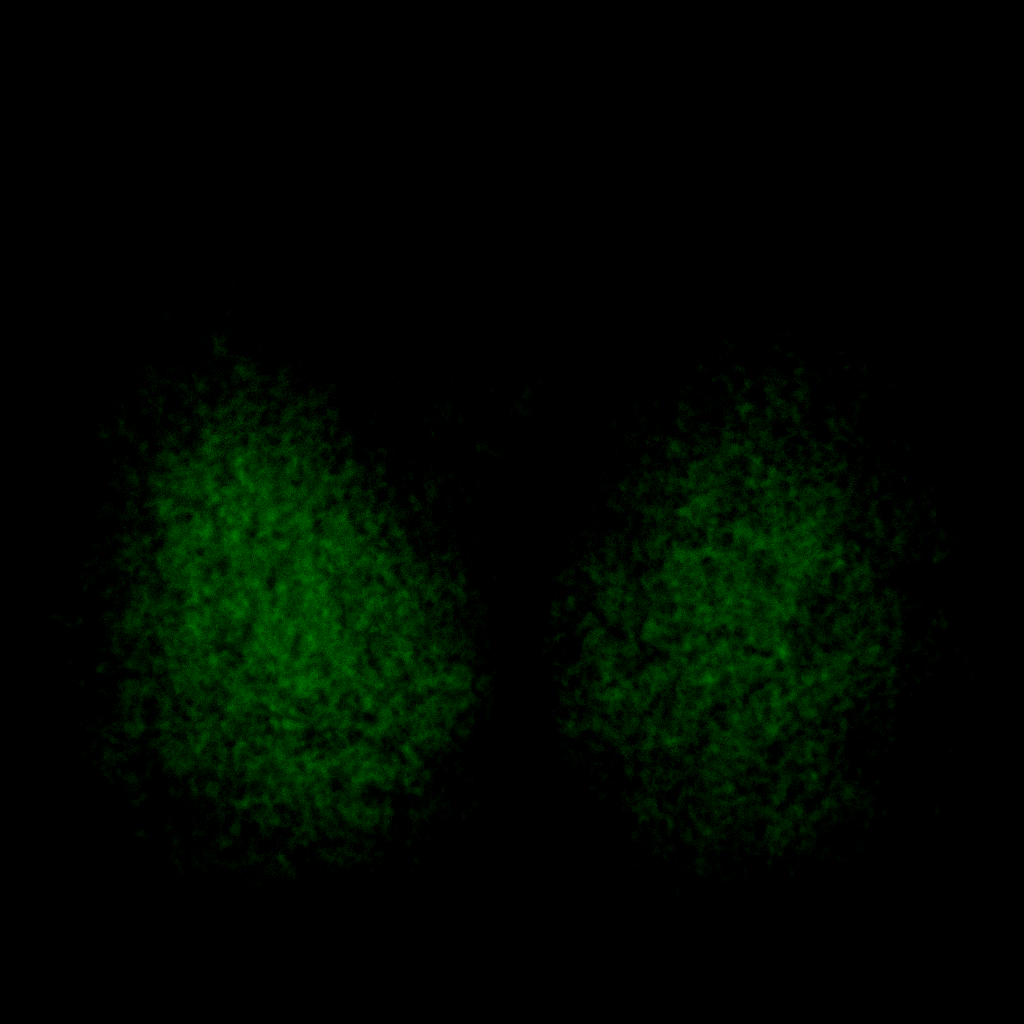

Supplement: Supplementary file 5 — Source data Fig. 3 [file 44318_2025_426_MOESM5_ESM.zip › Figure 3/3I/Figure 3I left.tif]
