# Supplementary material for: Quantitative measures of clock protein dynamics in the mouse suprachiasmatic nucleus extends the circadian time-keeping model
Source: EMBO J. 2025 Apr 17;44(13):3614–44. doi: 10.1038/s44318-025-00426-z (PMC12218236; doi:10.1038/s44318-025-00426-z)
Supplement: Supplementary file 10 — Expanded View Figures [file 44318_2025_426_MOESM10_ESM.pdf]

Expanded View Figures

Figure EV1. SCN relative molecular abundance of clock proteins measured using cre-recombinase dependent PER2 "Colour-Switch" mouse: P_{cs} KI.

(**A**) Schematic illustrates how CHX-dependent decay curves account for the background level of fluorescence in order to determine the level of fluorescence where clock protein molecules = zero (zero-point). Once the true zero-point is known, relative amplitude and baseline can be calculated, relative to the total dynamic range of fluorescence. (**B**) Schematic showing the design of the $PER2_{cs}$ KI, highlighting the locations for the PCR primers and their amplicons (upper horizontal bars; green: 5′ flanking amplicon and magenta: 3′ flanking amplicon) used for the initial identification of founder animals. An example Sanger sequence alignment (lower horizontal arrows; red) is shown for one founder animal (# 4 in PCR reactions). (**C**) 5′ (upper panel) and 3′ (lower panel) flanking PCRs identify pups, # 2, 3 and 4 as candidate knock in alleles. (**D**) Representative confocal images of an SCN slice (left, upper two rows) before transduction with *pEf1a*-Cre AAV (right, upper two rows) and 7 days after transduction, and (bottom) a merge of Venus and mRuby3 signals from before and after transduction, respectively. Scale bar = 200 µm. (**E**) Group data showing (upper) period, (lower) phase of PER2::Venus and PER2::mRuby3 oscillations from P_{cs}-KI SCN ($n = 6$; period: paired t-test: ns, $P = 0.1566$; mean phase difference: 0.31 ± 0.25 h). All group data are presented as mean ± SEM.

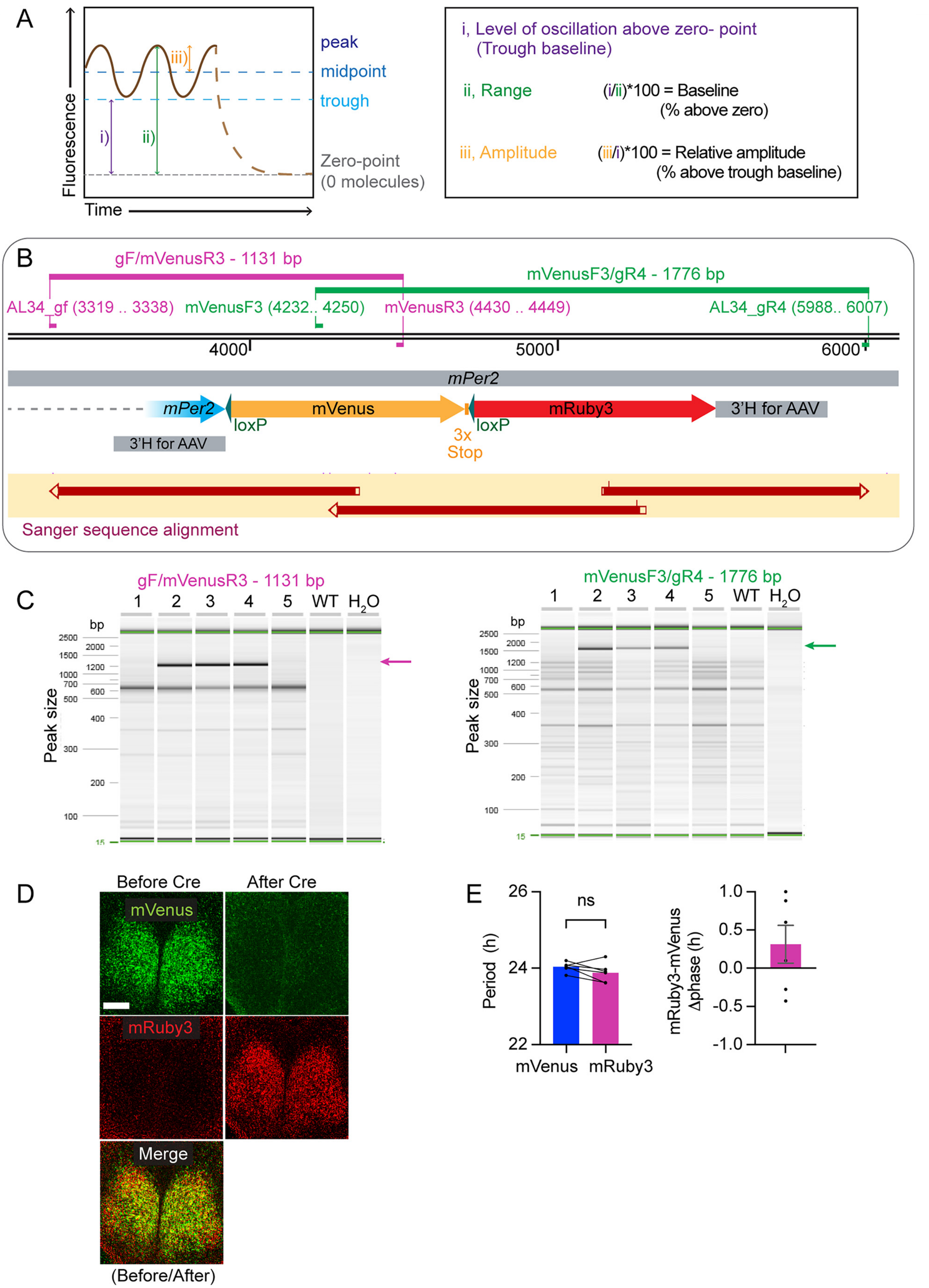
A
peak
midpoint
trough
Zero-point
(0 molecules)
Fluorescence
Time
i)
ii)
iii)
i, Level of oscillation above zero- point
(Trough baseline)
ii, Range
(i/ii)*100 = Baseline
(% above zero)
iii, Amplitude
(iii/i)*100 = Relative amplitude
(% above trough baseline)
B
gF/mVenusR3 - 1131 bp
mVenusF3/gR4 - 1776 bp
AL34_gf (3319 .. 3338)
mVenusF3 (4232.. 4250)
mVenusR3 (4430 .. 4449)
AL34_gR4 (5988.. 6007)
4000
5000
6000
mPer2
mPer2
loxP
mVenus
3x
Stop
loxP
mRuby3
3'H for AAV
3'H for AAV
Sanger sequence alignment
C
gF/mVenusR3 - 1131 bp
1 2 3 4 5 WT H2O
bp
Peak size
mVenusF3/gR4 - 1776 bp
1 2 3 4 5 WT H2O
D
Before Cre
After Cre
mVenus
mRuby3
Merge
(Before/After)
E
ns
Period (h)
mVenus
mRuby3
mRuby3-mVenus
Δphase (h)

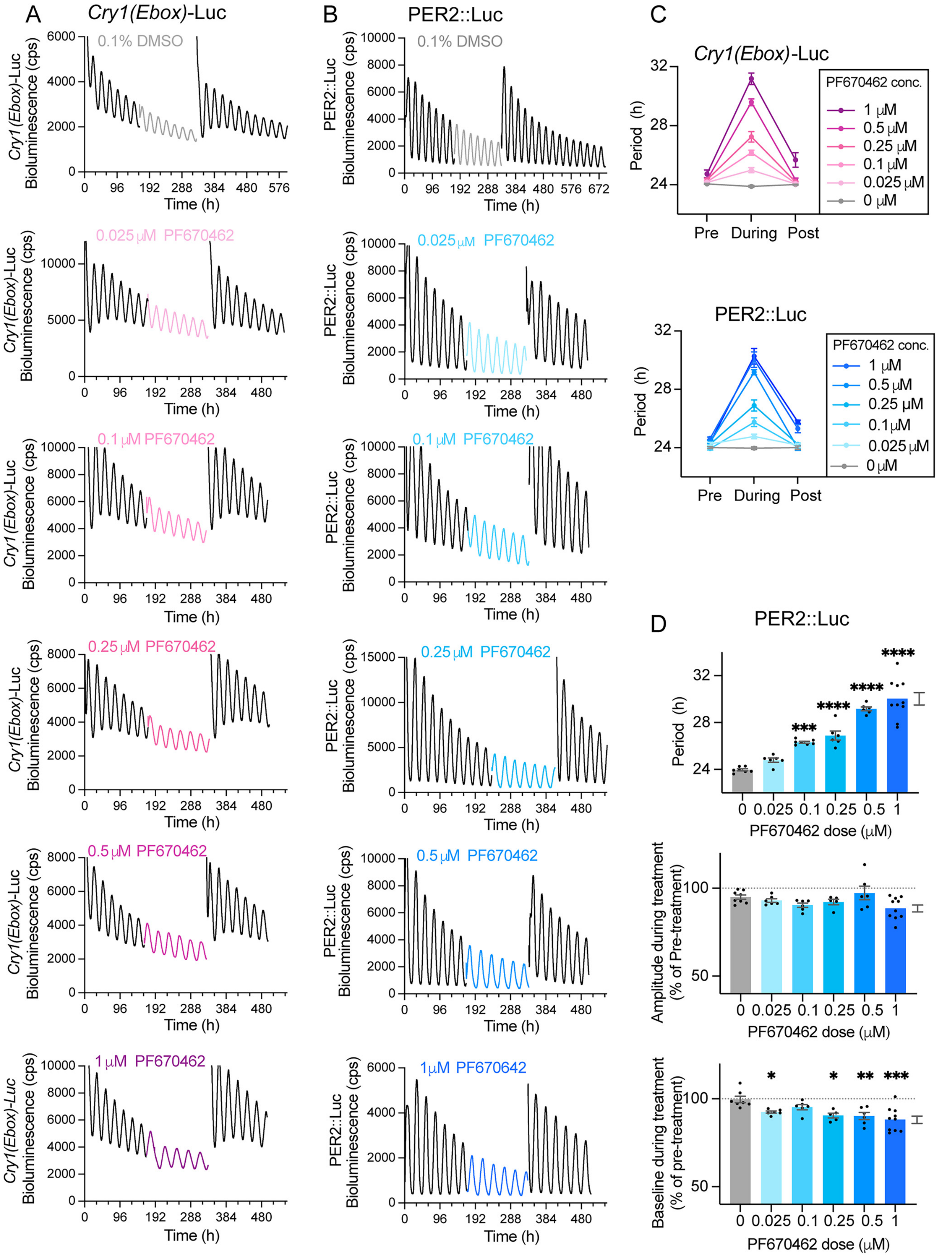

A
Cry1(Ebox)-Luc
0.1% DMSO
0.025 μM PF670462
0.1 μM PF670462
0.25 μM PF670462
0.5 μM PF670462
1 μM PF670462
Cry1(Ebox)-Luc Bioluminescence (cps)
Time (h)
B
PER2::Luc
0.1% DMSO
0.025 μM PF670462
0.1 μM PF670462
0.25 μM PF670462
0.5 μM PF670462
1μM PF670642
PER2::Luc Bioluminescence (cps)
Time (h)
C
Cry1(Ebox)-Luc
PER2::Luc
PF670462 conc.
1 μM
0.5 μM
0.25 μM
0.1 μM
0.025 μM
0 μM
Period (h)
Pre
During
Post
D
PER2::Luc
Period (h)
Amplitude during treatment (% of Pre-treatment)
Baseline during treatment (% of pre-treatment)
PF670462 dose (μM)

Figure EV2. The effect of stabilisation of PER on TTFL rhythms.

(**A**, **B**) Representative bioluminescence traces show TTFL reporters *Cry1(Ebox)*-Luc (transcriptional) and PER2::Luc (translational) in SCN slices treated with different doses of PER stabiliser drug, PF670462. Before treatment and wash-out after treatment traces are shown in black. Treatment traces are shown in pink for *Cry1(Ebox)*-Luc and blue for PER2::Luc SCN treated with PF670462. Vehicle traces are shown in grey for both reporters. (**C**) Circadian period of (upper) *Cry1*(*Ebox*)-Luc or (lower) PER2::Luc oscillations before (pre), during and after wash-out (post) of different concentrations PF670462. This shows dose dependence and reversibility of effect on circadian period. (**D**) PF670462 dose dependently (upper) lengthens period (ns, $P = 0.4042$, ***$P = 0.0002$, ****$P < 0.0001$) and (lower) minimally drops circadian baseline (ns, $P = 0.3470$, *$P = 0.0108$, **$P = 0.0052$, ***$P = 0.0001$) but does not alter circadian amplitude (ns, $P = 0.9478$, $P = 0.3996$, $P = 0.8470$, $P = 0.8915$, $P = 0.0747$) of PER2::Luc oscillations. All group data are presented as mean ± SEM and data in (**D**) ($n > 5$ SCN per dose group) are analysed with One-way ANOVAs with Dunnett's multiple comparisons tests.

© The Author(s)

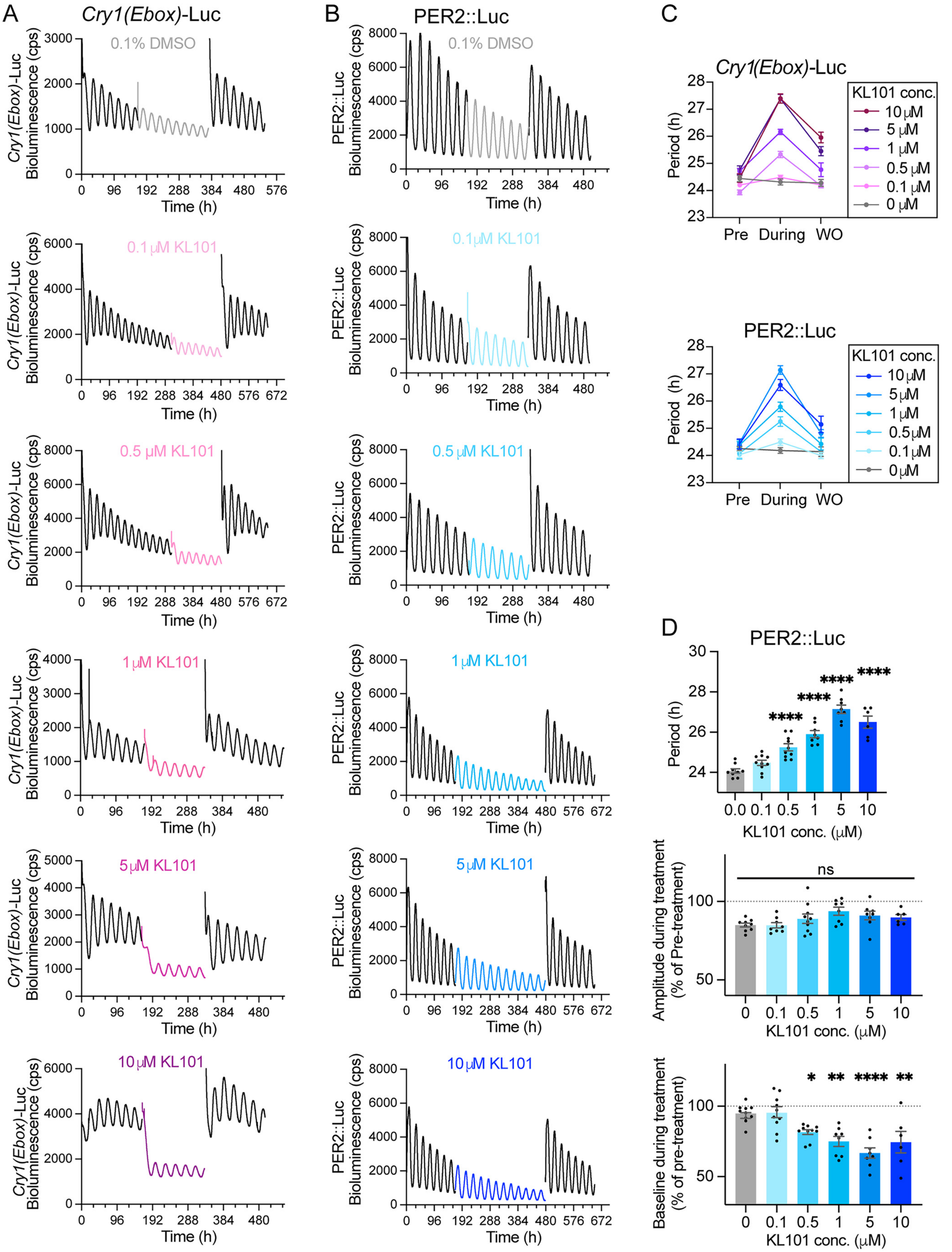
A
Cry1(Ebox)-Luc
B
PER2::Luc
C
D
0.1% DMSO
0.1 μM KL101
0.5 μM KL101
1 μM KL101
5 μM KL101
10 μM KL101
Cry1(Ebox)-Luc Bioluminescence (cps)
PER2::Luc Bioluminescence (cps)
Time (h)
KL101 conc.
Period (h)
Pre During WO
KL101 conc. (μM)
Amplitude during treatment (% of Pre-treatment)
Baseline during treatment (% of pre-treatment)
ns

Figure EV3. The effect of stabilisation of CRY1 on TTFL rhythms.

(**A**, **B**) Representative bioluminescence traces show TTFL reporters *Cry1(Ebox)*-Luc (transcriptional) and PER2::Luc (translational) in SCN slices treated with different doses of CRY1 stabiliser drug, KL101. Before treatment and wash-out after treatment traces are shown in black. Treatment traces are shown in pink for *Cry1(Ebox)*-Luc and blue for PER2::Luc SCN treated with KL101. Vehicle traces are shown in grey for both reporters. (**C**) Circadian period of (left) *Cry1*(*Ebox*)-Luc or (right) PER2::Luc oscillations before (pre), during and after wash-out (post) of different concentrations KL101 shows dose dependence and reversibility of effect on circadian period. (**D**) KL101 dose dependently (upper) lengthens period (ns, $P = 0.3274$, ****$P < 0.0001$), does not alter circadian amplitude (ns, $P > 0.9999$, $P = 0.5658$, $P = 0.2398$, $P = 0.5226$) but does (lower) drops circadian baseline ($P > 0.9999$, *$P = 0.0345$, **$P < 0.0028$, ****$P < 0.0001$) of PER2::Luc oscillations. All group data are presented as mean ± SEM and data in (**D**) ($n > 5$ SCN per group) are analysed with One-way ANOVAs with Dunnett's multiple comparisons tests.

© The Author(s)

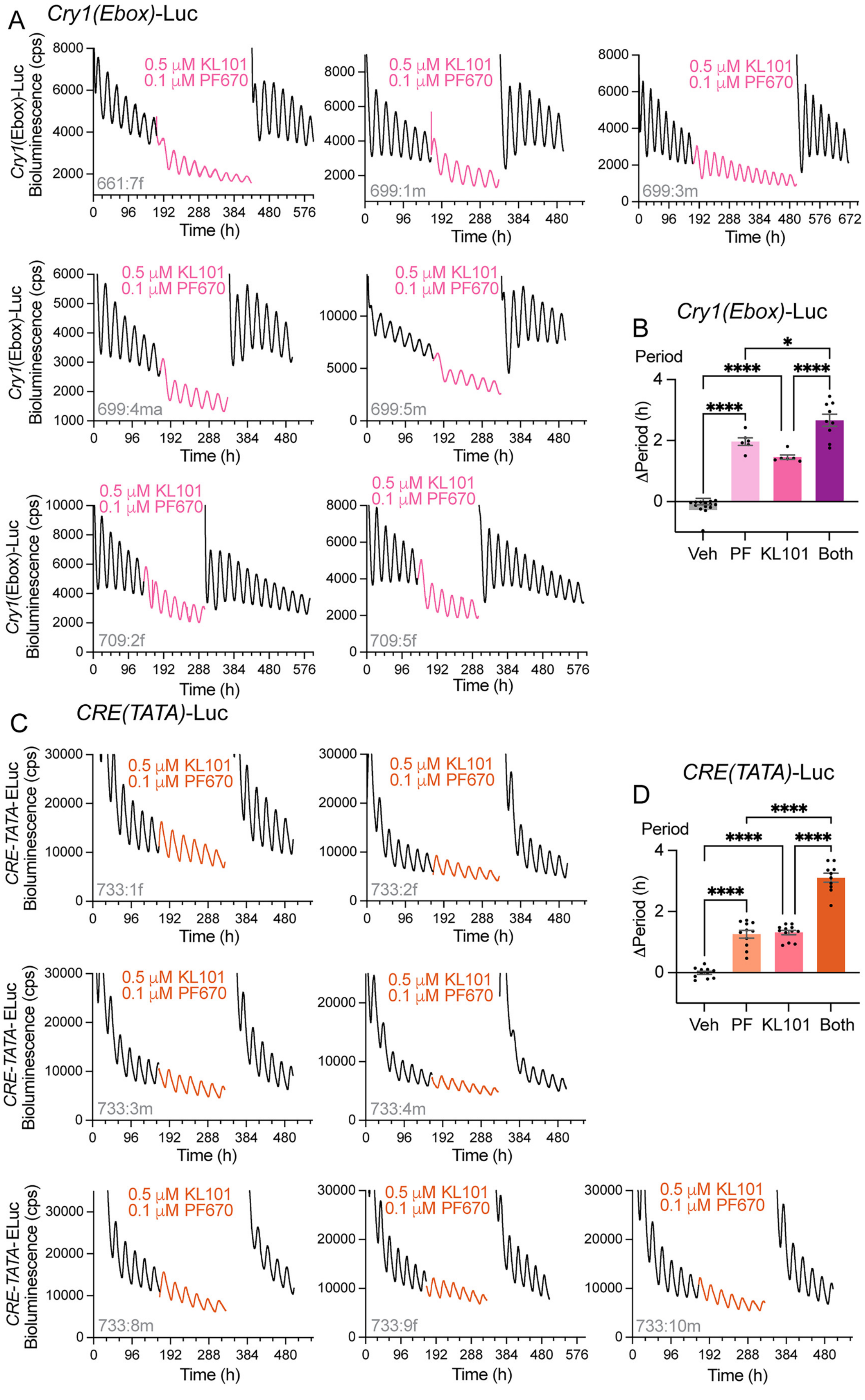
A
Cry1(Ebox)-Luc
Cry1(Ebox)-Luc Bioluminescence (cps)
0.5 µM KL101
0.1 µM PF670
661:7f
699:1m
699:3m
699:4ma
699:5m
709:2f
709:5f
Time (h)
B
Cry1(Ebox)-Luc
Period
ΔPeriod (h)
Veh
PF
KL101
Both
C
CRE(TATA)-Luc
CRE-TATA-ELuc Bioluminescence (cps)
733:1f
733:2f
733:3m
733:4m
733:8m
733:9f
733:10m
D
CRE(TATA)-Luc

Figure EV4. Combined stabilisation of PER and of CRY1 have an additive effect on the SCN circadian clock.

(**A**) Representative bioluminescence recordings of *Cry1*(*Ebox*)-Luc intra-TTFL transcriptional oscillations in SCN slices before (black trace) and during double-drug-treated with 0.1 μM PF670462 and 0.5 μM KL101 (pink trace) and after wash-out (2nd black trace). (**B**) Group data show circadian period change (Δ) of *Cry1*(*Ebox*)-Luc oscillations, after drug treatment compared to pre-treatment, where there was an additive effect of double-drug treatment compared with single drug treatment (*$P = 0.0190$, ****$P < 0.0001$). (**C**) As in (**A**), but for *CRE-TATA*-Luc extra-TTFL transcriptional oscillations (treatment trace in dark orange). (**D**) as in (**B**), but for *CRE-TATA*-Luc oscillations. (****$P < 0.0001$). All group data ($n > 5$ SCN per group) are presented as mean ± SEM and are analysed with One-way ANOVAs with Tukey's multiple comparisons tests.

 © The Author(s)

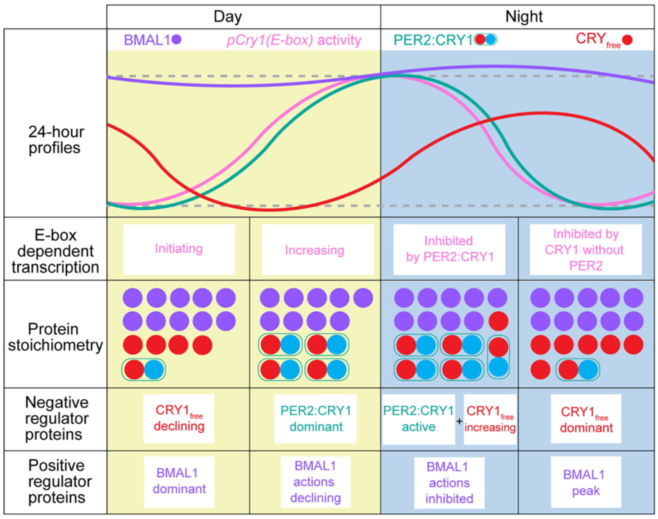

Figure EV5. Schematic of TTFL protein and E-box activity in the SCN.

Schematic shows the circadian dynamics of different TTFL activities. 1st row: plots of oscillations of (purple) BMAL1, (pink) *pCry1(Ebox)* promoter activity, (teal) PER2:CRY1 heteromeric complex and (red) free CRY1 outside of the PER2:CRY1 heteromeric complex. The minimum and maximum levels of E-box activation, suggested to be set by PER2 and $CRY1_{free}$, respectively, are indicated with dotted grey lines. 2nd row: descriptions of E-box-dependent transcriptional activities, relating to the oscillation of *pCry1* promoter-activity. 3rd row: graphic depicting estimated and inferred integer stoichiometries of clock proteins. See main text and methods to see full description about assumptions made regarding calculations of PER2:CRY1 complexes. 4th row: status of negative regulator protein/ repressor complex activities. 5th row: status of positive regulator (BMAL1) activities. Rows 1–4 are divided into quarters of the circadian cycle: Early morning, late morning, early night and late night. The transition between day and night is CT12.
