# Supplementary material for: Quantitative measures of clock protein dynamics in the mouse suprachiasmatic nucleus extends the circadian time-keeping model
Source: EMBO J. 2025 Apr 17;44(13):3614–44. doi: 10.1038/s44318-025-00426-z (PMC12218236; doi:10.1038/s44318-025-00426-z)
Supplement: Supplementary file 2 — Peer Review File [file 44318_2025_426_MOESM2_ESM.pdf]

THE EMBO JOURNAL

Quantitative measures of clock protein dynamics in the mouse suprachiasmatic nucleus extends the circadian time-keeping model

Nicola Smyllie, Alexander Koch, Antony Adamson, Andrew Patton, Adam Johnson, James Bagnall, Olivia Johnson, Qing-Jun Meng, Andrew S.I. Loudon, and Michael Hastings

Corresponding author(s): Michael Hastings (mha@mrc-lmb.cam.ac.uk) , Nicola Smyllie (nsmyllie@mrc-lmb.cam.ac.uk)

Review Timeline:

Submission Date:	25th Nov 24
Editorial Decision:	8th Jan 25
Revision Received:	4th Feb 25
Editorial Decision:	20th Feb 25
Revision Received:	26th Feb 25
Accepted:	3rd Mar 25

Editor: Ioannis Papaioannou

Transaction Report:

(Note: With the exception of the correction of typographical or spelling errors that could be a source of ambiguity, letters and reports are not edited. Depending on transfer agreements, referee reports obtained elsewhere may or may not be included in this compilation. Referee reports are anonymous unless the Referee chooses to sign their reports.)

1st Editorial Decision **8th Jan 2025**

Dear Dr. Hastings,

Thank you for submitting your manuscript EMBOJ-2024-119731-T for consideration by The EMBO Journal, and for your patience during peer review. Your manuscript has now been seen by three experts in the field, and we have received the full set of their thorough reports, which are included below. Please note that the report of referee #2 contains figures, and is therefore attached as a pdf file.

As you will see, all three referees recognize that the work is intriguing and significant, the state-of-the-art experimental approaches and the quality of the data commendable, and the manuscript well-written. They are all very supportive of the manuscript and raise no major concerns, and they provide a few constructive and reasonable suggestions for further strengthening of the manuscript without the need to perform additional experiments. We agree with the referees that the work and the manuscript would benefit from the suggested additions and improvements.

Given the referees' positive comments and recommendations, I would like to invite you to submit a revised version of the manuscript along with a detailed point-by-point response addressing all referees' comments. I should add that it is The EMBO Journal policy to allow only a single round of major revision, and acceptance of your manuscript will therefore depend on the completeness of your responses in this revised version. Please let me know if you have any questions or comments that you would like to discuss with me.

We generally allow three months as standard revision time (April 7, 2025). As a matter of policy, competing manuscripts published during this period will not negatively impact our assessment of the conceptual advance presented by your study. However, we request that you contact us as soon as possible upon publication of any related work, to discuss how to proceed. Should you foresee a problem in meeting this three-month deadline, please let us know in advance and we may be able to grant an extension.

Thank you for the opportunity to consider your work for publication in The EMBO Journal. I look forward to your revision.

Best regards,

Ioannis

Ioannis Papaioannou, PhD
Editor, The EMBO Journal
i.papaioannou@embojournal.org

*****
Instructions for preparing your revised manuscript
*****

1. When you are ready to submit the revision, please upload:

- A Word file of the manuscript text (including legends of main Figures, EV Figures and Tables). Please make sure that changes are highlighted (or "tracked") to be clearly visible.

- Individual production-quality figure files (one file per figure). When assembling your figures, please refer to our figure preparation guidelines in order to ensure proper formatting and readability in print as well as on screen:
https://bit.ly/EMBOPressFigurePreparationGuideline
If the data shown in a figure are obtained from n {less than or equal to} 2, please use scatter plots showing the individual data points.
Please also include scale bars in all microscopy images.
The following points must be specified in each figure legend:
i. the name of the statistical test used to generate error bars and P values
ii. the number (n) of independent experiments (please specify technical or biological replicates) underlying each data point (discussion of statistical methodology can be reported in the Materials and Methods section, but figure legends should contain a basic description of n, P, and the test applied)
iii. the nature of the bars and error bars (s.d., s.e.m.).
See also guidelines for figure legends: https://www.embopress.org/page/journal/14602075/authorguide#figureformat

- A point-by-point response to the referees' comments, with a detailed description of the changes made (as a word file). All

referees' concerns must be fully addressed and their suggestions taken on board. When preparing your letter of response to the referees' comments, please bear in mind that this will form part of the Review Process File and will therefore be available online to the community. Please note that you have the possibility to opt out of the transparent process at any stage prior to publication by letting the editorial office know (contact@embojournal.org); if you do opt out, the Review Process File link will point to the following statement: "No Review Process File is available with this article, as the authors have chosen not to make the review process public in this case.". For more details on our Transparent Editorial Process, please visit our website: https://www.embopress.org/page/journal/14602075/authorguide#transparentprocess

- Expanded View (EV) files (replacing Supplementary Information) that are collapsible/expandable online. A maximum of 5 EV Figures can be typeset. EV Figures should be cited as "Figure EV1, Figure EV2" etc. in the text, and their respective legends should be included in the manuscript file after the legends of regular figures. See detailed instructions regarding Expanded View files here:
https://www.embopress.org/page/journal/14602075/authorguide#expandedview

- For the figures that you do NOT wish to display as Expanded View figures, they should be bundled together with their legends in a single PDF file called "Appendix", which should start with a short Table of Contents (including page numbers). Appendix figures should be referred to in the main text as: "Appendix Figure S1, Appendix Figure S2" etc. Please see detailed instructions here: https://www.embopress.org/page/journal/14602075/authorguide#expandedview

- A complete author checklist, which you can download from our author guidelines (https://www.embopress.org/page/journal/14602075/authorguide). Please note that the checklist will also be part of the Review Process File.

2. Please note that no statistics should be calculated and shown in Figures if n=2. Please also note that each p value should be reported as an exact value.

3. Before submitting your revision, primary datasets (and computer code, where appropriate) produced in this study need to be deposited in appropriate public databases (see https://www.embopress.org/page/journal/14602075/authorguide#dataavailability).

The accession numbers, database, and the specific URLs (links) should be listed in a formal "Data availability" section (placed after Methods), following the example below:

"The RNA-seq datasets produced in this study are available in the following database:
Gene Expression Omnibus GSE46843 (https://www.ncbi.nlm.nih.gov/geo/query/acc.cgi?acc=GSE46843)"

*** All links should resolve to a page where the data can be accessed. ***

*** Please remember to provide in the Data availability section of your revised manuscript reviewer passwords if the datasets are not yet public. ***

*** The Data Availability Section is restricted to new primary data that are part of this study. In case you have no data that require deposition in a public database, please state so instead of referring to the database: "Our study includes no data deposited in public repositories." under the heading "Data availability". ***

4. Please check that the title and the abstract of the manuscript are brief, yet explicit, even to non-specialists. The length of the title should not exceed 100 characters, and the abstract should be a single paragraph not exceeding 175 words.

5. Please also note our reference format: https://www.embopress.org/page/journal/14602075/authorguide#referencesformat.

6. At EMBO Press we ask authors to provide source data for the main manuscript figures. Our source data coordinator will contact you to discuss which figure panels we would need source data for and will also provide you with helpful tips on how to upload and organize the files.

7. Please remember: digital image enhancement is acceptable practice, as long as it accurately represents the original data and conforms to community standards. If a figure has been subjected to significant electronic manipulation, this must be noted in the figure legend or in the "Materials and Methods" section. The editors reserve the right to request original versions of figures and the original images that were used to assemble the figure.

8. Our journal encourages inclusion of data citations in the reference list to directly cite datasets that were obtained from public databases. Data citations in the article text are distinct from normal bibliographical citations and should directly link to the database records from which the data can be accessed. In the main text, data citations are formatted as follows: "Data ref: Smith et al, 2001" or "Data ref: NCBI Sequence Read Archive PRJNA342805, 2017". In the Reference list, data citations must be labeled with "[DATASET]". A data reference must provide the database name, accession number/identifiers, and a resolvable link to the landing page from which the data can be accessed at the end of the reference. Further instructions are available at:

https://www.embopress.org/page/journal/14602075/authorguide#referencesformat.

9. We request authors to consider both actual and perceived competing interests. Please review our policy (https://www.embopress.org/page/journal/14602075/authorguide#conflictsofinterest) and update your competing interests statement if necessary. Please name this section 'Disclosure and competing interests statement' and place it after the Acknowledgements section.

10. Please note that all corresponding authors are required to provide an ORCID ID upon submission of a revised manuscript (https://orcid.org/). Please find instructions on how to link your ORCID ID to your account in our manuscript tracking system in our Author guidelines (https://www.embopress.org/page/journal/14602075/authorguide#authorshipguidelines).

11. We use CRediT to specify the contributions of each author in the journal submission system. CRediT replaces the author contribution section, which should be removed from the manuscript. Please use the free text box to provide more detailed descriptions. See also guide to authors: https://www.embopress.org/page/journal/14602075/authorguide#authorshipguidelines.

12. Further information is available in our Guide For Authors: https://www.embopress.org/page/journal/14602075/authorguide

13. We would also welcome the submission of cover suggestions or motifs to be used by our Graphics Illustrator in designing a cover.

14. Please use the link below to submit your revision:
https://emboj.msubmit.net/cgi-bin/main.plex

Referee #1:

This reviewer carefully read the manuscript "Behavior of PER2 and CRY1 in the suprachiasmatic nucleus contradicts canonical circadian clock model" by the authors.
Using real-time fluorescence imaging, the authors studied the dynamics of three key circadian clock proteins (PER2, CRY1, and BMAL1) in SCN slices. The study revealed that PER2 and CRY1 proteins operate independently within the circadian clock mechanism, challenging the traditional model where they were thought to function together in the same place as a complex. These findings provide new insights into the complexity of circadian rhythm regulation and suggest that the activator and repressor components of the circadian clock are not static entities but involve complex spatiotemporal relationships. The manuscript is easy to read and has clear conclusions with plentiful experiments. The reviewer considers that this study presents significant findings on clock gene TTFL in the SCN using well-defined and high-qualified approaches. The reviewer has suggested some points to be improved.

#Minor comments
For readers who are not familiar with this field, the reviewer recommends providing a more detailed explanation of why the present experimental results differ from previous studies about circadian clocks in the SCN. Live imaging of clock proteins has been demonstrated in previous studies, such as "Real-Time Recording of Circadian Per1 and Per2 Expression in the Suprachiasmatic Nucleus of Freely Moving Rats. Yamaguchi Y et al. J Biol Rhythms. 2016 Feb;31(1):108-11." Would it be possible to discuss in detail the distinctive features and advantages of the present study's methodology compared to previous works?

Figure 5i and j, 6i and j. In the treatment by PF670462 and KL 101, the dose-dependent decrease in Amplitude and Baseline are observed in Figure 5i and j, 6i and j. The reviewer considers that the cytotoxic effects of the stabilizer drugs might cause the decrease. Would it be possible to rule out these concerns based on previous literature and available data?

Figure 1 d. The reviewer recommends explaining what the blue color represents in Merge Figures.

Figure 3 c. To assist readers who may not be familiar with SCN anatomy, the reviewer suggests including anatomical orientation markers in the figure. Specifically, it is helpful to have directional indicators that clearly show which side is dorsal or ventral.

Referee #2:

Smyllie et al:

Behavior of PER2 and CRY1 in the suprachiasmatic nucleus contradicts canonical circadian clock model

Sometimes you get manuscripts for review that are crammed full of data and are very tiring to read. After working through such papers, one often has the feeling that the insights gained do not justify the effort of reading them.

This manuscript, however, stands apart. While it is complex and data-rich, it is highly stimulating and undoubtedly represents a significant and important contribution to the field. The data are state-of-the-art, featuring recordings for SCN slices from two crossed KI mice expressing pairs of endogenously tagged clock proteins, PER2/CRY1 and BMAL1/CRY1, respectively. A highlight of the study is undoubtedly the PER2 color-switch KI, which enables precise quantification of the expression ratios of BMAL1, CRY1, and PER2.

I therefore do not request additional experiments, as they could not improve the quality of the paper. Such requests would result in a different, bu
t not necessarily better, paper and would unnecessari
ly consume time and resources that the lab could more effectively dedica
te to new research.

Yet I have some comment and suggestions:

Minor points:
In the final paragraph of the Introduction, PER and CRY should be specified as PER2 and CRY1, right?

In Fig 4A right panel: the BMAL1 + CHX trace does not approach the dotted baseline. Rather, by extrapolation, the BMAL data seems to cross the dotted li
ne.

The authors show in Figures 5 - 7 that inhibitors, PF670 and KL101, lengthened the descending phase of their targets, PERs and CRY1, respectively but stretched the rhythms of the corresponding other partner in a symmetrical manner. Application of both inhibitors lengthened period in an additive manner. The authors suggest that actions of increased PER and CRY1 stabili
ty are independent. I totally agree with this interpretation. However, as sta
tement in the paper this could potentially lead to some confusion. We know that PERs do not repress without CRYs. I guess what the authors mean is tha
t the action of the PER:CRY complex is governed by PER stabili
ty and not by CRY,

which is protected from degradation when bound to PERs, while KL101 acts only on free CRY.

Suggestions and comments:

I believe the authors may be overly critical in dismissing the "old clock model". It is well established that PER proteins are essential for rhythmicity and cannot function without CRYs. Structural studies have provided detailed insights into how these proteins interact, and we know that disrupting these interactions impacts the clock. Therefore, both PER:CRY complexes and CRY alone are capable of interacting with the BMAL1:CLOCK complex, and I am sure the authors fully agree with this.
However, what this manuscript shows in an impressive and convincing manner is that the repression cannot be mediated by a simple stochiometric mechanism where early repression is mediated by a stoichiometic PER:CRY-BMAL1:CLOCK complex, which is then followed by late repression via a stoichiometic CRY1-BMAL1:CLOCK complex and finally, after CRY1 degradation, by a transcriptionally active BMAL1:CLOCK complex. Rather, free CRY1 or CRY1:PER complexes are available throughout the circadian day. Hence, the prevalent dual mode repression model must be substantially modified, taking into account the quantitative data presented here.
Yet, the authors might consider revising their title to better reflect this updated perspective by softening the statement. A possible alternative could be: "Spatial and Temporal Dynamics and Abundance of Core Clock Proteins in the SCN Prompt a Re-Evaluation (or Adjustment) of TTFL Models, or something along this line

Finally, since the manuscript was so stimulating, I would like to share some thoughts and data that I looked up while reviewing the manuscript, which the authors may consider incorporating into their discussion, if appropriate.

1) I believe our field still adheres too strongly to an outdated, stoichiometry-based model of transcription regulation, where, in its simplest form, a single transcription factor bound to a specific recognition site interacts with one corepressor or activator. The current understanding seems to be shifting toward a model where highly dynamic hubs of transcription factors transiently assemble and accumulate at the promoter. These hubs recruit coactivators and/or repressors up to functionally relevant local concentrations but do not necessarily adhere to a fixed stoichiometry. The dwell time of such TF hubs

could be relatively short (seconds to minutes), making them challenging to detect via microscopy.

An important aspect of transcription regulation likely involves modulation of the poised state (referred to by us as the 'late repressive complex'), where Pol II constantly initiates and prematurely terminates transcription (see Pol II S5 ChIP-seq data in Koike et al.). Additionally, the Koike ChIP-seq data demonstrate the presence of CRY1, BMAL1, and Pol II-S5 at the promoter during the poised state. Recruitment of Pol II would not be consistent with a model in which CRY1 quantitatively sequesters the BMAL1 TAD. Since either CRY1 or CRY1:PERs are present throughout the day, promoters are likely never fully active. The ratio of CRY1 to BMAL1:CLOCK within a TF hub at the promoter could determine the degree of repression (always high), without relying on a strict 1:1 stoichiometry. Rhythmically accumulating PER:CRY complexes, with the assistance of CK1, could enhance repression without requiring a one-to-one stoichiometry with BMAL1. Instead, full repression may simply require reducing the concentration of functional CLOCK:BMAL1 at the promoter below the threshold needed to recruit the critical local concentration of coactivators.

2) Concerning the presence of CRY1(free): Data from Neurospora in constant darkness reveal that the WCC activity at its circadian peak is at least five times lower than the WCC activity in a *frq* mutant, indicating that the promoter is never fully activated. Circadian oscillations of *frq* transcription occur between fully repressed and 20% of maximal activation.

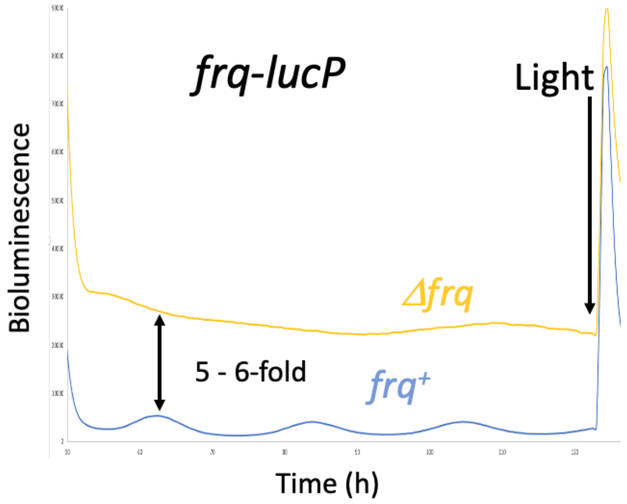

The presence of CRY1 throughout the day may serve a similar function, suggesting that CRY1(free) could define the maximal possible activation level and CRY1 : PER complexes the minimum.

3) Since the authors cannot provide data on PER1 and CRY2 (for technical reasons), I looked up the Ueda mass spectrometry paper (Narumi et al., 2016), which includes these data. I double-plotted the mass-spec and then aligned the PER2 peak at CT12 in the SCN with the PER2 peak at CT16-20 (Fig 4i) in the liver and normalized the data such that the PER2 amplitudes overlapped. I was surprised by how closely the fluorescence-based quantification of CRY1 and BMAL1 in the living SCN slices matched the mass spectrometry data from liver, with the only exception being a slightly later phase for CRY1 in the SCN.

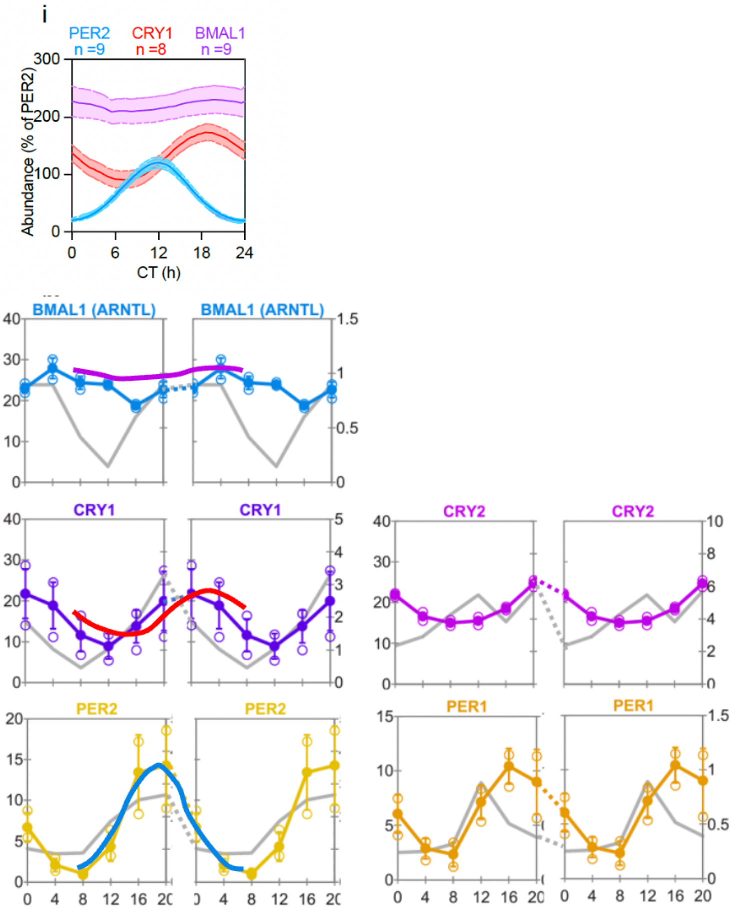

Given that PER1 and PER2, as well as CRY1 and CRY2, are expressed at similar levels in the liver, quantifying these proteins in the SCN is unlikely to affect the major conclusions of this manuscript.

Best wishes and good luck,

Michael Brunner

Referee #3:

Using genetic manipulations in SCN slice cultures Smyllie and coworkers demonstrate that the core clock proteins PER2 and CRY1 may execute both common and different, temporally staged functions in the Transcriptional Translational Feedback Loop (TTFL) of the circadian clockwork circuitry. This is in contrast with the findings revealed by biochemical studies with peripheral cell types and organs, most notable the liver. Thus, a landmark study by Aryal et al. (reference 22) revealed that in the mouse liver CRY and PER proteins are part of a ~2 MDa complex composed of about 30 polypeptides. The co-immunoprecipitation and immune-depletion experiments presented in this report suggested that the fraction of CRYs and PERs not assembled into this large complex was virtually negligible, and that the phases of PER and CRY accumulation were nearly identical. The latter had previously been demonstrated by other papers (e.g. PMID: 16675400; PMID: 17298173). Yet, the imaging studies presented here for the expression of fluorescent fusions proteins (knock-ins) with CRY1, PER2, and BMAL1 are quite convincing and carefully interpreted. The interactions between these proteins may thus indeed be different in the SCN when compared to liver. One of the strength of the study is that the fluorescence of two combinations of two different clock-fusion proteins, CRY1::Ruby3 & PER2::Venus, and CRY1::Ruby3 & Venus:: BMAL1, could be recorded in real time in SCN slice cultures. Moreover, a Cre-recombinase-induced color switch (Venus-yellow to mRuby3-red) allowed the authors to estimate the relative concentrations of the three proteins of interest (Figure 4). Obviously, this is a very important addition, since in the absence of this stoichiometric information it would have been impossible to draw conclusions regarding the possible actions of the three core clock components. The direct implications of the temporally staged expression patterns of PER2, CRY1, and BMAL1 in driving rhythmic gene expression would be difficult to assess through biochemical experiments, as the SCN only contains about 10,000 neurons. Moreover, even if ChIP-seq assays were feasible, the experiments would be confounded by the phase differences of clock protein expression in different SCN subareas. Nonetheless, the authors conduced some experiments with drugs affecting the stability of CRY1 and PER2, and the findings made in these studies support the major conclusions of the authors, namely that the different accumulation profiles of CRY1 and PER2 have functional consequences. Importantly, increasing the stabilities of CRY1 and PER2 engendered additive effects.

All in all this is an excellent piece of work, and I feel that the paper only requires editorial revisions before it can be published in EMBO Journal. Thus the authors should consider the following comments/queries.

Major issues

(1) The title is somewhat misleading, as the data extend, rather than contradict, the "canonical clock model". The biochemical data presented in reference 22 for liver are very compelling, and there is no good reason to question their validity. As shown by Kornmann et al. (PMID: 17298173), the accumulation cycles of PER and CRY proteins are driven by systemic cues (probably through the rhythmic activation of immediate early transcription factors), rather than self-sustained, cell-autonomous oscillators. Otherwise put, in mice the depletion of either BMAL1 or CLOCK in hepatocytes has little impact on circadian PER and CRY expression (PMID: 18779586; PMID: 17298173; PMID: 16675400; PMID. In contrast, BMAL1 is required to drive circadian Per2 gene expression in liver slices cultured ex vivo (PMID: 17298173). It is conceivable that the accumulation cycles of different clock proteins in cultured liver slices are similar to the ones reported here for the SCN. However, this has not been tested. The authors should discuss the difference of autonomous and systemically driven clocks in peripheral organs in their paper. To the non-informed the present title may actually suggest that PER and CRY do not participate in the establishment of a TTFL, but, similar to Kai proteins in cyanobacteria, act through posttranslational mechanisms (e.g. phosphorylation/de-phosphorylation cycles). Such claims have been made by others, but they are not supported by rigorous experiments. Something like "PER2 and CRY1 proteins drive the circadian master pacemaker in the suprachiasmatic through both common and distinct mechanisms" would be a more appropriate title.

Minor points

(2) My copy of the manuscript is devoid of page numbers.

(3) Although the PER and/or CRY and CLOCK-BMAL1 protein complexes might be different in liver (reference 22) and in the SCN, it is unlikely that they act as heterodimers. For example, in cultured fibroblasts PER and CRY proteins also assemble into large complexes (PMID: 15860628). Therefore , the term "heterodimers" should be replaced by "protein complexes" or "heterodimers in protein complexes" throughout the manuscript.

(3) Page 3, first paragraph. "indefinitely" should be replaced by "for weeks to months".

(4) Page 5. It is not clear how 3D-nuclear/cytoplasmic rations were determined. Wouldn't his require the analysis of serial sections or morphometric assays?

(5). Page 6, first paragraph. "Overall, the three proteins display different intracellular behaviors: BMAL1 is the most nuclear with a large immobile fraction, possibly indicative of DNA-bound state". However, in accessible chromatin regions of the nucleus the concentration of unspecific DNA-binding sites for any transcription factor (TF) is in the millimolar range (I shall be happy to discuss this with the authors in greater detail if so requested). Hence, most TFs are always DNA-bound, and they move by inter-segmental transfer and sliding, rather than by diffusion. Moreover, as revealed by PMID: 22981862, even when bound to specific and functional E-boxes within target genes, the dwelling time of BMAL1 is in the low second range. It is conceivable that the "slow components" reflect phase separation into liquid droplets, as has been shown for the nuclear orphan receptor REV-ERBα (another core clock component) and its co-repressor NCOR1 (PMID: 37802023).

EMBOJ-2024-119731-T

Response to Referees

We have attached a point-by-point response to the referees' comments with a colour-code using the following key:

Referees comments: black
Our response: blue
Manuscript text: *blue italics*
New Manuscript text: *new text underlined*

Referee #1:

For readers who are not familiar with this field, the reviewer recommends providing a more detailed explanation of why the present experimental results differ from previous studies about circadian clocks in the SCN. Live imaging of clock proteins has been demonstrated in previous studies, such as "Real-Time Recording of Circadian Per1 and Per2 Expression in the Suprachiasmatic Nucleus of Freely Moving Rats. Yamaguchi Y et al. J Biol Rhythms. 2016 Feb;31(1):108-11." Would it be possible to discuss in detail the distinctive features and advantages of the present study's methodology compared to previous works?

To address this suggestion, we have included additional detail about the advantages of our approach to live imaging of circadian clock proteins within the introduction section. Here, we specifically highlight the differences of our approach compared to that of *Yamaguchi et al. 2016* (transcriptional luciferase report) and *Yoo et al. 2004* (translational luciferase report).

"Luciferase-based bioluminescence approaches have enabled live-imaging of both transcriptional (Yamaguchi et al. 2016) and translational (Yoo et al. 2004) temporal dynamics of SCN clock proteins. These are, however, indirect enzymatic readouts of protein abundance with limited spatial resolution.

To develop this further, we employed a fluorescent-fusion, multi-channel approach to image SCN clock proteins directly and simultaneously. Organotypic SCN slices from mice……were used to visualise measure and manipulate, pharmacologically, the behaviour of the individual proteins in their native setting*"*

Figure 5i and j, 6i and j. In the treatment by PF670462 and KL 101, the dose-dependent decrease in Amplitude and Baseline are observed in Figure 5i and j, 6i and j. The reviewer considers that the cytotoxic effects of the stabilizer drugs might cause the decrease. Would it be possible to rule out these concerns based on previous literature and available data?

We agree with the Reviewer that cytotoxicity is an important consideration for any pharmacological manipulation. We would like to highlight two key points relating to our own data. First, the effects on period, amplitude and baseline on *Cry1*-Luc report are

completely reversible on washout of the drugs. Second, the effect on amplitude and baseline are specific to *Cry1*-Luc and are not observed for PER2::Luc. Together, these argue against cytotoxic effects, which would have a pan-reduction in both luciferase-based reports.

With regards to previous literature, PF670462 has been extensively used on SCN slices at the same dose ranges that we used in this study (Meng et al. 2010; Patton et al. 2016; Smyllie et al. 2016) without any suggestion of cytotoxicity. Additionally, Janovska et al. 2018, used PF670462 in primary cell cultures at concentrations higher than we used, but with no decrease, compared to controls, in the expression of housekeeping protein used as an indicator of cellular health. For KL101, Miller et al. 2020 published a viability assay for KL101, confirming 100% viability of U2OS cells for the complete range of concentrations that we used.

We have now included the following text to the methods section of the manuscript, including highlighting previous literature:

"*To ensure that our measured effects were "on target" and not related to cytotoxicity, only doses where the measured effects were immediately and fully reversible on washout of the drug were used. Dose ranges for protein stabiliser drugs were selected based on previous literature (Meng et al. 2010; Patton et al. 2016; Miller et al. 2020), where no cytotoxic effects were reported.*"

Figure 1 d. The reviewer recommends explaining what the blue color represents in Merge Figures.

Thank you for this recommendation. We have added "*counterstained with DAPI (blue)*" to the figure legends of Figure 1B and 1D.

Figure 3 c. To assist readers who may not be familiar with SCN anatomy, the reviewer suggests including anatomical orientation markers in the figure. Specifically, it is helpful to have directional indicators that clearly show which side is dorsal or ventral.

We have taken the Reviewer's recommendation and added dorsal (d), ventral (v), medial (m) and lateral (l) labels to Fig 3D but also in Fig 1A, the first time an image of the SCN appears. We have included a key in the figure legend for the d, v, m, l labels.

Referee #2:

Minor points:

In the final paragraph of the Introduction, PER and CRY should be specified as PER2 and CRY1, right?

Thank you, you are correct. We have updated the text to say "PER2" and "CRY1".

In Fig 4A right panel: the BMAL1 + CHX trace does not approach the dotted baseline. Rather, by extrapolation, the BMAL data seems to cross the dotted line.

Thank you for spotting this. It appears that the dotted line was misplaced in drafting the figure and so we have updated Figure 4A where the dotted line now matches the projected baseline more closely. For this point, we also wanted to reiterate the limitations of using CHX to determine the baseline of BMAL1 due to its very long half-life. We use an extrapolation of a one-phase decay curve fit, whose projection would go beyond the end of this graph. Details on the curve-fits and baseline analysis are included in the methods section.

The authors show in Figures 5 - 7 that inhibitors, PF670 and KL101, lengthened the descending phase of their targets, PERs and CRY1, respectively but stretched the rhythms of the corresponding other partner in a symmetrical manner. Application of both inhibitors lengthened period in an additive manner. The authors suggest that actions of increased PER and CRY1 stability are independent. I totally agree with this interpretation. However, as statement in the paper this could potentially lead to some confusion. We know that PERs do not repress without CRYs. I guess what the authors mean is that the action of the PER:CRY complex is governed by PER stability and not by CRY, which is protected from degradation when bound to PERs, while KL101 acts only on free CRY.

Thank you for highlighting this point and agree that our truncated phrasing of this could be confusing when the effects are more nuanced. We have therefore amended references to PER and CRY independent **actions** to be more specific and instead refer to PER:CRY heterodimer and $CRY1_{free}$ when appropriate.

Penultimate paragraph of results:

“Dual application lengthened period significantly further than did either drug alone (Fig. 7b), thereby confirming additive, independent ~~actions~~ of increased PER and CRY1 stability.”

Replaced with:

“Dual application lengthened period significantly further than did either drug alone (Fig. 7B), thereby confirming additive and independent effects of increased PER and CRY1 stability”

Last paragraph of results:

“Taken together, specific pharmacological perturbations of PER and CRY1 stability reveal independent and additive ~~actions~~ of PER and CRY1 on the TTFL, that are consistent with the differing temporal profiles of (early) PER2 and (late) CRY1 abundance.”

Replaced with:

"Taken together, specific pharmacological perturbations of PER and CRY1 stability reveal independent control of their stabilities that have additive effects on the TTFL, that are consistent with the differing temporal profiles of (early) PER2 and (late) CRY1 abundance."

Penultimate paragraph of discussion:

"Finally, joint application of sub-saturating doses of PF670462 and KL101 had an additive effect on lengthening SCN period, indicative ~~of independent actions~~ of stabilised PER and CRY1. This provides mechanistic insight into genetic studies of circadian behaviour and SCN function that showed additive effects of mutations in key regulators within PER and CRY degradation pathways. Thus, PER and CRY1 (and BMAL1) stabilities are independent of each other, and PER and CRY1 proteins ~~act independently~~ within the SCN TTFL."

Replaced with:

"Finally, joint application…indicative of independent effects of PER and CRY1 stabilisation. This provides mechanistic…degradation pathways. Specifically, the dominant effect of PER2 stabilisation is to prolong the PER:CRY heterodimer activity phase in the early circadian night, whereas CRY1 stabilisation boosts the free-CRY1 activity phase in the late circadian night. Thus, PER and CRY1 (and BMAL1) stabilities are independent of each other, and PER and CRY1 proteins have different, complementary actions within the SCN TTFL."

Last paragraph of discussion:

"~~PER2 and~~ CRY1 have independent behaviours outside of the PER:CRY heterodimer"

Replaced with:

"CRY1 has additional independent behaviours outside of the PER:CRY heterodimer"

Suggestions and comments:

I believe the authors may be overly critical in dismissing the "old clock model"
… both PER:CRY complexes and CRY alone are capable of interacting with the BMAL1:CLOCK complex, and I am sure the authors fully agree with this. However, what this manuscript shows in an impressive and convincing manner is that the repression cannot be mediated by a simple stochiometric mechanism
…Hence, the prevalent dual mode repression model must be substantially modified, taking into account the quantitative data presented here. Yet, the authors might consider revising their title to better reflect this updated perspective by softening the statement.

Yes, a very fair point. We accept that our title could be interpreted quite harshly and we therefore should more carefully describe the relationship between our new data and the canonical clock model. We have therefore amended the title of our paper to:

"Quantitative measures of clock protein dynamics in the SCN extends the circadian time-keeping model"

(paraphrased comment below)
I believe our field still adheres too strongly to an outdated, stoichiometry based model of transcription regulation…The current understanding seems to be shifting toward a model where highly dynamic hubs of transcription factors transiently assemble and accumulate at the promoter…An important aspect of transcription regulation likely involves modulation of the poised state…Since either CRY1 or CRY1:PERs are present throughout the day, promoters are likely never fully active. The ratio of CRY1 to BMAL1:CLOCK within a TF hub at the promoter could determine the degree of repression (always high), without relying on a strict 1:1 stoichiometry. Rhythmically accumulating PER:CRY complexes, with the assistance of CK1, could enhance repression without requiring a one-to-one stoichiometry with BMAL1. Instead, full repression may simply require reducing the concentration of functional CLOCK:BMAL1 at the promoter below the threshold needed to recruit the critical local concentration of coactivators.

Thank you for highlighting this point. We acknowledge that we refer to protein stoichiometries and ratios, particularly with reference to Figure 4 and Extended Figure 9. Our rationale for expressing the proteins and complexes as ratios in this simplified manner was to aid interpretation of how the total amounts of each species relate to one another over time. We agree, however, that static, strict stoichiometries are an oversimplification of the dynamic nature of clock proteins, whether within their large megadalton protein complexes or highly dynamic transcriptional hubs at promoters. We have therefore included the following text in the second paragraph of the discussion to make this point:

"The dynamic nature of these clock proteins strongly suggests that circadian regulation at E-boxes is not a static and strict stoichiometric phenomenon but, instead, should be considered as a dynamic hub of transiently associating and dissociating transcription factors."

Concerning the presence of CRY1(free): Data from Neurospora in constant darkness reveal that the WCC activity at its circadian peak is at least five times lower than the WCC activity in a frq mutant, indicating that the promoter is never fully activated. Circadian oscillations of frq transcription occur between fully repressed and 20% of maximal activation. The presence of CRY1 throughout the day may serve a similar function, suggesting that **CRY1(free) could define the maximal possible activation level and CRY1:PER complexes the minimum.**

This is an excellent thought that we did not pursue sufficiently in our original thoughts. We have now made this more explicit by amending Extended Figure 9 (re-labelled as Extended View Figure 5 to conform to EMBO-J Figure guidelines) and adding this point to the discussion section:

"the persistent presence of CRY1 suggests that the potential for E-box repression remains throughout the day, either as PER2:CRY1 or $CRY1_{free}$, which could be modulated by the presence of corepressors. Further to this, the level of inhibition of $CRY1_{free}$ could set the maximal level of E-box activation, whereas the abundance of PER2, which limits the availability of PER2:CRY1, may therefore set the minimum, i.e., less PER2 would give a higher minimum."

Since the authors cannot provide data on PER1 and CRY2 (for technical reasons), I looked up the Ueda mass spectrometry paper (Narumi et al., 2016), which includes these data. I double-plotted the mass-spec and then aligned the PER2 peak at CT12 in the SCN with the PER2 peak at CT16-20 (Fig 4i) in the liver and normalized the data such that the PER2 amplitudes overlapped. I was surprised by how closely the fluorescence-based quantification of CRY1 and BMAL1 in the living SCN slices matched the mass spectrometry data from liver, with the only exception being a slightly later phase for CRY1 in the SCN.

This meta-analysis of our data compared to Narumi et al. 2016 it is very insightful. It prompted us to go back to our primary data files and repeat this more formally and indeed the internal relative phasing between PER2, CRY1 and BMAL1 were similar for the liver data compared with the SCN. This gives us some confidence that the addition of PER1 and CRY2 to the mix would not alter the conclusions we have made here. Thank you for this.

Referee #3:

Major issues:

1) The title is somewhat misleading, as the data extend, rather than contradict, the "canonical clock model"…. The authors should discuss the difference of autonomous and systemically driven clocks in peripheral organs in their paper. To the non-informed the present title may actually suggest that PER and CRY do not participate in the establishment of a TTFL, but, similar to Kai proteins in cyanobacteria, act through posttranslational mechanisms (e.g. phosphorylation/de-phosphorylation cycles). Such claims have been made by others, but they are not supported by rigorous experiments.

Thank you for your comments regarding the title of the paper. Confusion about the title was also raised by Reviewer 2, thus we amended it as discussed above. In addition, we shall clarify autonomous versus systemically driven clocks by adding the following text (new text underlined below) within the introduction section:

"This timing signal is conveyed via neural and neuroendocrine links to co-ordinate subordinate cell-autonomous clocks across tissues, thereby orchestrating daily rhythms of physiology and sleep-wakefulness[3]. Importantly, whereas circadian rhythms of subordinate clocks in peripheral tissues damp rapidly when isolated from SCN-derived and systemic cues, organotypic SCN slice cultures can maintain autonomous, high amplitude oscillations for many weeks and months, in the absence of systemic cues."

Minor points:

(2) My copy of the manuscript is devoid of page numbers.

We are sorry that this is the case, this must have got missed on submission. They are now added.

(3) Although the PER and/or CRY and CLOCK-BMAL1 protein complexes might be different in liver (reference 22) and in the SCN, it is unlikely that they act as heterodimers. For example, in cultured fibroblasts PER and CRY proteins also assemble into large complexes (PMID: 15860628). Therefore, the term "heterodimers" should be replaced by "protein complexes" or "heterodimers in protein complexes" throughout the manuscript.

We agree with the Reviewer that it is unlikely that PER2 and CRY1 act as pure heterodimers in isolation in the SCN and rather form heterodimeric interactions that ultimately assemble into large protein complexes, similarly to those detected in mouse liver. We have updated the manuscript to be explicit about this. At the first reference to the PER2:CRY1 heterodimer in the results section, we include the following statement:

"…formation of PER2:CRY1, where these heterodimers exist within larger protein assemblies (Aryal *et al*, 2017)"

We also replace references to "heterodimers" with "heteromeric protein complexes" or "PER2:CRY1 complex" where relevant.

(3) Page 3, first paragraph. "indefinitely" should be replaced by "for weeks to months".

We have updated the manuscript to replace "*indefinitely*" with "*many weeks to months*."

(4) Page 5. It is not clear how 3D-nuclear/cytoplasmic rations were determined. Wouldn't this require the analysis of serial sections or morphometric assays?

I think there is a misunderstanding of the technique we used. The nuclear:cytoplasmic ratio measures were conducted within 2D confocal images of cells within fixed SCN slices. Thus, the measures were taken across a single 2D plane for each cell. Although the plane for each cell will be slightly different, the measures are not in the cell periphery, so that nuclear and plasma membrane boundaries are clearly demarcated. We have made the following changes to the wording to the methods section to clarify this:

Nucleus:Cytoplasmic (Nuc:Cyto) Ratio
Nuc:Cyto ratios were measured in 2D confocal images of fixed SCN slices. Fluorescence intensity was measured within FIJI software (NIH, USA). All measures were background-corrected using fluorescence intensity outside of the SCN area. ROIs were selected for nucleus and cytoplasm for each cell within 2D images. The Nuc:cyto ratio was then calculated for the background-subtracted intensities and expressed as percentages.

5). Page 6, first paragraph. "Overall, the three proteins display different intracellular behaviors: BMAL1 is the most nuclear with a large immobile fraction, possibly indicative of DNA-bound state". However, in accessible chromatin regions of the nucleus the concentration of unspecific DNA-binding sites for any transcription factor (TF) is in the millimolar range… most TFs are always DNA-bound, and they move by inter-segmental transfer and sliding, rather than by diffusion. Moreover, as revealed by PMID: 22981862, even when bound to specific and functional E-boxes within target genes, the dwelling time

of BMAL1 is in the low second range. It is conceivable that the "slow components" reflect phase separation into liquid droplets, as has been shown for the nuclear orphan receptor REV-ERBα (another core clock component) and its co-repressor NCOR1 (PMID: 37802023).

Thank you for the Reviewer's insight into the details about TF mobility. We agree with the Reviewer that inter-segmental transfer and sliding are the primary modes of mobility for TFs once bound to DNA. We have included additional text to emphasise that BMAL1 is primarily DNA bound:

"Overall, the three proteins display different intracellular behaviours: BMAL1 is the most nuclear with a large immobile fraction, which likely represents its predominantly DNA-bound state".

The Reviewer makes an interesting point about phase separation in liquid droplets. We have thus included new text at the point where the slow components are first mentioned (new next underlined below):

"This indicates that, like PER2 and BMAL1, CRY1 molecules form two mobility pools referred to as $CRY1_{fast}$ (~30%), and $CRY1_{slow}$ (~20%) and an "immobile" (or very slowly moving) pool, $CRY1_{immobile}$ (~50%), represented by fluorescence that did not recover over the 90 seconds course of the experiment. The presence of a slow mobility component may represent molecules present in phase-separated liquid droplets, which have been detected for the clock proteins REV-ERBα and NCOR1 (PMID: 37802023)".

1st Revision - Editorial Decision **20th Feb 2025**

Dear Dr. Hastings,

Thank you for submitting your revised manuscript (EMBOJ-2024-119731R) to The EMBO Journal, and for your patience during re-review. Your manuscript has now been seen by two of the original referees who had also assessed the previous version, and we have now received their comments, which you can find below.

I am very pleased to say that the referees recognize that the initially raised concerns have been fully addressed, and that the revised manuscript is interesting and makes a significant contribution to the understanding of the circadian clock. In light of this supportive input, I am glad to inform you that your manuscript has in principle been accepted for publication in The EMBO Journal - congratulations on an excellent work!

From the editorial side, there are a few corrections and changes that we need from you to make in a final version of your manuscript. Please include in your resubmission a brief cover letter describing any changes to the manuscript:

- The author contributions statement should be removed from the manuscript file. Instead, we use CRediT to specify the contributions of each author in the journal submission system. Please feel free to use the free text box to provide more detailed descriptions during submission. See also our guide to authors for more information: https://www.embopress.org/page/journal/14602075/authorguide#authorshipguidelines.

- Please provide the information on materials availability in the respective paragraphs of your Methods and Protocols, where the generation of the new mouse line and AAV construct are described. The "Materials availability" section can now be removed from the manuscript.

- Please move the statement currently under the heading "Lead contact" to the end of your "Data availability" statement, and remove the section "Lead contact" from the manuscript.

- Please correct the callouts for the Appendix figures to "Appendix Figure Sx" (the "S" is currently missing), throughout your manuscript.

- We noticed that callouts for Fig. 5H and 7H are missing; please make sure that all Figure panels are called out in the final version of your manuscript.

- Please state details of the authority granting ethics approval of your experiments involving animals (including the reference number of approval) in paragraph "Animals" of your Methods and Protocols.

- Please add the heading "Appendix for" followed by the manuscript' title on the first page of your Appendix PDF file. This should be followed by a brief Table of Contents (including page numbers) listing all Appendix items.

- Please remove the Reagents and Tools table from your main manuscript file and upload it instead to our manuscript tracking system as an individual file (choosing the file type "Reagent Table") using the template you can find in our author guide: https://www.embopress.org/page/journal/14602075/authorguide#structuredmethods. The table listing primers and sequences can be incorporated in your Reagents and Tools table.

- Please upload your synopsis image in JPG/TIFF format (it is currently in PDF format).

- During our routine pre-acceptance checks, our data editors have raised the following queries:
1. Please note that the exact p values are not provided in the legends of Figures 3B, 4B, H, M; 5C, H; 6C, H, I, J; 7B, D, H; EV2 D, EV3 D, EV4 B, D; Supplementary figure(s) 2C, 3D, F; 5C.
2. Please indicate the statistical test used for data analysis in the legends of Figures 5C, 6C, supplementary figure(s) 2B, D.
3. Please note that the error bars are not defined in the legends of Figures 2D, 3B, supplementary figures 2D, 4B, C.
Some of this information is already provided in your Statistics Table (which should be named "Table 1" and be accompanied by a brief caption). Please make sure that all requested information is provided either in the Figure legends or in this Table in your revised manuscript.

- During our standard figure checks, we detected two cases of possible cell reuse that is not detailed in the Figure legends:
1. Possible cell reuse between Figure 1B and Appendix Figure S1A.
2. Possible cell reuse between Figure 2B AND Figure 3G.
Please double-check these Figure pairs and correct them or, if the reuse is justified and intentional, please describe it in the respective Figure legends.

- The manuscript's section order should be corrected as follows: Title page - Abstract & Keywords - Introduction - Results - Discussion - Methods - Data Availability - Acknowledgements - Disclosure and Competing Interests Statement - References -

Figure Legends - Table with legend - Expanded View Figure Legends.

Please also note that as part of the EMBO publications' Transparent Editorial Process, The EMBO Journal publishes online a Peer Review File along with each accepted manuscript. This File will be published in conjunction with your paper and will include the referee reports, your point-by-point response and all pertinent correspondence relating to the manuscript. You can opt out of this by letting the editorial office know (contact@embojournal.org). If you do opt out, the Peer Review File link will point to the following statement: "No Peer Review File is available with this article, as the authors have chosen not to make the review process public in this case."

We look forward to seeing a final version of your manuscript as soon as possible. Please let us know if you have any questions and use this link to submit your revision: https://emboj.msubmit.net/cgi-bin/main.plex.

Best wishes,

Ioannis

Ioannis Papaioannou, PhD
Editor, The EMBO Journal
i.papaioannou@embojournal.org

Referee #2:

The authors have fully addressed my comments and suggestions. I am of the opinion that the revised manuscript makes a significant contribution to the understanding of the circadian clock.

Referee #3:

In my review of the original submission I have only requested editorial revisions. In the revised version of their manuscript, the authors have now addressed my queries in a satisfactory fashion, and I thus recommend that this interesting and carefully conducted study is now accepted for publication in EMBO Journal.

All editorial and formatting issues were resolved by the authors.

2nd Revision - Editorial Decision 3rd Mar 2025

Dear Dr. Hastings,

Congratulations on an excellent work! I am very pleased to inform you that your manuscript has now been accepted for publication in The EMBO Journal. Thank you for your thorough responses to the referees' comments, and for addressing our editorial and formatting requests.

Your manuscript will be processed for publication by EMBO Press. It will be copy edited and you will receive page proofs prior to publication. Please note that you will be contacted by Springer Nature Author Services to complete licensing and payment information.

You may qualify for financial assistance for your publication charges - either via a Springer Nature fully open access agreement or an EMBO initiative. Check your eligibility: https://www.embopress.org/page/journal/14602075/authorguide#chargesguide

Should you be planning a Press Release on your article, please get in contact with embo_production@springernature.com as early as possible in order to coordinate publication and release dates.

If you have any questions, please do not hesitate to contact the Editorial Office. Thank you for your contribution to The EMBO Journal. Working with you has been a pleasure!

Best regards,

Ioannis

Ioannis Papaioannou, PhD
Editor, The EMBO Journal
i.papaioannou@embojournal.org

>>> Please note that it is The EMBO Journal policy for the transcript of the editorial process (containing referee reports and your response letter) to be published as an online supplement to each paper. If you do NOT want this, you will need to inform the Editorial Office via email immediately. More information is available here: https://www.embopress.org/transparent-process#Review_Process

** Click here to be directed to your login page: https://emboj.msubmit.net

EMBO Press Author Checklist

Corresponding Author Name: Michael H Hastings	
Journal Submitted to: The EMBO Journal	
Manuscript Number: EMBOJ-2024-119731-T	

USEFUL LINKS FOR COMPLETING THIS FORM
The EMBO Journal - Author Guidelines
EMBO Reports - Author Guidelines
Molecular Systems Biology - Author Guidelines
EMBO Molecular Medicine - Author Guidelines

Reporting Checklist for Life Science Articles (updated January
This checklist is adapted from Materials Design Analysis Reporting (MDAR) Checklist for Authors. MDAR establishes a minimum set of requirements in transparent reporting in the life sciences (see Statement of Task: 10.31222/osf.io/9sm4x). Please follow the journal's guidelines in preparing your
Please note that a copy of this checklist will be published alongside your article.

Abridged guidelines for figures
1. Data
The data shown in figures should satisfy the following conditions:
- the data were obtained and processed according to the field’s best practice and are presented to reflect the results of the experiments in an accurate and unbiased manner.
- ideally, figure panels should include only measurements that are directly comparable to each other and obtained with the same assay.
- plots include clearly labeled error bars for independent experiments and sample sizes. Unless justified, error bars should not be shown for technical
- if n<5, the individual data points from each experiment should be plotted. Any statistical test employed should be justified.
- Source Data should be included to report the data underlying figures according to the guidelines set out in the authorship guidelines on Data

2. Captions
Each figure caption should contain the following information, for each panel where they are relevant:
- a specification of the experimental system investigated (eg cell line, species name).
- the assay(s) and method(s) used to carry out the reported observations and measurements.
- an explicit mention of the biological and chemical entity(ies) that are being measured.
- an explicit mention of the biological and chemical entity(ies) that are altered/varied/perturbed in a controlled manner.
- the exact sample size (n) for each experimental group/condition, given as a number, not a range;
- a description of the sample collection allowing the reader to understand whether the samples represent technical or biological replicates (including how many animals, litters, cultures, etc.).
- a statement of how many times the experiment shown was independently replicated in the laboratory.
- definitions of statistical methods and measures:
  - common tests, such as t-test (please specify whether paired vs. unpaired), simple χ2 tests, Wilcoxon and Mann-Whitney tests, can be unambiguously identified by name only, but more complex techniques should be described in the methods section;
  - are tests one-sided or two-sided?
  - are there adjustments for multiple comparisons?
  - exact statistical test results, e.g., P values = x but not P values < x;
  - definition of ‘center values’ as median or average;
  - definition of error bars as s.d. or s.e.m.

Please complete ALL of the questions below.
Select "Not Applicable" only when the requested information is not relevant for your study.

Materials

Newly Created Materials	Information included in the manuscript?	In which section is the information available? (Reagents and Tools Table, Materials and Methods, Figures, Data Availability Section)
New materials and reagents need to be available; do any restrictions apply?	Yes	Materials and Methods, Materials Availability Section

Antibodies	Information included in the manuscript?	In which section is the information available? (Reagents and Tools Table, Materials and Methods, Figures, Data Availability Section)
For **antibodies** provide the following information:<br>- Commercial antibodies: RRID (if possible) or supplier name, catalogue number and or/clone number<br>- Non-commercial: RRID or citation	Not Applicable	

DNA and RNA sequences	Information included in the manuscript?	In which section is the information available? (Reagents and Tools Table, Materials and Methods, Figures, Data Availability Section)
**Short novel DNA or RNA including primers, probes**: provide the sequences.	Yes	Materials and Methods

Cell materials	Information included in the manuscript?	In which section is the information available? (Reagents and Tools Table, Materials and Methods, Figures, Data Availability Section)
**Cell lines:** Provide species information, strain. Provide accession number in repository **OR** supplier name, catalog number, clone number, and/**OR** RRID.	Not Applicable	
**Primary cultures:** Provide species, strain, sex of origin, genetic modification status.	Yes	Tools Table, Materials and Methods
Report if the cell lines were recently **authenticated** (e.g., by STR profiling) and tested for mycoplasma contamination.	Not Applicable	

Experimental animals	Information included in the manuscript?	In which section is the information available? (Reagents and Tools Table, Materials and Methods, Figures, Data Availability Section)
**Laboratory animals or Model organisms:** Provide species, strain, sex, age, genetic modification status. Provide accession number in repository **OR** supplier name, catalog number, clone number, **OR** RRID.	Yes	Tools Table, Materials and Methods
**Animal observed in or captured from the field:** Provide species, sex, and age where possible.	Not Applicable	
Please detail **housing and husbandry conditions**.	Yes	Materials and Methods

Plants and microbes	Information included in the manuscript?	In which section is the information available? (Reagents and Tools Table, Materials and Methods, Figures, Data Availability Section)
**Plants:** provide species and strain, ecotype and cultivar where relevant, unique accession number if available, and source (including location for collected wild specimens).	Not Applicable	
**Microbes:** provide species and strain, unique accession number if available, and source.	Not Applicable	

Human research participants	Information included in the manuscript?	In which section is the information available? (Reagents and Tools Table, Materials and Methods, Figures, Data Availability Section)
If collected and within the bounds of privacy constraints report on age, sex and gender or ethnicity for all study participants.	Not Applicable	

Core facilities	Information included in the manuscript?	In which section is the information available? (Reagents and Tools Table, Materials and Methods, Figures, Data Availability Section)
If your work benefited from core facilities, was their service mentioned in the acknowledgments section?	Yes	Acknowledgements

Design

Study protocol	Information included in the manuscript?	In which section is the information available? (Reagents and Tools Table, Materials and Methods, Figures, Data Availability Section)
If study protocol has been **pre-registered, provide DOI in the manuscript**. For clinical trials, provide the trial registration number **OR** cite DOI.	Not Applicable	
Report the **clinical trial registration number** (at ClinicalTrials.gov or equivalent), where applicable.	Not Applicable	

Laboratory protocol	Information included in the manuscript?	In which section is the information available? (Reagents and Tools Table, Materials and Methods, Figures, Data Availability Section)
Provide DOI OR other citation details if **external detailed step-by-step protocols** are available.	Not Applicable	

Experimental study design and statistics	Information included in the manuscript?	In which section is the information available? (Reagents and Tools Table, Materials and Methods, Figures, Data Availability Section)
Include a statement about **sample size** estimate even if no statistical methods were used.	Yes	Figures and Materials and Methods
Were any steps taken to minimize the effects of subjective bias when allocating animals/samples to treatment (e.g. **randomization procedure**)? If yes, have they been described?	Not Applicable	
Include a statement about **blinding** even if no blinding was done.	Not Applicable	
Describe **inclusion/exclusion criteria** if samples or animals were excluded from the analysis. Were the criteria pre-established?<br><br>If sample or data points were omitted from analysis, report if this was due to attrition or intentional exclusion and provide justification.	Not Applicable	
For every figure, are **statistical tests** justified as appropriate? Do the data meet the assumptions of the tests (e.g., normal distribution)? Describe any methods used to assess it. Is there an estimate of variation within each group of data? Is the variance similar between the groups that are being statistically compared?	Yes	

Sample definition and in-laboratory replication	Information included in the manuscript?	In which section is the information available? (Reagents and Tools Table, Materials and Methods, Figures, Data Availability Section)
In the figure legends: state number of times the experiment was **replicated** in laboratory.	Yes	Figure legends and Materials and Methods
In the figure legends: define whether data describe **technical or biological replicates**.	Yes	Figure legends and Materials and Methods

Ethics

Ethics	Information included in the manuscript?	In which section is the information available? (Reagents and Tools Table, Materials and Methods, Figures, Data Availability Section)
Studies involving **human participants**: State details of **authority granting ethics approval** (IRB or equivalent committee(s), provide reference number for approval.	Not Applicable	
Studies involving **human participants**: Include a statement confirming that **informed consent** was obtained from all subjects and that the experiments conformed to the principles set out in the WMA Declaration of Helsinki and the Department of Health and Human Services Belmont Report.	Not Applicable	
Studies involving **human participants:** For publication of **patient photos**, include a statement confirming that consent to publish was obtained.	Not Applicable	
Studies involving experimental **animals**: State details of **authority granting ethics approval** (IRB or equivalent committee(s), provide reference number for approval. Include a statement of compliance with ethical regulations.	Yes	Materials and Methods
Studies involving **specimen and field samples:** State if relevant **permits** obtained, provide details of authority approving study; if none were required, explain why.	Not Applicable	

Dual Use Research of Concern (DURC)	Information included in the manuscript?	In which section is the information available? (Reagents and Tools Table, Materials and Methods, Figures, Data Availability Section)
Could your study fall under dual use research restrictions? Please check biosecurity documents and list of **select agents and toxins** (CDC): https://www.selectagents.gov/sat/list.htm	Not Applicable	
If you used a select agent, is the security level of the lab appropriate and reported in the manuscript?	Not Applicable	
If a study is subject to dual use research of concern regulations, is the name of the **authority granting approval and reference number** for the regulatory approval provided in the manuscript?	Not Applicable	

Reporting

The MDAR framework recommends adoption of discipline-specific guidelines, established and endorsed through community initiatives. Journals have their own policy about requiring specific guidelines and recommendations to complement MDAR.

Adherence to community standards	Information included in the manuscript?	In which section is the information available? (Reagents and Tools Table, Materials and Methods, Figures, Data Availability Section)
State if relevant guidelines or checklists (e.g., **ICMJE, MIBBI, ARRIVE, PRISMA**) have been followed or provided.	Not Applicable	
For **tumor marker prognostic studies,** we recommend that you follow the **REMARK** reporting guidelines (see link list at top right). See author guidelines, under 'Reporting Guidelines'. Please confirm you have followed these guidelines.	Not Applicable	
For **phase II and III randomized controlled trials**, please refer to the **CONSORT** flow diagram (see link list at top right) and submit the CONSORT checklist (see link list at top right) with your submission. See author guidelines, under 'Reporting Guidelines'. Please confirm you have submitted this list.	Not Applicable	

Data Availability

Data availability	Information included in the manuscript?	In which section is the information available? (Reagents and Tools Table, Materials and Methods, Figures, Data Availability Section)
Have **primary datasets** been deposited according to the journal's guidelines (see 'Data Deposition' section) and the respective accession numbers provided in the Data Availability Section?	Yes	Data Availability Section
Were **human clinical and genomic datasets** deposited in a public access-controlled repository in accordance to ethical obligations to the patients and to the applicable consent agreement?	Not Applicable	
Are **computational models** that are central and integral to a study available without restrictions in a machine-readable form? Were the relevant accession numbers or links provided?	Not Applicable	
If publicly available data were reused, provide the respective **data citations in the reference list**.	Not Applicable	
